# *P53*-Related Anticancer Activities of *Drimia calcarata* Bulb Extracts Against Lung Cancer

**DOI:** 10.3389/fmolb.2022.876213

**Published:** 2022-06-13

**Authors:** K. Laka, Z. Mbita

**Affiliations:** Department of Biochemistry, Microbiology and Biotechnology, University of Limpopo, Polokwane, South Africa

**Keywords:** lung cancer, *Drimia calcarata*, apoptosis, cell cycle, p53, STATs, anticancer

## Abstract

Current lung cancer treatment strategies are ineffective, and lung cancer cases continue to soar; thus, novel anticancer drugs and targets are needed, and medicinal plants are promising to offer better alternatives. This study was aimed at analysing two *p53* splice variants during the potential anticancer activities of *Drimia calcarata* (*Dc*) methanol and water extracts against different human lung cancer cell lines of varying *p53* mutation status, and these included mutant H1573 and mutant H1437 and *p53*-wild type (A549) cells. The anticancer activities of the *Dc* extracts were assessed by establishing the cytotoxic effect and the apoptosis-inducing capacity of these extracts, using the MTT assay and Annexin V analysis, respectively, with the latter confirmed using fluorescence microscopy. The molecular mechanisms induced by these extracts were further evaluated using cell cycle analysis and RT-PCR. Both extracts demonstrated safety against noncancerous lung MRC-5 fibroblasts and exhibited significant anticancer potency (*p* < 0.001) against the H1437 (IC_50_ values: 62.50 μg/ml methanol extract and 125 μg/ml WE), H1573 (IC_50_ value: 125 μg/ml for both extracts) and A549 (IC_50_ value: 500 μg/ml ME). The water extract had no effect on the viability of A549 cells. Treated H1437 cells underwent *p53*-dependent apoptosis and S-phase cell cycle arrest while H1573 treated cells underwent *p53*-independed apoptosis and G0/G1 cell cycle arrest through upregulation of *p21* mRNA expression levels. The expression levels of *STAT1*, *STAT3*, *STAT5A* and *STAT5B* genes increased significantly (*p* < 0.001) following the treatment of H1573 cells with ME and WE. Treatment of H1437 cells with ME upregulated the *STAT1*, *STAT3*, *STAT5A* and *STAT5B* mRNAs. Our results indicate that the proliferative inhibitory effect of *D. calcarata* extracts on A549 and H1573 cells is correlated with the suppression of *Bcl-2*, *STAT3* and *STAT5B* while that is not the case in H1437 cells. Thus, our results suggest that the dysregulation of anti-apoptotic molecules *Bcl-2*, *STAT3*, *STAT5A* and *STAT5B* in H1437 may play a role in cancer cell survival, which may consequently contribute to the development of *p53*-mutated non-small human lung cancer. Our results indicate that *D. calcarata* is a promising source of anticancer agents for the treatment of p53-mutant human non-small lung cancer cells than the p53-wild type human non-small lung cancer cells.

## Graphical Abstract

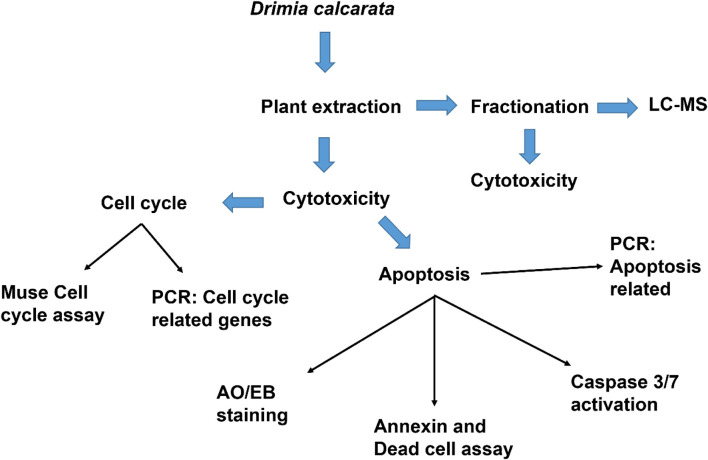

## 1 Introduction

Cancer is one of the leading cause of mortality, worldwide, second only to cardiovascular diseases (Yusuf et al., 2020; Sung et al., 2021). It is a challenge in African countries, generally, due to limited funding available to deal with the cancer epidemic and awareness, which should be prioritised with more possible resources channelled towards cancer prevention and treatment (Kuete and Efferth, 2015). Lung cancer remains the leading cause of cancer death, with an estimated 1.8 million deaths (18%), followed by colorectal (9.4%), liver (8.3%), stomach (7.7%), and female breast (6.9%) cancers (Wu et al., 2019; Sung et al., 2021). In South Africa, lung cancer is the second most common cancer among men and the sixth most common cancer among women, according to Cancer Association of South Africa (CANSA). There are three basic types of lung disease. It is essential to understand which type of cancer one has since the type of cancer subtype impacts on the available treatment options and outlook. The lung cancer subtypes include non-small cell lung cancer, small cell lung cancer, and those characterised by carcinoid tumours (Jemal et al., 2008). Worldwide, little is known about the lung cancer subtypes mortality rates as the death certificates do not record the subtype information (Howlader et al., 2020). In terms of treatment, lung cancer is typically treated by surgery, chemotherapy, and radiation. In patients with advanced disease or relapse, surgery remains the most effective therapeutic option. However, new drugs are being investigated that target specific components of tumour cells, improving survival.

Even with technological improvement, radiation still affects, to a certain extent, healthy tissues surrounding and adjacent to tumours, for example the highly radiosensitive lung tissue (Schaue et al., 2015). Lung cancer treatment is changing significantly thanks to directed therapies. Therapies in this category include drugs that target driver mutations, those that inhibit immune checkpoint molecules, and those that target presumptive important molecules in malignant growth and survival (Hirsch et al., 2016). Cetuximab and necitumumab, monoclonal antibodies directed against the EGFR, are among these therapies (Thatcher et al., 2015; Watanabe et al., 2019), along with several VEGF and VEGF receptor inhibitors. Despite not showing the same striking levels of response as targeted treatment for driver mutations, some of these drugs did extend overall survival in patients with lung cancer in phase three trials (Sandler et al., 2006; Reck et al., 2009; Reck et al., 2014).

Screenings of medicinal plants used as anticancer drugs have provided modern medicine with effective cytotoxic pharmaceuticals (Balunas and Kinghorn, 2005). The diversity of the biosynthetic pathways in plants has provided a variety of lead structures that have been used in drug development (Tejesvi et al., 2007). Thus, in the past decade, targeting natural compounds has been particularly successful in the field of anticancer drug research (Ashrafizadeh et al., 2020a; Ashrafizadeh et al., 2020b; Mirzaei et al., 2021; Abadi et al., 2022). Due to the increase in this public health problem, African people have chosen to take alternative medicine from traditional healers so that they can fight these diseases, because they cannot afford Western medicine (Abdullahi, 2011). In Africa, most medicines are from natural products, particularly, plants, where plant barks; leaves, or roots are dissolved in boiled water and taken orally or applied on wounds (Ozioma and Chinwe, 2019). This shows the importance of natural products in African countries. Even when looking at pharmaceuticals, worldwide, most medicines are derived from plants (Mbele et al., 2017).

It is now necessary to study molecular mechanisms of medicinal plants and their bioactive compounds (Lee et al., 2011). The lack of selective diagnostic biomarkers and effective therapeutic drugs has made non-small-cell lung cancer (NSCLC) one of the deadliest diseases. Despite a great emphasis on understanding genetic defects in NSCLC, its molecular pathogenesis remains unclear (Shi et al., 2012).

In recent years, it has been possible to develop agents that target specific molecular pathways in malignant cells as a result of a better understanding of the mechanisms that drive malignancy in non-small cell lung cancer (Zhou et al., 2020). The key driving forces behind cancer (CSC) stem cells, including PI3K/AKT/mTOR and JAK/STAT3, had been shown to be highly regulated in high-CSC cancers, and clinical trials are being conducted to identify small molecules that target these pathways (Losuwannarak et al., 2019). Despite being a small percentage of the total cancer cell population in lung adenocarcinomas, CSCs from patients contributed to tumorigenesis and relapse (Gomez-Casal et al., 2013).

JAK/STAT pathway in mammals is the principal pathway signalling cytokines, growth factors, cell migration, and apoptosis. Activated JAK/STAT pathway promotes cell proliferation and differentiation, as well as migration and apoptosis (Rawlings et al., 2004). This pathway involves two protein families, the JAKs and the STATs, which are activated after tyrosine receptors are phosphorylated. Upon activation of JAKs, phosphorylation of the tyrosine motifs in the cytoplasmic tail of the receptor enables STAT binding (Bolli et al., 2003). In response to growth factors and cytokines, the transcription factor STAT3 is phosphorylated at tyrosine 705 (Tyr705), and it functions in a wide range of cellular functions such as cell proliferation, survival, inflammation, metabolism, and immunity. STAT3 is constitutively activated in cancer cells, unlike normal cells where it is strictly regulated (Khan et al., 2015; Huang et al., 2016). STAT3 functions as a hub for many oncogenic pathways, so inhibiting STAT3 could lead to the inhibition of several upstream signalling pathways at the same time (Lee et al., 2019). Despite the existence of numerous STAT3 inhibitors, none of them have achieved FDA approval for use in clinical trials for lung cancer, indicating that inhibiting STAT3 alone may not be sufficient to eradicate cancer cells (Huang et al., 2016). Therefore, it is imperative to identify novel therapeutic agents that can suppress STAT3 signalling and trigger apoptosis simultaneously through different mechanisms (Khan et al., 2020). In 50%–70% of patients with NSCLC, the PI3K pathway is active based on the AKT phosphorylation (Tsurutani et al., 2006).

In lung cancer, abnormal activation of PI3K/AKT signalling is a common occurrence. The tumour suppressor phosphatase, (PTEN), was the first to demonstrate the importance of PI3K/Akt pathway in cancer (Li et al., 1997). PTEN dephosphorylates the 3′-position on the inositol ring, which results in the elimination of the second messenger, PIP3, which then terminates signaling through this pathway (Li et al., 1997; Maehama and Dixon, 1998). This pathway’s activation was clearly established as one of the key pathways underpinning tumorigenesis where mutations in the *PIK3CA* gene, encoding the p110α PI3K catalytic subunit (Samuels et al., 2004), which resulted in constitutive activation of this pathway (Samuels et al., 2005). In non-small lung cancer cells, the overexpression of p110α was significantly associated with AKT activation. A study using a lung cancer cell line, NCI-H460, with a PI3K allele (NCI-H460) had their p110α expression manipulated, both *in vitro* and *in vivo* successfully, consequently, decreasing proliferation of non-small lung cancer cells (Scrima et al., 2012). Through the inhibition of PI3K/Akt/mTOR signalling pathways and activation of JNK and p38 MAPK signalling pathways, platycodin-D induced autophagy in NCI-H460 and A549 cells (Zhao et al., 2015).

Previous efforts to determine whether or not the transcription factor and tumour suppressor protein *p53* is required for DNA damage-induced apoptosis in human cancer cells produced contradictory conclusions (Jaiswal et al., 2020). Some studies concluded that *p53* maintains pluripotency, and then promotes differentiation in response to DNA damage or developmental signals. Some concluded that pluripotent embryonic stem cells require *p53* for apoptosis (Qin et al., 2007; He et al., 2016a); others concluded they do not. For example, in one study, doxorubicin (Adriamycin) did not induce apoptosis in *p53*−/− pluripotent embryonic stem cells, whereas in another, Adriamycin induced apoptosis in >90% of *p53*−/− pluripotent embryonic stem cells (Aladjem et al., 1998; Prost et al., 1998). These reports unequivocally demonstrated that the multiple roles of *p53* in cell cycle regulation and apoptosis are first acquired during pluripotent stem cell differentiation.

*P53* also plays a major role in the response to many anticancer therapeutics, particularly those that cause DNA damage (Aubrey et al., 2018). The *p53* expression and function might be associated with the suppressive effect of most medicinal plants on cell growth and proliferation of cancer cells. *P53* knockout mice were completely resistant to apoptosis induced by γ-radiation and treatment with chemotherapeutic drugs that induced DNA damage (e.g., etoposide, cyclophosphamide, cisplatin) (Strasser et al., 1995; Aubrey et al., 2018). *P53* is the most commonly mutated tumour-suppressor gene in human cancers (Bykov et al., 2018; Sabapathy and Lane, 2018). Over 50% of human cancers overexpress mutant forms of *p53*. A growing number of studies suggests that the nature of a *p53* mutation in a cell can impact upon cellular properties, clinical responses to therapy and prognosis of a tumour (Canale et al., 2017; Labbé et al., 2017).

It remains unclear how *p53* handles the different signals to decide the appropriate cell fate in response to stress, and how these responses are implicated in tumorigenesis and cancer progression. They can also be associated with response to treatment, depending on the cell context. The human *p53* gene contains two promoters, multiple exons and polyadenylation sites, thus, it is transcribed into multiple variants (Bourdon et al., 2005). The analysis of the expression of *p53* isoforms and *p53* mutation status may help to define different subtypes within a particular cancer type, which would have different responses to treatment. Thus, the understanding of the regulation of *p53* transcripts expression and their biological activities in relation to the cellular context would constitute an important step toward the improvement of the diagnostic, prognostic, and predictive values of *p53* in cancer treatment (Bourdon et al., 2014).

Depending on the nature of the genetic alteration, *p53* induces either cell growth arrest or apoptosis (Jovanović et al., 2019; Mijit et al., 2020). Different *p53* isoforms play an important role in regulating cell fate in response to different stimuli in normal cells by differentially regulating gene expression. In cancer cells, abnormal expression of *p53* isoforms contributes actively to cancer formation and progression, regardless of *p53* mutation status. However, clinical studies failed to establish *p53* mutation status as a clear predictive or prognostic factor of cancer progression and treatment. Kosaka et al. (2009) reported that *p53* gene mutations were not independently associated with the prognosis for patients with surgically treated lung adenocarcinoma. The recent discovery of p53 isoforms that can differentially regulate cell cycle arrest and apoptosis suggests that their expression, rather than p53 mutations, could be more relevant in cancer, and can be targeted as prognostic biomarkers. Nevertheless, uncovering of *p53* isoforms has opened new perspectives in the cancer research field. This study was aimed at analysing two *p53* splice variants during the potential anticancer activities of *Drimia calcarata* methanol and water extracts against different human lung cancer cell lines of varying *p53* mutation status, and these included *p53*-mutant H1573 and *p53*-mutant H1437 and *p53*-wild type A549 cells.

## 2 Methods and Materials

### 2.1 Plant Extracts Preparation

*Drimia calcarata* bulbs were harvested from the Phalakwane village, Ga-Mphahlele in the Limpopo province, South Africa. The bulbs were air-dried and pulverized into powder using a laboratory grinder. Powdered plant material was thoroughly extracted using methanol and water (1:10 w/v), following a protocol previously reported by (Eloff, 1998). Following extraction, the samples were air-dried and the dried plant extracts were reconstituted in acetone for phytochemical analysis and dimethylsulphoxide (DMSO) (Sarchem, RSA) for all the cell culture-based assays.

### 2.3 Cell Culture and Cell Viability

Cell culture and MTT assay were performed following a method that has been previously reported (Laka et al., 2019). Different media were used and these included the Roswell Park Memorial Institute 1,640 (RPMI-1640) for A549, H1573 and H1437, Eagle’s Minimum Essential Medium (EMEM) (Hyclone, United States) for the MRC-5 while Dulbecco’s Modified Eagle Medium (DMEM) (Hyclone, United States) was used for the HEK-293 cells. All media contained Foetal Bovine Serum (10%) (Hyclone, United States) and a mixture of penicillin-streptomycins (1%) (Biowest, United States). The cell lines were kept in a 5% CO_2_ chamber at 37°C. The cytotoxicity of the different plant extracts was assessed using the 3-(4,5-dimethylthiazol-2-yl)-2,5-diphenyltetrazolium bromide (MTT) assay. Three cell lines; A549 (lung carcinoma cells, CCL-185™), H1573 (lung adenocarcinoma, CRL-5802™), H1437 (lung adenocarcinoma, CRL-5872™) were used. Initially, the cells were seeded at a concentration of 1 × 10^3^ cells/well in 96-well culture plate overnight and treated with different concentrations of each extract, solvent controls (0.25% DMSO, 0.25% H_2_O) and positive control (50 µM curcumin). Following treatment, the cells in each well were subjected to 5 mg/ml MTT reagent (10 µl) (ThermoFischer Scientific, United States) and incubate in the CO_2_ incubator for 3 h. After incubation, the formed crystals were dissolved by adding 100 µl DMSO and placed in the dark for an hour at 25°C. Thereafter, the absorbance readings were measured using microtitre plate reader (Promega, United States) at 560 nm. The cell viability was analysed using the formula (Sung et al., 2021) as previously reported (Makgoo et al., 2019).

$$Cell\;viability\;\left(\%\right)=\frac{sample\;absorbance\;\left(560\;nm\right)}{\text{untreated absorbance }\left(560\text{ nm}\right)}\;X\;100$$ (1)

### 2.4 Morphological Examination (Fluorescence Microscopy Imaging)

The effect of the extracts on the morphological features of lung cancer A549, H1573 and H1437 cells was determined using fluorescence microscopy as describe previously (Ezhilarasan et al., 2017). Cells were seeded at an initial concentration of 1 × 10^5^ in 24 well culture plates and exposed to solvent controls (0.25% DMSO, 0.25% H_2_O), positive control (50 µM curcumin) and IC_50s_ of ME (62.50 μg/ml) and WE (125 μg/ml), for 24 h. Cells were fixed for 10 min with 3.7% paraformaldehyde, followed by staining with AO/EB (1 μg/ml). After washing with one X PBS, morphological changes were observed under the Eclipse Ti-U fluorescence microscope (Nikon Instruments Inc., United States) and captured using DSRI-1 camera (Nikon Instruments Inc., United States).

### 2.5 Annexin V and Dead Cell Assay

The apoptosis analysis was carried out using the protocol previously described by Acikgoz et al. (2021). Apoptosis was induced by seeding the cells at 1 × 10^5^ cells/well, overnight, after which incubate them in the presence of *D. calcarata* extracts using the IC_50_ values for 24 h. The Muse^®^ Annexin V and Dead Cell reagent (Merck-Millipore, Germany) was used to stain the cells. The cells were then placed in the dark for 20 min at room temperature. The Muse^®^ Cell Analyser (Merck-Millipore, Germany) was used to analyse the samples.

### 2.6 Cell Cycle Analysis Assay

The cell cycle analysis was carried out using the protocol previously described by Kwak et al. (2021). Cell culture flasks (25 cm) were used to grow cells overnight. Following culturing, cells were treated with solvent controls, positive control and IC_50_s of *D. calcarata* extracts for 24 h. The cells were pelleted by centrifugation and fixed for 3 h in 70% ethanol at −20°C. After fixation, the cells were stained with the Muse^®^ Cell Cycle Reagent (Merck-Millipore, Germany) and placed in the dark for 30 min. The Muse^®^ Cell Analyser was used to analyse the samples.

### 2.7 Reverse Transcription-Polymerase Chain Reaction Components

ZR^®^ RNA MiniPrep Kit (Zymo Research, United States) was used for total RNA extraction and manufacturer’s instructions were followed. The complementary deoxyribonucleic acid (cDNA) was synthesised using a Promega AMV II Reverse Transcription System (United States). The EmeraldAmp^®^ GT PCR Kit (Takara Bio, United States) was employed for the amplification of apoptosis-related genes, cell cycle-related genes and STAT genes using the primer sets (Table 1). Amplification was done using T100^™^ Thermal Cycler (BioRad, United States). The PCR products were mixed with the novel juice (Genedirex, Taiwan). Samples were visualised using 2% agarose gels, which were viewed using D-DiGit Gel Scanner (LICOR, United States). The band densities from three independent experiments were measured and analysed using ImageJ software [National Institutes of Health (NIH), United States].

**TABLE 1:** The primer sequences of apoptosis related genes, cell cycle related genes and STAT genes.

Gene	Forward primers	Reverse primer	Accession number
*Bcl-2*	5′-GCA​CCG​GGC​ATC​TTC​TCC​TC-3′	5′-CCG​AGA​TGT​CCA​GCC​AGC​TG-3′	NM_000,657.3
*p53*	5′-GTT​GCC​CAG​GCT​GGA​GTG​GAG-3′	5′-GGC​TGA​GAC​AGG​TGG​ATC​GC-3′	NM_000,546.6
*Bax*	5′-GGG​TGG​TTG​GGT​GAG​ACT​C-3′	5′-AGA​CAC​GTA​AGG​AAA​CGC​ATT​A-3′	NM_001,291,429.2
*STAT1*	5′-GCC​CCG​ATG​GTC​TCA​TTC​CG-3′	5′-GTC​CTT​CAA​CAG​GGC​ACG​CT-3′	NM_001,384,880.1
*STAT3*	5′-TGC​CTG​CGG​CAT​CCT​TCT​GC-3′	5′-ACA​GGC​GTG​AGC​CAC​CAT​GC-3′	NM_139,276.3
*STAT5B*	5′-GGA​TGG​GTG​CAT​CGG​GGA​AG-3′	5′-TCT​CAG​AGG​CAG​GTG​CTG​GT-3′	NM_012,448.4
*STAT5A*	5′-AGA​AGC​ACC​ACA​AGC​CCC​AC-3′	5′-GTG​TTT​CCT​GAC​CGC​CCC​AA-3′	NM_001,288,718.2
*CLA1*	5′-GCA​CTG​CAG​CAA​CCC​CAA​GAG-3′	5′-GAG​CTG​CAG​TTT​CCC​TCT​CAG-3′	NM_003,914.4
*CLB1*	5′-GTG​CCA​GTG​CCA​GTG​TCT​GAG-3′	5′-TCA​TGT​TTC​CAG​TGC​TTC​CCG-3′	NM_001,354,844.2
*p21*	5′-GAC​CTG​TCA​CTG​TCT​TGT​AC-3′	5′-GGT​AGA​AAT​CTG​TCA​TGC​TGG-3′	NM_000,389.5
*CDC2*	5′-GGT​TCC​TAG​TAC​TGC​AAT​TCG-3′	5′-TTT​GCC​AGA​AAT​TCG​TTT​GG-3′	NM_033,379.5

### 2.8 Fractionation

The bulb methanol extract and water extract (2 g) were chromatographed on a glass column (30 × 2 cm^2^) packed with silica gel (60 g) dissolved in 50:50 acetone and hexane. Elution was carried out using 50:50 (Acetone: Hexane), 100% Acetone, 50:50 (Acetone: Methanol) for the methanol extract and 50:50 (Acetone: Hexane), 100% Acetone, 50:50 (Acetone: Methanol) and 7:1:2 (Butanol: Acetic acid: Distilled water). The solvents were removed under a stream of cold air at room temperature. Once the solvents were evaporated, the samples were dissolved in DMSO/water for cell culture experiments and Liquid Chromatography-Mass Spectrometry (LC-MS) Analysis.

### 2.9 Liquid Chromatography-Mass Spectrometry Analysis

Fraction samples (1 mg/ml) were prepared. Fifty percent methanol in water containing 1% formic acid was used to prepare the samples. The LC-MS analysis was done as previously described (Stander et al., 2017). Calibration, calculation and the rest of the settings were done using polyalanine as previously reported (Rautenbach et al., 2017).

### 2.10 Statistical Significance

GraphPad Prism Version 6.0 was employed for graphical data analysis presented as mean ± standard error of mean (SEM). The one-way ANOVA Tukey Kramer Multiple Comparison Test was used to verify the statistical significance and the asterisks (*) (**) and (***) were used to indicate *p* ≤ 0.05, *p* ≤ 0.01 and *p* ≤ 0.001, respectively.

## 3 Results

### 3.1 *In vitro* Inhibition of Lung Cancer Cells Growth by *D. calcarata* Bulb Extracts

**FIGURE 1:**
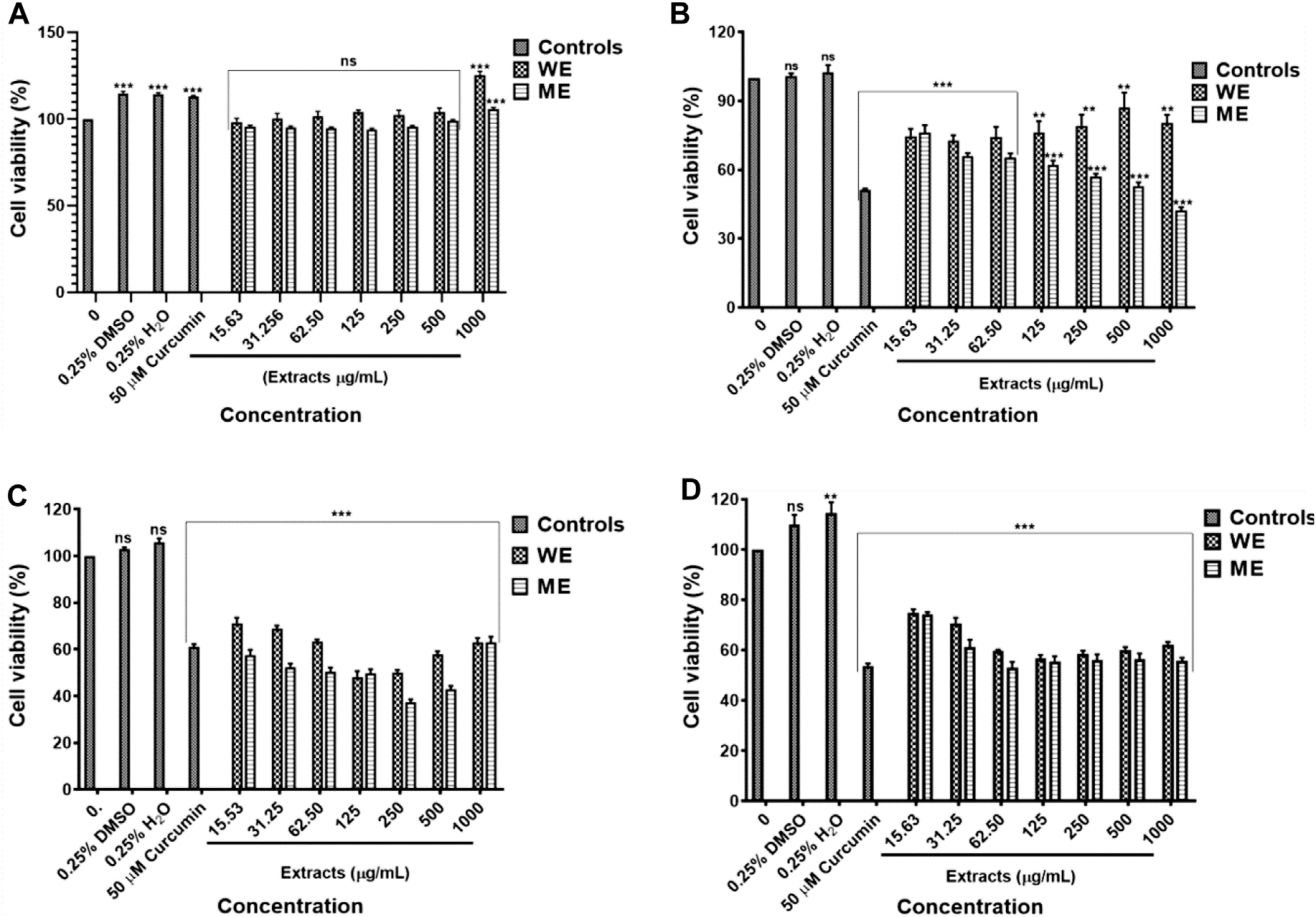
Cytotoxic effect of *D. calcarata* extracts on noncancerous lung MRC-5 fibroblasts **(A)**, lung cancer cells, *p53*-wild type A549 **(B)**, *p53*-mutated H1573 **(C)** and *p53*-mutated H1437 **(D)**. No significant difference on the cell viability was observed after treatment of MRC-5. The water extract (WE) significantly (**p ˂ 0.01) decreased the viability of A549 cells. Both WE and ME significantly (***p < 0.001) reduced the cell viability of H1573 and H1437 cells. Cytotoxicity of selected concentrations of **(D)** calcarata extract using Muse^®^ Count and Viability assay.

The *D. calcarata* extracts showed no significant activity against the normal lung cells MRC-5 (Figure 1A; Supplementary Table S1). The water extract (WE) showed the lowest cytotoxicity activity with no 50% inhibitory concentration (IC_50_) value while methanol extract (ME) had the highest activity with IC_50_ of 500 μg/ml (52.948 ± 1.569) against the *p53*-wild type A549 cells (Figure 1B; Supplementary Table S2). Both extracts showed the highest activity against the *p53*-mutant H1573 (Figure 1C; Supplementary Table S3) and *p53*-mutant H1437 (Figure 1D; Supplementary Table S4) adenocarcinoma lung cancer cells, which showed IC_50_ values of 125 μg/ml ME (49.000 ± 1.807) and WE (47.667 ± 2.348) for H1573 and 62.50 μg/ml ME (52.667 ± 2.108) and 125 μg/ml WE (56.167 ± 1.470) for H1437 cells. The *p53*-wild type A549 looks less sensitive towards the two extracts as compared to *p53*-mutant cell lines, H1573 and H1437.

**FIGURE 2:**
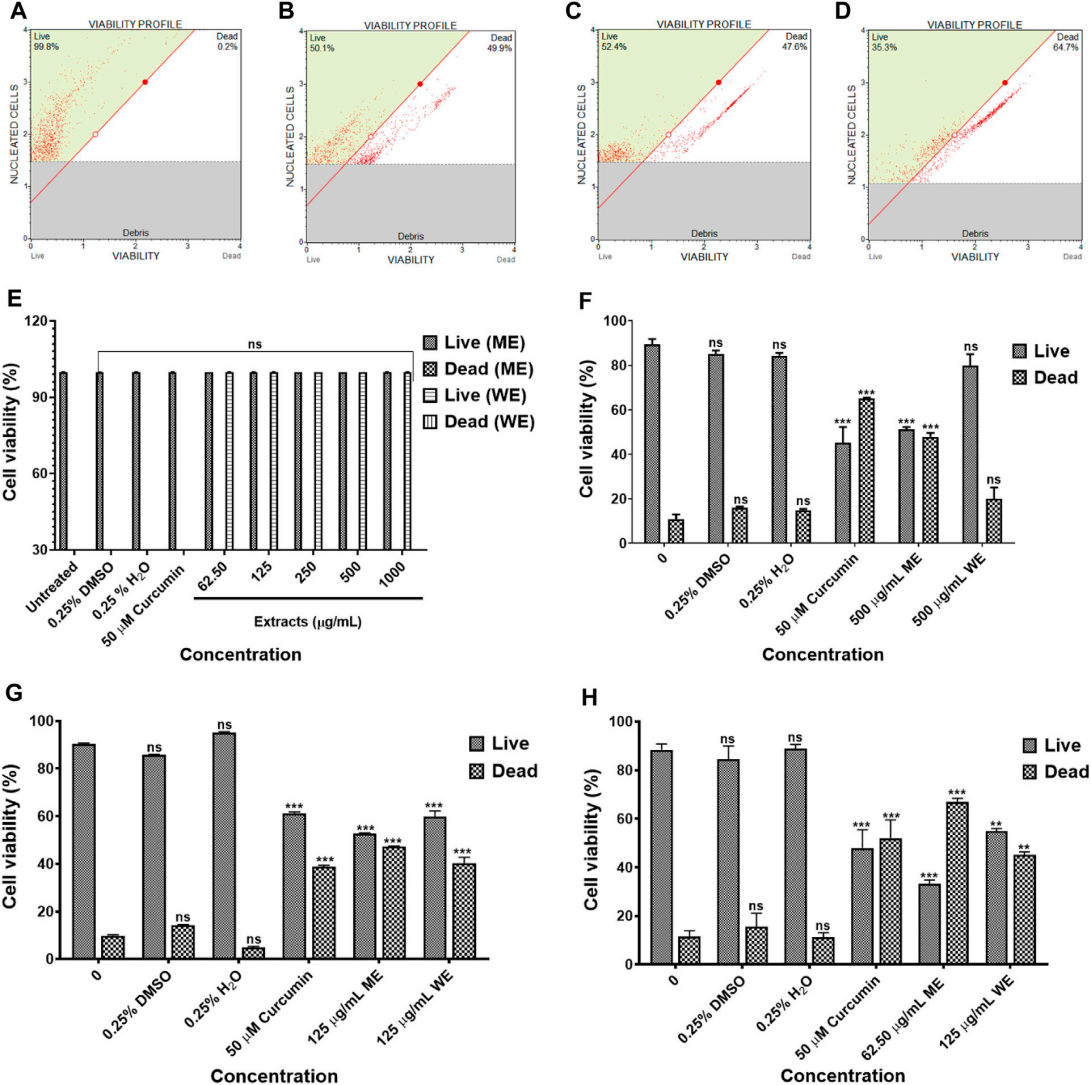
The cytotoxicity data of selected *D. calcarata* concentrations on MRC-5 **(A,E)**, *p53*-wild type A549 **(B,F)**, *p53*-mutant H1573 **(C,G)** and *p53*-mutant H1437 **(D,H)** cells. The difference was found to be statistically insignificant (ns) after the treatment of A549 with WE. The difference was found to be statistically significant (**p < 0.01 and ***p < 0.001) after treatment of H1573 and H1437 with WE and ME. Comparing with the untreated control.

The IC_50_ values obtained from the MTT assay were used to verify the cell viability reduction by the *D. calcarata* extracts against the normal lung cells and lung cancer cells, using the MUSE Count and Viability Assay. Methanol extract exhibited pronounced cytotoxic effects in all the cell lines tested: *p53*-wild type A549 (Figures 2B,F), *p53*-mutant H1573 (Figures 2C,G) and *p53*-mutant H1437 (Figures 2D,H) (51.550 ± 1.267, 52.733 ± 0.203, and 32.295 ± 1.661, respectively). Water extract showed no significant effect on the A549 cell viability (80.000 ± 5.056), whereas it showed best activity against the H1573 cells (59.700 ± 2.488) and H1437 cells (54.824 ± 1.176). The cytotoxic results showing data from three independent experiments are summarised in Supplementary Tables S5–S8.

### 3.2 Acridine Orange/Ethidium Bromide Staining Showed Apoptosis Induction

**FIGURE 3:**
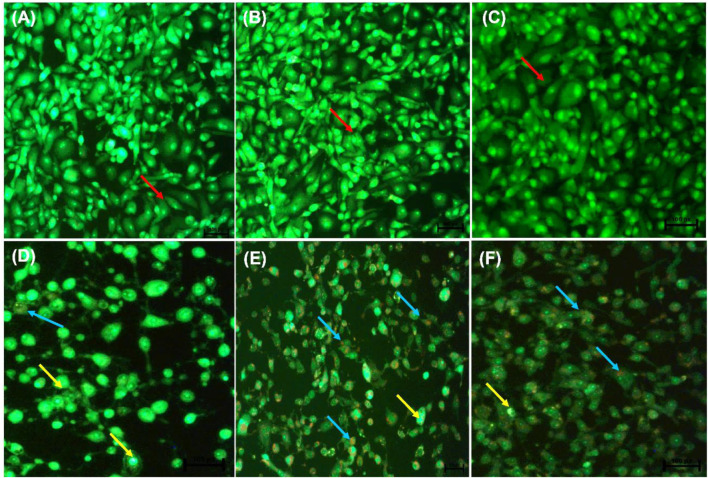
Nuclear morphology of *p53*-wild type A549 cells after AO/EB staining. *D. calcarata* extracts induced typical apoptotic changes in cultured A549 cells: **(A)** untreated cells, **(B)** 0.25% DMSO, **(C)** 0.25% H_2_O, **(D)**-50 µM Curcumin, **(E)**-500 μg/ml ME, **(F)**-500 WE. The samples were analysed using an Eclipse Ti-U fluorescence microscope (Nickon Instruments Inc. United States) and images were captured at ×20 magnification. The red arrows indicate intact nuclei, while yellow arrows show cells undergoing early apoptosis and blue arrows point to cells displaying late apoptosis.

Following AO/EB staining (Figures 3A–F), untreated control cells (A) and 0.25% DMSO treated cells (B) and 0.25% H_2_O (C) displayed intact nuclei (red arrows). *P53*-wild type lung cancer A549 cells that were exposed to curcumin (D) and *D. calcarata* extracts (E-F) displayed a large number of cells undergoing early apoptosis, which fluoresced green/yellow (yellow arrows) and cells showing late stage of apoptosis, which showed uneven orange fluorescence at their periphery (blue arrows). The number of cells slighly decreased comparing with the untreated control.

**FIGURE 4:**
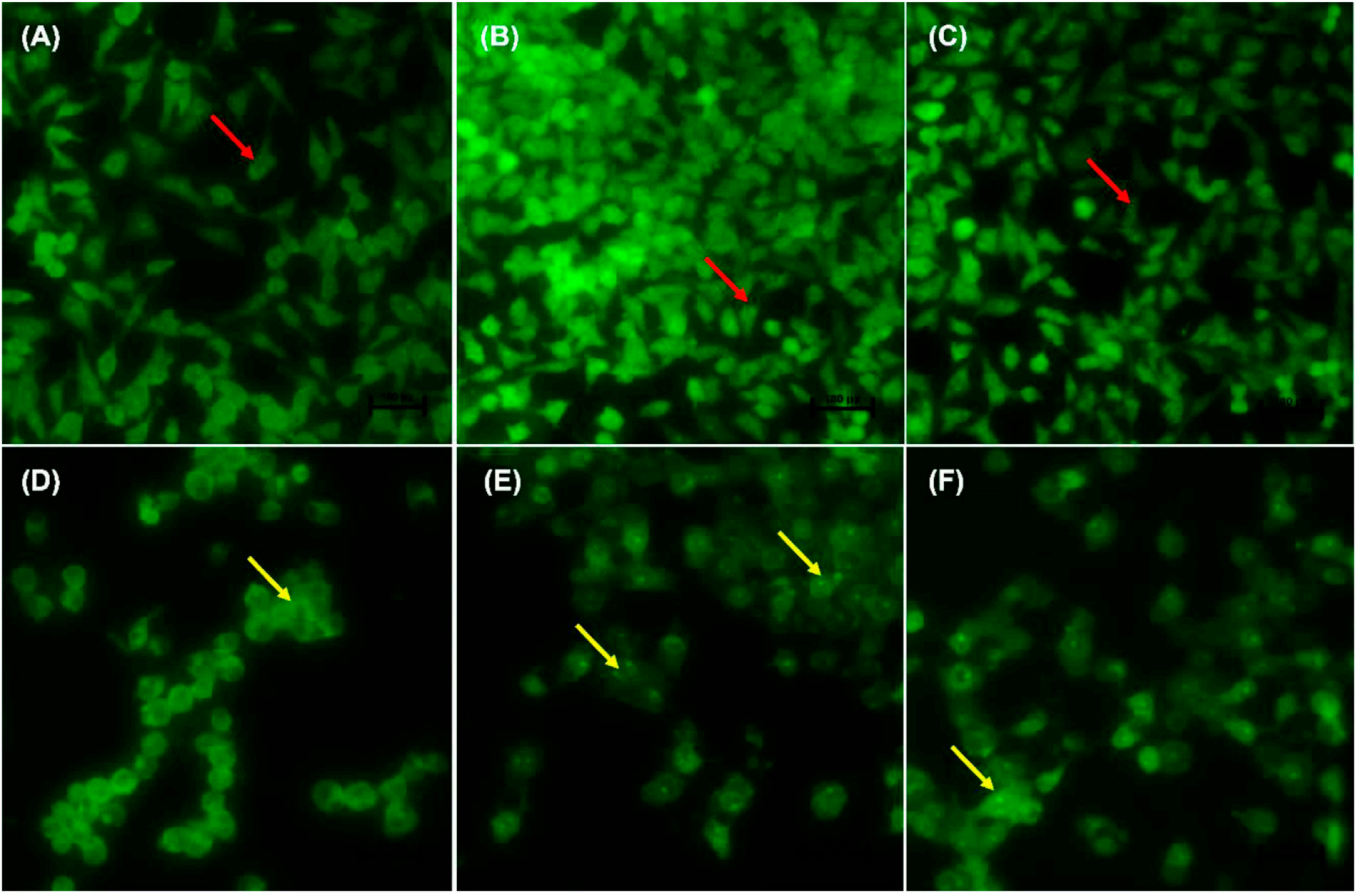
Nuclear morphology of *p53*-mutant H1573 cells after AO/EB staining. *D. calcarata* extracts induce morphological changes in cultured cells: **(A)**—untreated cells, **(B)** 0.25% DMSO, **(C)** 0.25% H_2_O, **(D)** 50 µM Curcumin, **(E)** 125 μg/ml ME, **(F)** 125 μg/ml WE. The samples were analysed using an Eclipse Ti-U fluorescence microscope (Nickon Instruments Inc. United States) and images were captured at ×20 magnification. The red arrows indicate intact nuclei, while yellow arrows depict early apoptotic cells.

**FIGURE 5:**
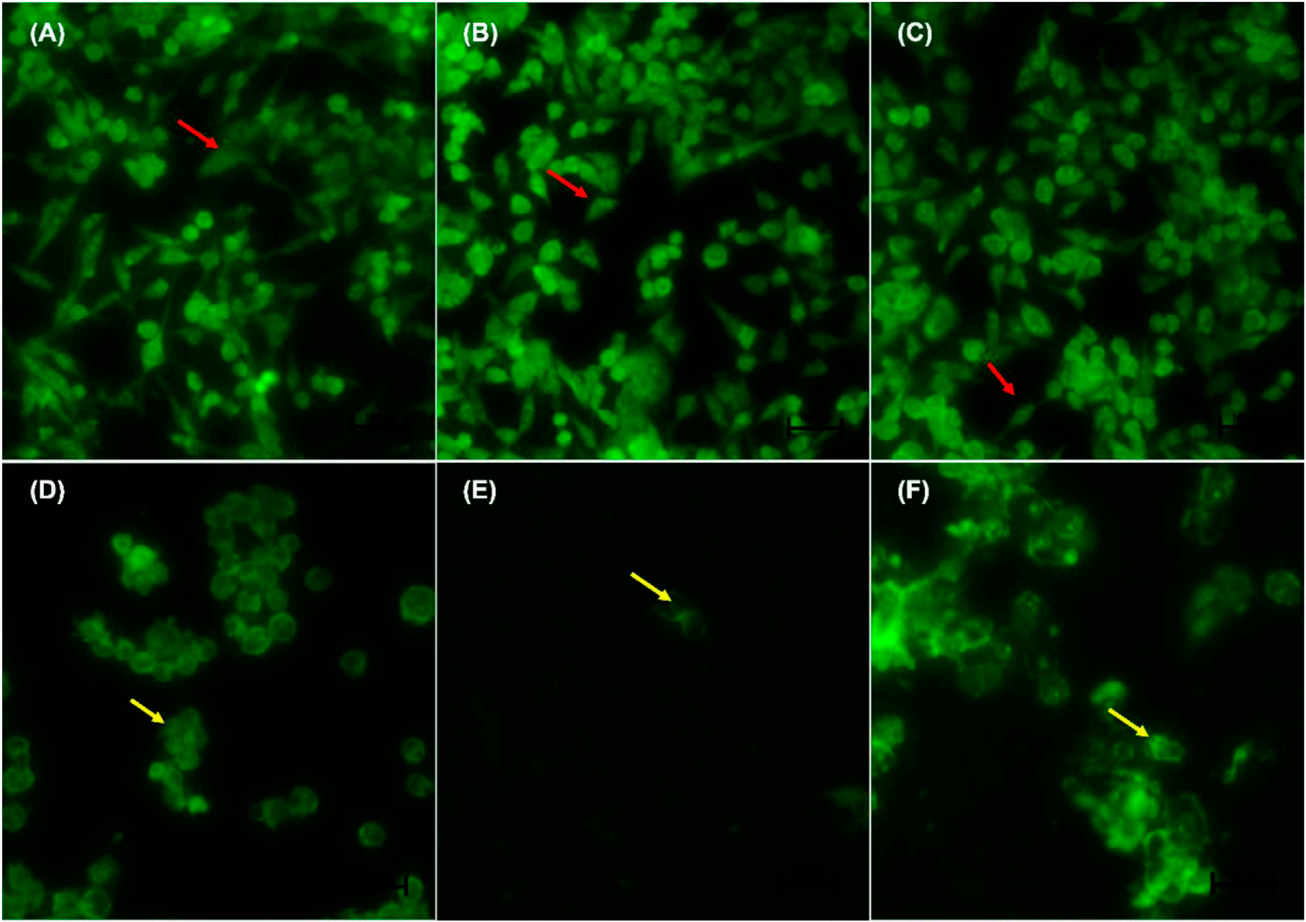
Nuclear morphology of *p53*-mutant H1437 cells after AO/EB staining. *D. calcarata* extracts induced typical apoptotic changes in cultured cells: **(A)** untreated cells, **(B)**−0.25% DMSO, **(C)** 0.25% H_2_O, **(D)**-50 µM Curcumin, **(E)**—62.50 μg/ml ME, **(F)**—125 μg/ml WE. The samples were analysed using an Eclipse Ti-U fluorescence microscope (Nickon Instruments Inc. United States) and images were captured at ×20 magnification. The red arrows indicate intact nuclei, while yellow arrows show cells going through early apoptosis.

Figures 4A–F indicate the morphological changes of *p53*-mutant H1573 cancer cells. Untreated control cells (A) and 0.25% DMSO (B) and 0.25% H_2_O-treated (C) cells displayed intact nuclei (red arrows), while lung cancer cells exposed to curcumin (D) and *D. calcarata* extracts (E-F) contained many cells going through early apoptosis, which fluoresced green/yellow (yellow arrows) and reduced cell number. Figures 5A–F indicate the morphological changes of *p53*-mutant H1437 cancer cells. Following AO/EB staining, untreated control cells (A) and 0.25% DMSO (B) and 0.25% H_2_O treated (C) cells displayed intact nuclei (red arrows), while lung cancer cells exposed to curcumin (D) and *D. calcarata* extracts (E-F) contained many cells demonstrating early apoptosis, which fluoresced green/yellow (yellow arrows) and reduced cell numbers.

### 3.3 Apoptosis Analysis Using Annexin V

**FIGURE 6:**
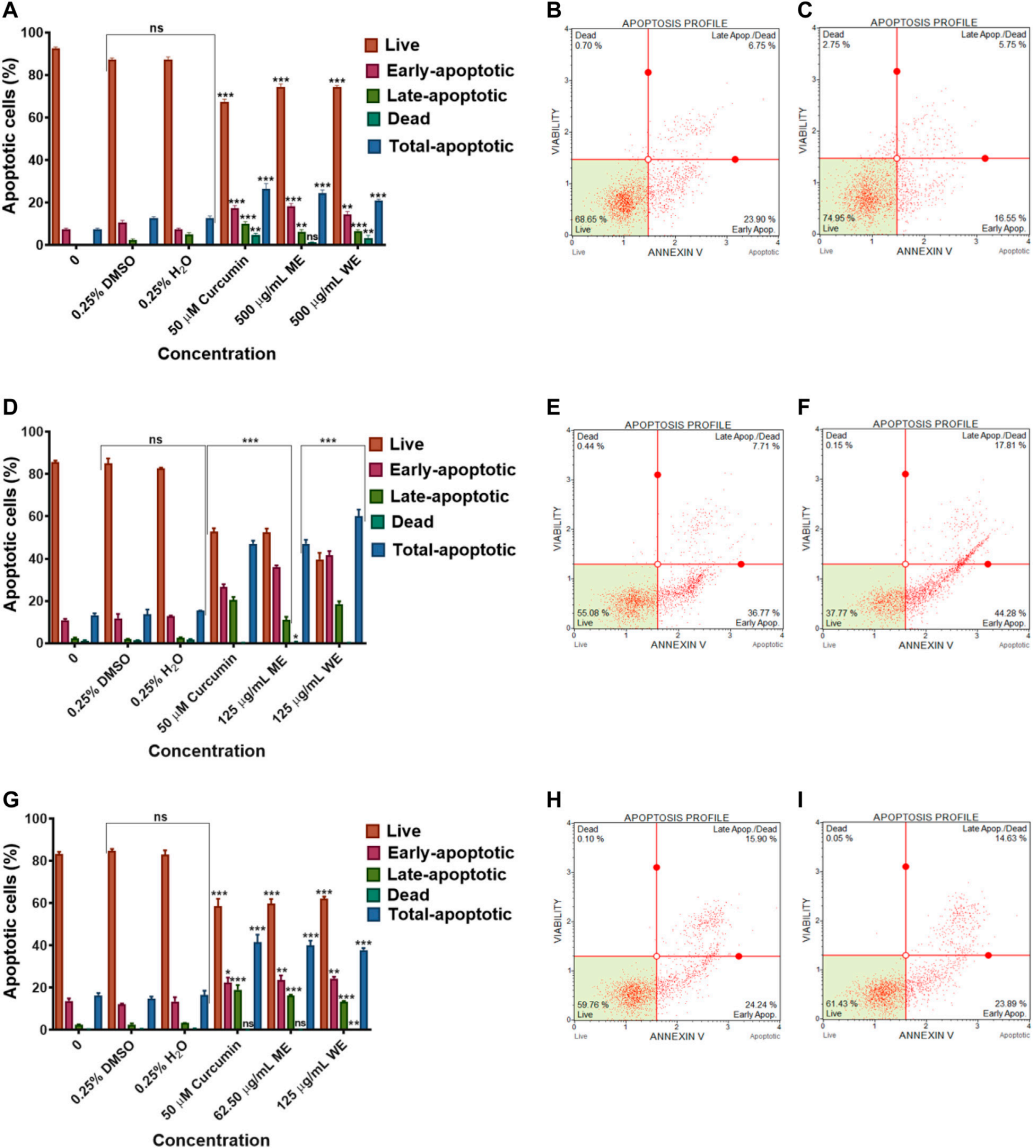
Apoptosis evaluation in *p53*-wild type A549 **(A–C)**, *p53*-mutant H1573 **(D–F)** and *p53*-mutant H1437 **(G–I)** cells after 24 h treatment. In **(A–D)**, solvent controls had no significant (ns) effects on all the lung cancer cell lines. All *D. calcarata* extracts and positive control agent significantly (***p < 0.001) induced apoptosis in lung cancer cells comparing with the untreated control.

To identify the type of cell death induced by the *D. calcarata* extracts, especially, specifically, whether the extracts induced necrosis or apoptosis in lung cancer cells, with the MUSE Annexin V and Cell Dead Kit was used. Figure 6 shows the analysis of live cells, cells at the early stages of apoptosis, those at the late stages of apoptosis and necrotic cells. As showed in Figure 6, the number of live cells in response to different extracts can be used as an index of apoptosis efficiency when compared to control. All extracts indicated minute significant reduction of live cells in A549 (Figures 6A–C) cells and significantly (*p* < 0.001) decreased the live cells of H1573 (52.393 ± 1.83 ME and 39.700 ± 073 WE) (Figures 6D–F; Supplementary Figure S7; Supplementary Table S10) and H1437 (59.270 ± 2.564 ME and 62.114 ± 0.974 WE) (Figures 6G–I; Supplementary Figure S8; Supplementary Table S11) in comparison with the untreated control cells, which showed a non-significant change of live cells. *D. calcarata* ME and WE induced both early and late apoptosis in H1573, H1437 and A549 lung cancer cells. Both extracts induced more early apoptosis in all three cells, with H1573 cells exhibiting more apoptosis than H1437 cells, with A549 demonstrating the least. In addition, both extracts showed a significant increase of dead cells in A549 cells than the untreated control cells; however, the *p53*-wild type A549 cells were less sensitive compared to the other two *p53*-mutant cell lines.

### 3.4 Expression Analysis of Apoptosis-Related Genes

**FIGURE 7:**
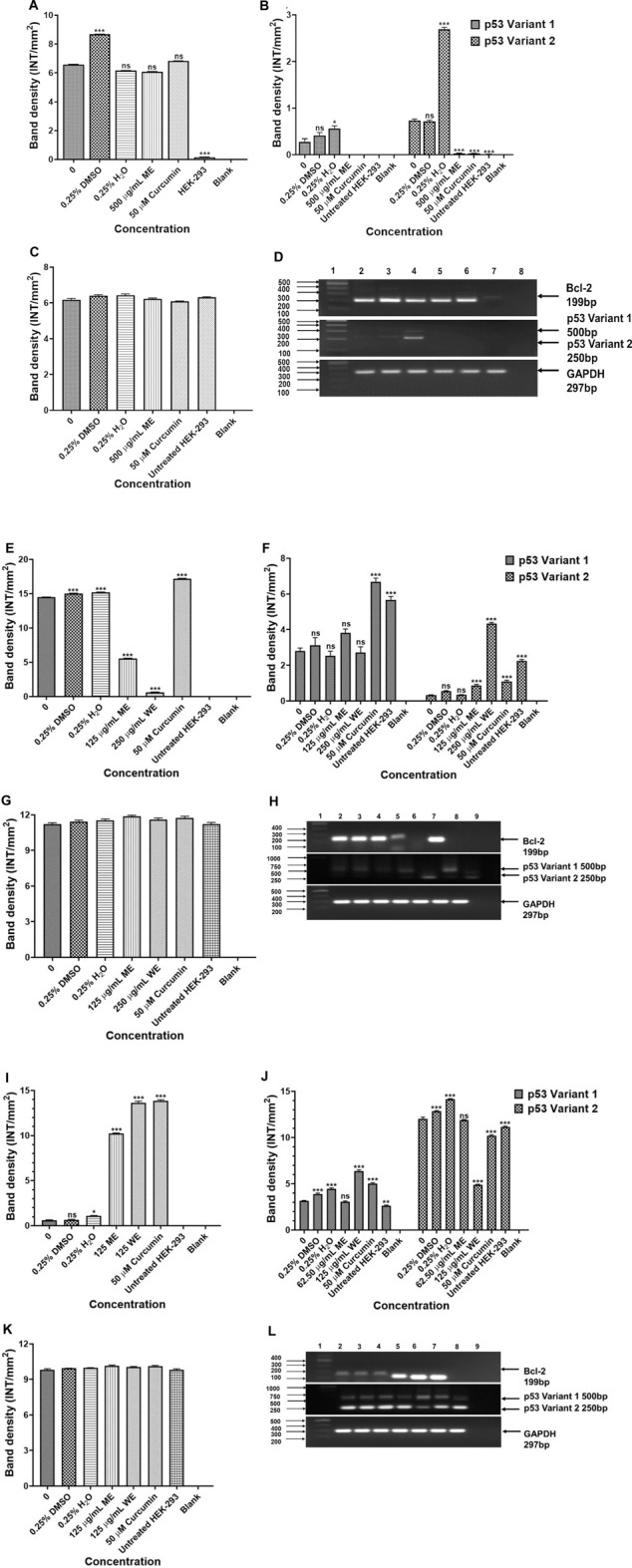
Analysis PCR band intensities analysis of apoptotic genes in *p53*-wild type A549 [**(A)**—*Bcl-2*, **(B)** *p53* variants, **(C)** *GAPDH* and **(D)** PCR gels], *p53*-mutant H1573 [**(E)**-Bcl-2, **(F)**-*p53* variants, **(G)**-*GAPDH* and **(H)**-PCR gels] and *p53*-mutant H1437 [**(I)**-*Bcl-2*, **(J)**-*p53* variants, **(K)**-*GAPDH* and **(L)**-PCR gels] cells. A549 **(D)** Lane 1: 100/1,000 bp Ladder, Lane 2: untreated, Lane 3: 0.25% DMSO, Lane 4: 0.25% H_2_O, Lane 5: 500 μg/ml ME, Lane 6: 50 μM curcumin, Lane 7: HEK-293 cells and Lane 8: blank. H1573 **(H)** Lane 1: 100/1,000 bp DNA Ladder, Lane 2: untreated, Lane 3: 0.25% DMSO, Lane 4: 0.25% H_2_O, Lane 5: 125 μg/ml ME, Lane 6: 125 μg/ml WE, Lane 7: 50 μM curcumin, Lane 8: HEK-293 cells and Lane 9: blank. *GAPDH* was used as a loading control. H1437 **(L)** Lane 1: 100/1,000 bp DNA Ladder, Lane 2: untreated, Lane 3: 0.25% DMSO, Lane 4: 0.25% H_2_O, Lane 5: 62.50 μg/ml ME, Lane 6: 125 μg/ml WE, Lane 7: 50 μM curcumin, Lane 8: HEK-293 cells and Lane 9: blank. *GAPDH* was used as a loading control.

To investigate the mechanism of apoptosis induced by the *D. calcarata* extracts after 24 h incubation of lung cancer cells, RT-PCR was used to evaluate the expression of several apoptosis genes, including *p53*, *Bcl-2* and *Bax* for evaluation of apoptotic cell death mechanism. Results in Figures 7A,B show that ME slightly decreased the antiapoptotic *Bcl-2* and highly decreased the expression of both *p53* variants, 1 and 2 in *p53*-wild type A549 cells. There was no expression of the pro-apoptotic gene, *Bax* in both the untreated and treated *p53*-wild type A549 cells. Following treatment of *p53*-mutant H1573, the *D. calcarata* extracts had no significant effect on the mRNA levels of *p53* variants. Figure 7E shows that there was a significant (*p* < 0.001) decrease in the level of *Bcl-2* expression when compared with the untreated control of A549 cells. The mRNA expression level of *p53* variant one was not affected by ME while, WE and curcumin significantly increased the *p53* variant 1 (*p* < 0.001) in *p53*-mutant H1437 cells (Figure 7F). Both extracts and curcumin significantly (*p* < 0.001) upregulated the mRNA expression of *Bcl-2* in H1437 cells compared with the untreated control (Figure 7I). The methanol extract and curcumin significantly (*p* < 0.05 and *p* < 0.001, respectively) increased *p53* variant 2. On the contrast, WE significantly (*p* < 0.001) decreased the mRNA expression of *p53* variant 2 (Figure 7J). The results are summarised in Supplementary Table S12–S14 showing data from three independent experiments.

### 3.5 Cell Cycle Analysis

**FIGURE 8:**
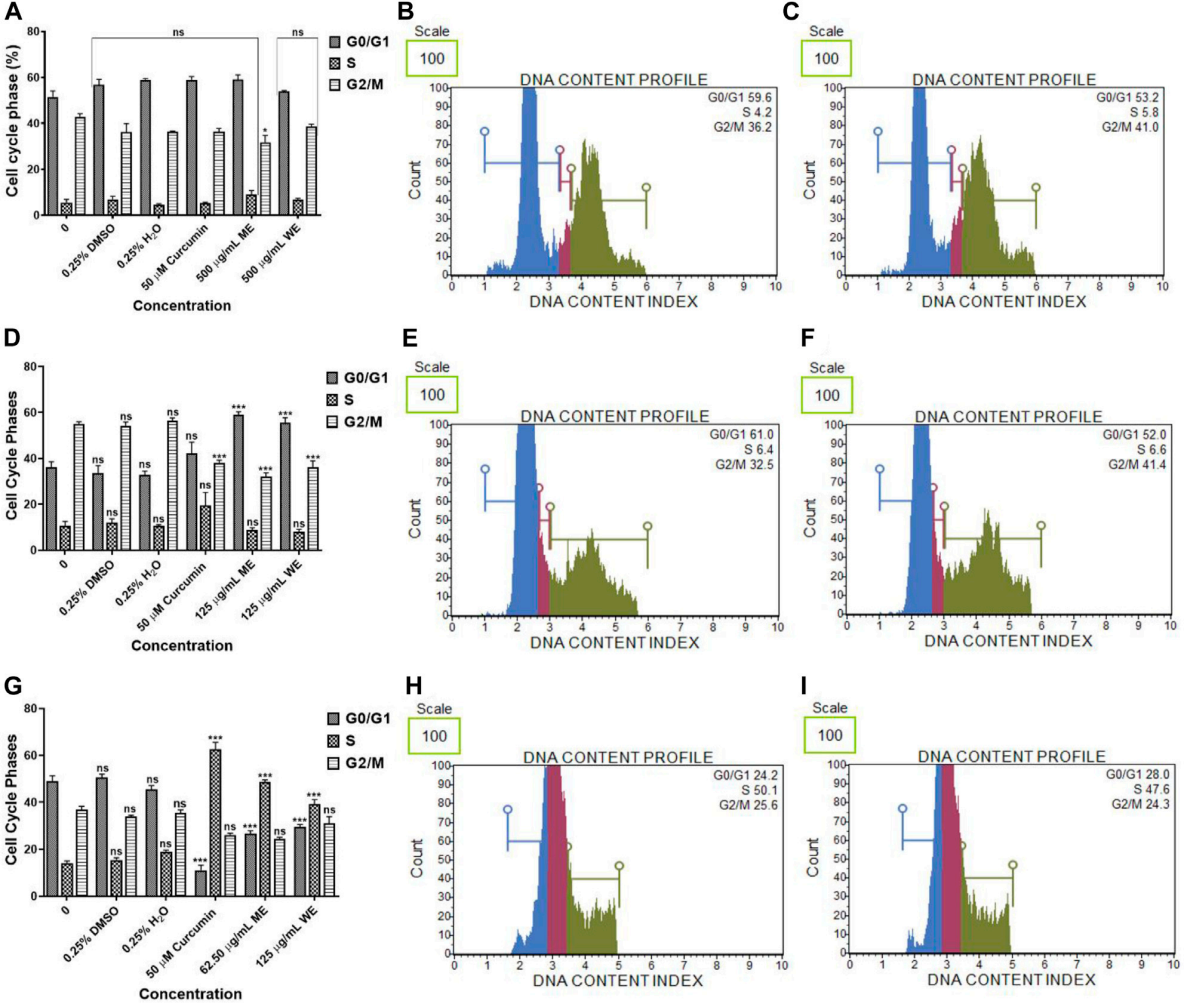
The percentage of *p53*-wild type A549 **(A–C)**, *p53*-mutant H1573 **(D–F)** and *p53*-mutant H1437 (**G–I**) cells in the G0/G1, S and G2/M cell cycle phases after 24 h treatment. *D. calcarata* extracts had no effect on the cell cycle progression of A549, promoted G0/G1 cell cycle arrest of H157 (***p < 0.001) and S-phase cell cycle arrest of H1437 (***p < 0.001) comparing with the untreated control.

The cell cycle was assessed using a PI uptake analysis method using Muse^®^ Cell Cycle Kit. Figures 8A–C, have both extracts had no significant effect on the cell cycle progression of *p53*-wild type A549 cells. There was a significant (*p* < 0.001) increase in the population of *p53*-mutant H1573 cells at the G0/G1 phase. In the untreated control, the mean percentage of G0/G1 cells was 36.100 ± 2.473, while in ME-treated cells, it was 59.100 ± 1.264 and in WE-treated cells, it was 55.667 ± 2.07 and a significant decrease (*p* < 0.001) in the population of H1573 cells at the G2/M phase following treatment with ME and WE as (32.150 ± 1.578 and 36.200 ± 2.728) compared to the untreated control cells (Figures 8D–F; Supplementary Figure S10; Supplementary Table S16). Interestingly, there was a significant increase (*p* < 0.001) in the population of H1437 cells at the S phase, with the untreated control having the least, 14.133 ± 0.899, followed by WE 39.233 ± 1.936 and ME with the highest 48.800 ± 0.723. A significant decrease (*p* < 0.001) in the population of *p53*-mutant H1437 cells at the G0/G1 was observed following treatment with both extracts (26.667 ± 1.235 and 29.567 ± 0.984) (Figures 8G–I; Supplementary Figure S11; Supplementary Table S17).

**FIGURE 9:**
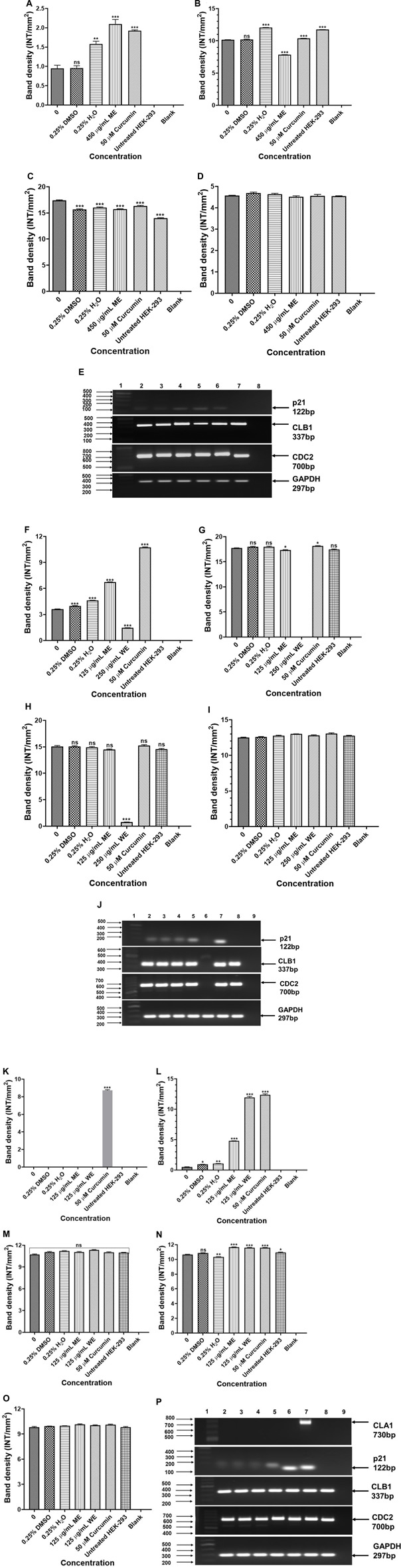
PCR analysis of cell cycle related genes in *p53*-wild type A549 [**(A)**-*p21*, **(B)**-*CLB1*, **(C)**-*CDC2*, **(D)**-*GAPDH* and **(E)**-PCR gels], *p53*-mutant H1573 [**(F)**-*p21*, **(G)**-*CLB1*, **(H)**-*CDC2*, **(I)**-*GAPDH* and **(J)**-PCR gels] and *p53*-mutant H1437 [**(K)**-*p21*, **(L)**-*CLB1*, **(M)**-*CDC2*, **(N)**-*GAPDH* and **(O)**-PCR gels] cells. A549 **(E)** Lane 1: 100/1,000 bp Ladder, Lane 2: untreated, Lane 3: 0.25% DMSO, Lane 4: 0.25% H_2_O, Lane 5: 500 μg/ml ME, Lane 6: 50 μM curcumin, Lane 7: HEK-293 cells and Lane 8: blank. H1573 **(J)** Lane 1: 100/1,000 bp DNA Ladder, Lane 2: untreated, Lane 3: 0.25% DMSO, Lane 4: 0.25% H_2_O, Lane 5: 125 μg/ml ME, Lane 6: 125 μg/ml WE, Lane 7: 50 μM curcumin, Lane 8: HEK-293 cells and Lane 9: blank. *GAPDH* was used as a loading control. H1437 **(P)** Lane 1: 100/1,000 bp DNA Ladder, Lane 2: untreated, Lane 3: 0.25% DMSO, Lane 4: 0.25% H_2_O, Lane 5: 62.50 μg/ml ME, Lane 6: 125 μg/ml WE, Lane 7: 50 μM curcumin, Lane 8: HEK-293 cells and Lane 9: blank. *GAPDH* was used as a loading control.

Expression analysis of Cell cycle-related genes were analysed to determine the effect of *D. calcarata* methanol and water extracts on cell cycle progression of human non-small lung cancer cells. The *p21* was significantly (*p* < 0.001) higher in ME-treated *p53*-wild type A549 cells compared to the untreated control (Figure 9A). The expressions of CLB1 and *CDC2* were significantly upregulated in ME-treated A549 cells compared to the untreated control (Figures 9B,C, respectively). Treatment of *p53*-mutant H1573 with *D. calcarata* extracts upregulated the mRNA expression of *p21*, but in contrast, downregulated *CDC2* (Figures 9F,H). Differential effect of two extracts was observed on the expression of *CLB1*, ME downregulated *CLB1* expression whereas WE increased its expression (Figure 9G). The mRNA expression levels of *p21* (*p* < 0.001), *CLB1* (*p* < 0.05 and *p* < 0.001) and *CDC2* (*p* < 0.001) in the *p53*-mutant H1437 cells were significantly upregulated after treatment with both extracts (Figures 9K–M). Figures 9E,J,P show the PCR band products. The results are summarised in Supplementary Tables S18–S20 showing data from three independent experiments.

### 3.6 Regulation of STATS in Lung Cancer Cells

**FIGURE 10:**
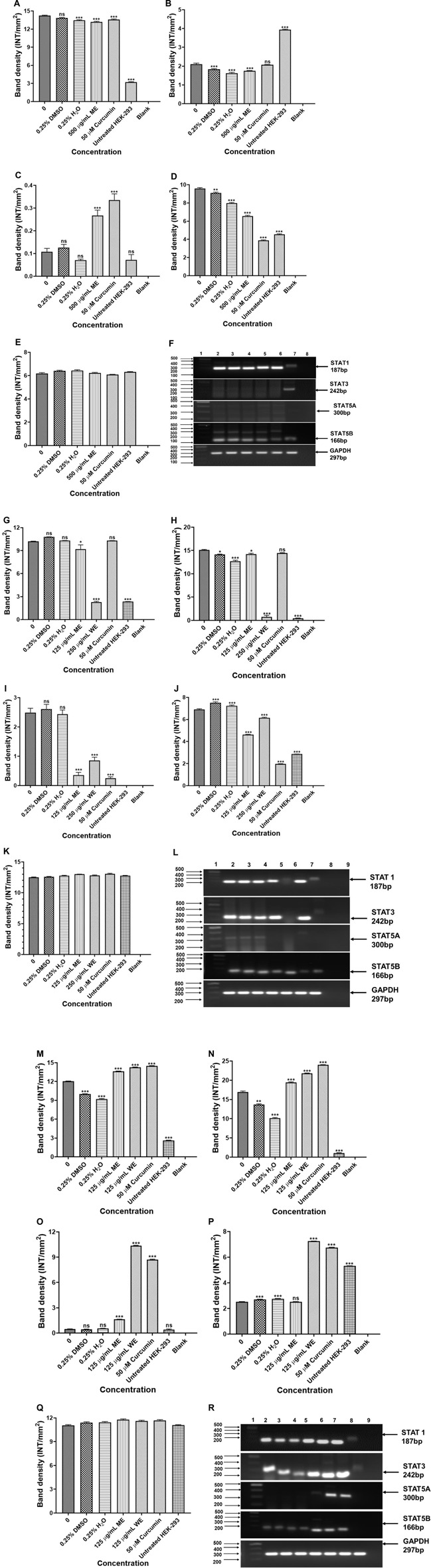
PCR analysis of STATs in *p53*-wild type A549 [**(A)**-*STAT1*, **(B)**-*STAT3*, **(C)**-*STAT5A*, **(D)**-*STAT5B*, **(E)**-*GAPDH* and **(F)**-PCR gels], *p53*-mutant H1573 [**(G)**-*STAT1*, **(H)**-*STAT3*, **(I)**-*STAT5A*, **(J)**-*STAT5B*, **(K)**-*GAPDH* and **(L)**-PCR gels] and *p53*-mutant H1437 [**(M)**-*STAT1*, **(N)**-*STAT3*, **(O)**-*STAT5A*, **(P)**-*STAT5B*, **(Q)**-*GAPDH* and **(R)**-PCR gels] cells. A549 **(F)** Lane 1: 100/1,000 bp Ladder, Lane 2: untreated, Lane 3: 0.25% DMSO, Lane 4: 0.25% H2O, Lane 5: 500 μg/ml ME, Lane 6: 50 μM curcumin, Lane 7: HEK-293 cells and Lane 8: blank. H1573 **(L)** Lane 1: 100/1,000 bp DNA Ladder, Lane 2: untreated, Lane 3: 0.25% DMSO, Lane 4: 0.25% H_2_O, Lane 5: 125 μg/ml ME, Lane 6: 125 μg/ml WE, Lane 7: 50 μM curcumin, Lane 8: HEK-293 cells and Lane 9: blank. *GAPDH* was used as a loading control. H1437 **(R)** Lane 1: 100/1,000 bp DNA Ladder, Lane 2: untreated, Lane 3: 0.25% DMSO, Lane 4: 0.25% H_2_O, Lane 5: 62.50 μg/ml ME, Lane 6: 125 μg/ml WE, Lane 7: 50 μM curcumin, Lane 8: HEK-293 cells and Lane 9: blank. *GAPDH* was used as a loading control.

The potential signalling factors correlated with the change in cell viability and function, after treatment, the mRNA expression of *STAT1*, *STAT3*, *STAT5A* and *STAT5B* were assessed using PCR. The results in Figures 10A–F show that ME downregulated the expression of *STAT1*, *STAT3* and *STAT5A*, while *STAT5B* was upregulated in *p53*-wild type A549 cells (*p* < 0.001). Figures 10G–L, treatment of the *p53*-mutant H1573 cells led to downregulation of *STAT1* (*p* < 0.05 and *p* < 0.001), *STAT3* (*p* < 0.05 and *p* < 0.001), *STAT5A* (*p* < 0.001) and *STAT5B* (*p* < 0.001), respectively. Interestingly, treatment of the H1437 with both extracts resulted in the upregulation of *STAT1*, *STAT3* and *STAT5A* (Figures 10M–O,Q, *p* < 0.001, respectively). However, treatment of the *p53*-mutant H1437 with ME had no significant effect on the expression of *STAT5B*, while ME upregulated *STAT5B* (Figure 10P, *p* < 0.001). The results are summarised in Supplementary Tables S21–S23 showing data from three independent experiments.

### 3.7 Chemical Composition of *D. calcarata* Fractions by Liquid Chromatography Mass Spectrometry

Looking at Table 2, all the fractions contain the compound psoralen (M-H formula mass: 187.09). Water fractions, 3 and 4, share an unknown compound (M-H formula mass: 230.1216), which is not found in the methanol fraction 3. The methanol fractions 1 and 2 and water fractions 1 and 2, only share the compound psoralen (M-H formula mass: 187.09) that is found in all the fractions (Table 3). Furthermore, cardiac glycoside found in abundance in *Drimia* species know as Scillaren A M-H formula mass: 693.6864, Chemical formula: C_36_H_52_O_13_, was present in methanol fraction 3 only. UV chromatograms of the fractions from the LC/MS system are shown in Supplementary Figure S12.

**TABLE 2:** Chemical composition of principal compounds in Water fractions.

Fraction 1.50:50 (Hexane: Acetone)					
**Compound**	**Retention time (min)**	**M-H**	**M-H formula**	**Compound CID**	**References**
Psoralen	19.26	187.0968	C_11_H_6_O_3_	6,199	Wu et al. (2010)
Fraction 2.100% Acetone					
Psoralen	19.26	187.0959	C_11_H_6_O_3_	6,199	Wu et al. (2010)
**Fraction 2. 100% Acetone**					
**Compound**	**Retention time (min)**	**M-H**	**M-H formula**	**Compound CID**	**References**
Baccatin III	21.16	603.2467	C_31_H_38_O_11_	65,366	Lee et al. (2014)
Limonin-17-beta-D-glucoside	24.08	649.2479	C_32_H_42_O_14_	24,820,753	Mandadi et al. (2007); Jayaprakasha et al. (2010); Brito et al. (2014)
Vanillic acid 4-Beta-D-Glucoside	24.38	329.2315	C_14_H_18_O_9_	14,132,336	Gong et al. (2019); Navarro et al. (2019)
**Fraction 3.50:50 (Acetone: Methanol)**					
**Compound**	**Retention time (min)**	**M-H**	**M-H formula**	**Compound CID**	**References**
Eriodictyol 7-O-glucoside	13.24	449.1078	C_21_H_21_O_11_	13,254,473	Areias et al. (2001); Hu et al. (2012)
Psoralen	19.28	187.0952	C_11_H_6_O_3_	6,199	Wu et al. (2010)
Cyanidin 3-O-(6′′ acetyl) glucoside	22.84	491.2101	C_23_H_23_O_12_	—	Brito et al. (2014); Sorrenti et al. (2015)
Scillirubroside/scilliphaeosidin-glucoside	22.84	607.2842	C_28_H_31_O_15_	—	Knittel et al. (2014)
Glucoscilliphaeoside	24.13	707.3246	C_36_H_52_O_14_	—	Kakouri et al. (2019)
6β-Acetoxyscillarenin3-Oβ-D-glucoside (1→4)-α-l-rhamnoside		753.3319	C_38_H_54_O_15_	—	Kakouri et al. (2019)
Chlorogenic acid		355.2257	C_16_H_18_O_9_	1,794,427	Brito et al. (2014)
**Fraction 4.70:10:20 (Butanol: Acetic acid: Water)**					
**Compound**	**Retention time (min)**	**M-H**	**M-H formula**	**Compound CID**	**References**
Psoralen	19.26	187.0934	C_11_H_6_O_3_	6,199	Wu et al. (2010)

**TABLE 3:** Chemical composition of principal compounds in Methanol fractions.

Compound	Retention time (min)	M-H	M-H formula	Compound CID	References
Fraction 1.50:50 (Hexane: Acetone)					
Psoralen	19.23	187.0951	C_11_H_6_O_3_	6,199	Wu et al. (2010)
Fraction 2% Acetone					
Ficuspirolide	8.13	241.0800	C_13_H_20_O_4_	100,987,513	Kuo and Li, (1999)
Hydroxytyrosol	8.73	153.0574	C_8_H_10_O_3_	82,755	Bertelli et al. (2020)
Quercertin derivative	8.92	299.0803	C_15_H_10_O_7_	5,280,343	Danihelová et al. (2013)
Quercertin derivative	9.39	299.0853	C_15_H_10_O_7_	5,280,343	Danihelová et al. (2013)
Vanillic acid 4-Beta-D-Glucoside	10.06	329.0868	C_14_H_18_O_9_	14,132,336	Gong et al. (2019); Navarro et al. (2019)
Vitexin (Apigenin-8-C-glucoside)	14.05	431.1902	C_21_H_20_O_10_	5,280,441	Miao He et al. (2016)
Taxifolin 4′-glucoside	15.54	465.0994	C_21_H_22_O_12_	71,587,141	De Rosso et al. (2014); Navarro et al. (2019)
Eriodictyol 7-O-glucoside	15.83	449.1059	C_21_H_21_O_11_	13,254,473	Areias et al. (2001); Hu et al. (2012)
Psoralen	19.25	187.0970	C_11_H_6_O_3_	6,199	Wu et al. (2010)
Oleuropein	21.07	539.1743	C_25_H_31_O_13_	5,281,544	Longo et al. (2017)
Baccatin III	22.33	603.2449	C_31_H_38_O_11_	65,366	Lee et al. (2014)
Bilobalide		650	C_30_H_35_O_16_	73,581	Longo et al. (2017)
Limonin-17-beta-D-glucoside		649	C_32_H_42_O_14_	24,820,753	Mandadi et al. (2007); Jayaprakasha et al. (2010); Brito et al. (2014)
Fraction 3.50:50 (Acetone: Methanol)					
Hydroxytyrosol	5.45	153.0.90	C_8_H_10_O_3_	82,755	Bertelli et al. (2020)
Protocatechuic acid glucoside		315.0704	C_13_H_16_O_9_	11,972,438	Abu-Reidah et al. (2013)
Pantothenate	5.81	218.1042	C_9_H_16_NO_5_	6,613	Rodríguez-Pérez et al. (2013)
Quercertin derivative	16.16	299.0414	C_15_H_10_O_7_	5,280,343	(Danihelová et al. (2013); Rhimi et al. (2019)
Petunidin 3,5-Di-O-Beta-D-Glucoside	16.20	641.1682	C_28_H_33_O_17_	75,184,857	Li et al. (2019); Wang et al. (2020)
Psoralen	19.22	187.0951	C_11_H_6_O_3_	6,199	Wu et al. (2010)
Oleuropein	21.07	539.1341	C_25_H_32_O1_3_	5,281,544	Longo et al. (2017); Shirzad et al. (2017)
Scillirubroside/scilliphaeosidin-glucoside	22.83	607.2767	C_28_H_31_O_15_	—	Knittel et al. (2014)
Scillaren A	—	693.6864	C_36_H_52_O_13_	441,870	Kakouri et al. (2019)
Unknown	16.06	1,371.4402	C_52_H_75_O_62_	—	—
Glucoscilliphaeoside	24.10	707.3322	C_36_H_52_O_14_	—	Kakouri et al. (2019)
6β-Acetoxyscillarenin3-Oβ-D-glucoside (1→4)-α-l-rhamnoside	24.13	753.3315	C_38_H_54_O_15_	—	Kakouri et al. (2019)
Dihydroquercetin	—	284.0297	C_15_H_12_O_7_	417	—

### 3.8 The *in vitro* Cytotoxicity of *D. calcarata* Fractions Against A549 Cells

#### 3.8.1 Water Fractions

**FIGURE 11:**
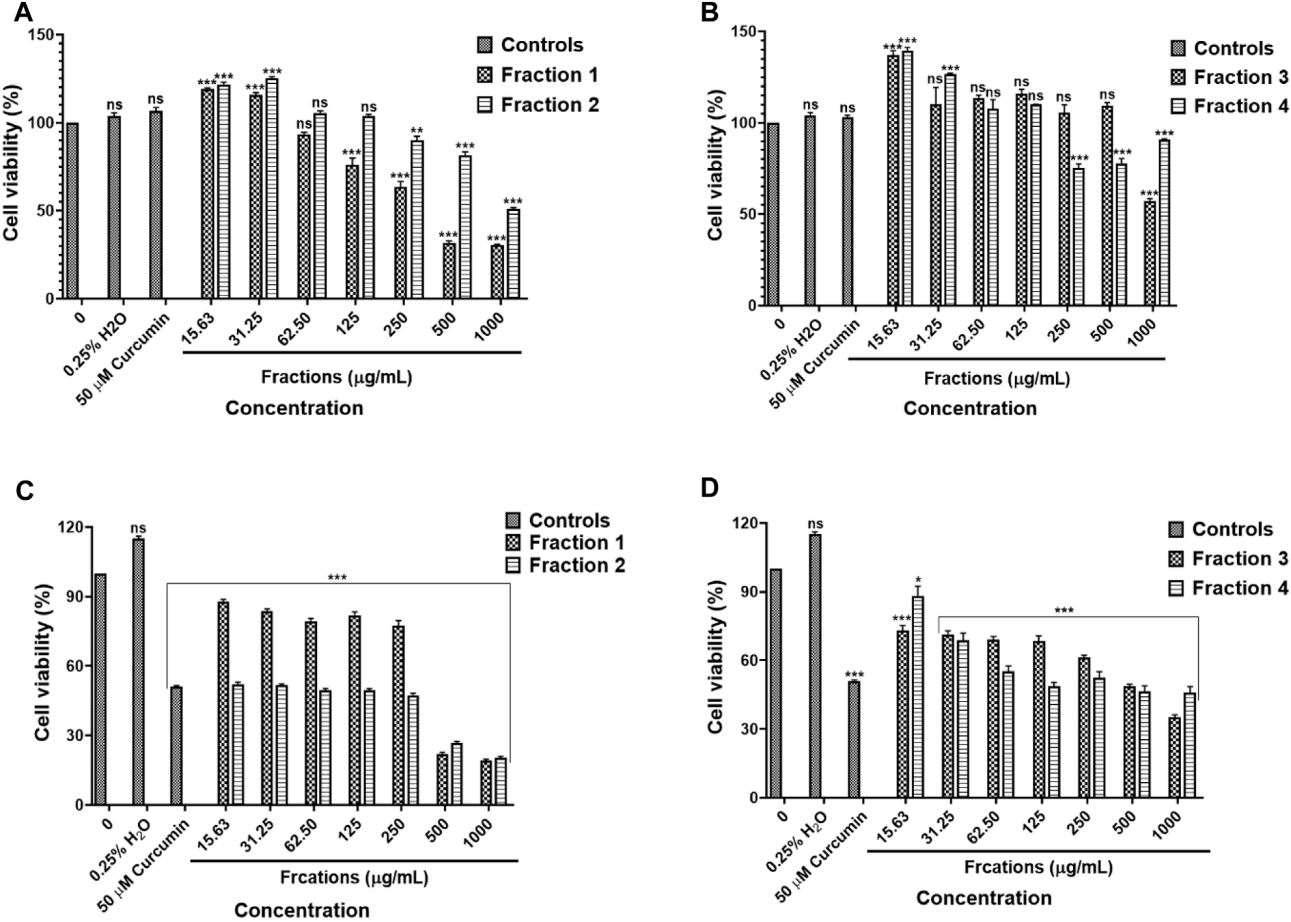
Cytotoxic effect of *D. calcarata* water extract fractions on the human embryonic kidney HEK-293 cells **(A,B)** and lung cancer A549 cells **(C,D)**. Cells were treated with *D. calcarata* water extract fractions at concentrations ranging from 15.63 to 1,000 μg/ml. Curcumin (50 µM) was used as positive control and H_2_O (0.25%) as solvent control. The difference was found to be statistically significant (***p < 0.001) after treatment of HEK-293 cells Fraction 1 (125–1,000 μg/ml), Fraction 2 (250–1,000 μg/ml) and Fraction 4 (250–1,000 μg/ml). Fraction three had no significant (ns) effect on HEK-293 cells (15.63–500 μg/ml). The difference was found to be statistically significant (*p < 0.05 and ***p < 0.001) following treatment of A549 with all fractions compared to the untreated control.

The results in Figures 11A,B, water fractions 2 and 3 showed safety against the human embryonic kidney HEK-293 cells while fraction 1 showed safety after exposure to 15.63–62.50 μg/ml and fraction 4 showed safety following treatment with concentrations 15.63–125 μg/ml, higher concentrations exhibited cytotoxic effects on the HEK-293 cells. An *in vitro* cytotoxicity screening of the *D. calcarata* fractions indicated high cytotoxicity effect on the lung cancer cell line (A549), with the fractions 2, 3, and 4 showing the highest toxicity than fraction 1 (Figures 11C,D). The results are summarised in Supplementary Table S24, S25.

#### 3.8.2 Methanol Fractions

**FIGURE 12:**
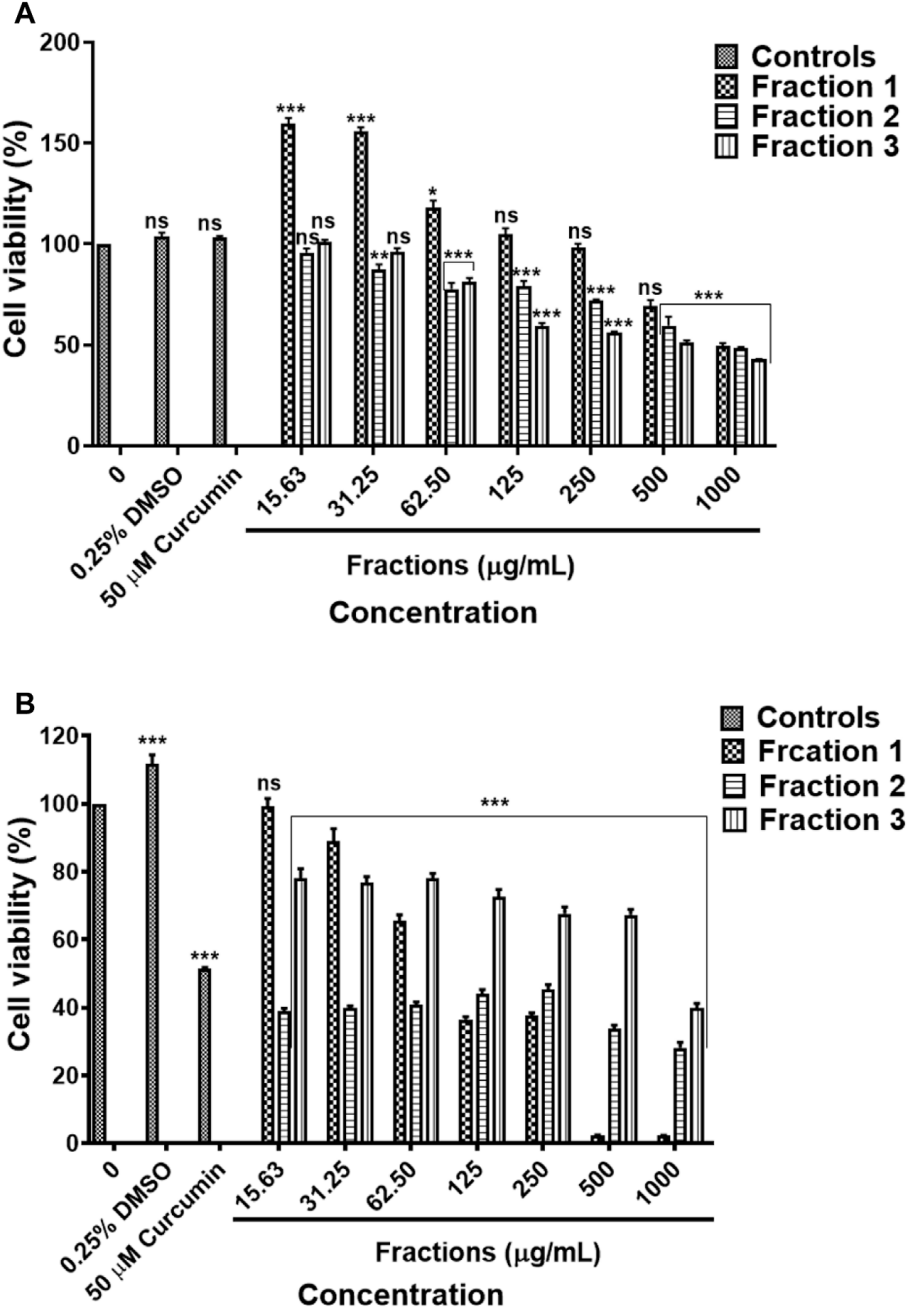
Cytotoxic effect of *D. calcarata* methanol extract fractions on human embryonic kidney HEK-293 cells **(A)** and lung cancer A549 cells **(B)**. Cells were treated with *D. calcarata* methanol extract fractions at concentrations ranging from 15.63 to 1,000 μg/ml. Curcumin (50 µM) was used as positive control and DMSO (0.25%) as solvent control. The difference was found to be statistically significant (*p < 0.05, **p < 0.01, ***p < 0.001) for all fractions comparing with the untreated control.

Results obtained by MTT assay revealed that methanol fraction one did not exhibit a significant effect against HEK-293 following treatment with 15.63–500 μg/ml concentrations of fraction 1. On the other hand, fraction two and three resulted in the cell viability was above 80% following treatment with concentration 15.63–31.25 μg/ml and 15.63–62.50 μg/ml, respectively (Figure 12A). The methanol fractions 1, 2 and 3 exhibited cytotoxicity against and A549 cells, with fraction 1 exhibiting high toxicity than fractions 2 and 3 (Figure 12B). The results are summarised in Supplementary Table S26, S27.

## 4 Discussion

Over 27 million people in South Africa rely on traditional medicine for their health needs (Street et al., 2008). There is poor documentation on the use of South African plants for cancer treatment; however, they have been exploited throughout the world for the development of new and effective anticancer agents (Twilley et al., 2020). Currently, there is no research on the anticancer activities of *D. calcarata* extracts on lung cancer cells. Three human non-small-cell lung carcinoma (NSCLC) cell lines (*p53*-wild type A549, *p53*-mutant H1573 and *p53*-mutant H1437) were used in this study. The data presented in this study demonstrated that methanol extract (ME) exhibited cytotoxic effect against all the lung cancer cell lines, regardless of their *p53* statuses. The methanol (ME) extract was more active against *p53*-mutant lung cancer cells (H1573 and H1437) compared to the *p53*-wild type A549 cells, which exhibited an IC_50_ value of 500 μg/ml, compared to 125 μg/ml for both *p53-*mutant lung cancer cells. The Water extract (WE) was also highly effective against the H1573 and H14347, exhibiting IC_50_ values of 125 and 250 μg/ml, respectively. This is the first time extracts from *Drimia calcarata species* have been tested against lung cancer cells of varying *p53* mutation statuses. However, *Drimia maritima* bulb extract had been previously reported to demonstrate higher cytotoxicity and apoptotic activity compared to the leaves extract on lung A549 cancer cells (Bozcuk et al., 2011).

During apoptosis, a series of modifications, such as chromatin condensation, is generally exhibited by apoptotic cells. To observe apoptotic body formation and nuclear changes that signify apoptosis, A549, H1573 and H1437 cells treated with *D. calcarata* IC_50_ values for 24 h were double stained with acridine orange/ethidium bromide. Previously, Ciniglia et al. (2010) indicated that AO/EB double staining assay can be recommended as a reliable and rapid assay to detect the apoptotic effect-of anticancer compounds or general cell death, since it allows distinction among the viable, early, or late apoptotic cells, based on nuclear morphology variations and chromatin disintegrations. In all three tested cell lines, fluorescence microscopy with AO/EB staining revealed, in *p53*-wild type A549, cells differently stained nuclei (green and orange) exposure to ME and WE (Figures 3E,F). The treatment also caused apoptosis-related morphological changes such as nuclei fragmentation and formation of apoptotic bodies. In the *p53*-mutant H1573 (Figures 4E,F) and *p53*-mutant H1437 (Figures 5E,F) AO/EB staining revealed similar stained cells (green), reduction in cancer cell number and also feature of apoptosis like cell shrinkage and nuclei fragmentation were observed following treatment with ME and WE. For preliminary confirmation of apoptotic/necrotic cell death, AO/EB staining has been used in several studies (Rajavel et al., 2017; Suganya et al., 2019; Khan et al., 2021). This results provide morphological proof that the *Drimia calcarata* extracts induce apoptosis and inhibit cell growth of NSCLC cell lines.

To identify the different pathways of cell death-either necrosis or apoptosis in treated cancer cells with *D. calcarata* extracts, staining with Muse^®^ Annexin V and Dead Cell reagent was used to confirm cell death induction. As shown in Figure 6, the findings indicated that *D. calcarata* ME and WE induced both early and late apoptosis in all the three tested cell lines, *p53*-wild type A549, *p53*-mutant H1573 and *p53*-mutant H1437. Tumours often arise as a result of deregulated mechanisms that are involved in the regulation of cell homeostasis, which include cell cycle arrest, and apoptosis. The latter is an ideal mode for potential anticancer drugs that can be utilized for therapeutic intervention (Ghavami et al., 2009). Cancer chemotherapy and chemoprevention may be improved by agents that inhibit cancer cell proliferation and induce apoptosis. In spite of the development of various anticancer agents, successful cancer treatment is hindered by associated adverse side effects and acquired drug resistant (Khan and Mlungwana, 1999). Thus, growing interest is being shown in the development of novel safe and effective treatments for cancer using plant-based compounds. Additionally, it has been shown that *p53* is one of the most powerful tumour suppressor genes in human cancers, which regulates both intrinsic and extrinsic apoptotic pathways (Maximov and Maximov, 2008). *Bax* and *Bcl-2* genes have been found to be some of the main regulators of apoptosis through the mitochondrial pathway as well as in controlling cytochrome-*c* release (Ashkenazi and Herbst, 2008). Upon an apoptotic stimulus, Bax, proapoptotic Bcl-2 associated X protein, translocates to mitochondrial outer membranes, where it activates cytochrome-*c* release, which then activates caspase-9 and -3 and eventually leads to apoptosis (Thomas et al., 2000). The role of Bcl-2 in this process is different. It can promote mitochondrial integrity or block caspase activation factors for the activation of caspases or regulate apoptosis by interacting with other molecules in the Bcl-2 family (Hengartner, 2000; Jeong and Seol, 2008). Accordingly, a cell’s reaction to apoptotic signals is caused by the ratio between levels of the pro-apoptotic Bax and the antiapoptotic Bcl-2.

In a previous study (Monga et al., 2013), (+)-cyanidan-3-ol (CD-3), isolated from *Acacia catechu* and also detected in *D. calcarata* ME and WE extracts, was shown to upregulate the mRNA and protein levels of Bax, while decreasing the mRNA and protein levels of Bcl-2 in MCF-7 cells. In CD-3-treated MCF-7 cells, cytochrome-*c* was found to be released from the mitochondria. In other words, CD-3 promoted the induction of apoptosis was by modifying the ratio of pro-apoptotic to anti-apoptotic proteins, favouring cell death. Researchers concluded that the up-regulation of Bax and the subsequent decrease in Bcl-2 protein expression may be one of the key mechanisms through which CD-3 induces apoptosis in MCF-7 cells (Monga et al., 2013). Several compounds including bilobalide, limonin and protocatechuic acid detected in *D. calcarata* have been reported to inhibit cell proliferation and induce apoptosis in various cancer cells. Bilobalide isolated from the leaves of *Ginkgo biloba* inhibited cell proliferation and induced apoptosis in FaDu human pharyngeal squamous cell carcinoma via both the death receptor-mediated extrinsic apoptotic pathway and the mitochondrial-mediated intrinsic apoptotic pathway (Jeong et al., 2020). The *in vitro* results proved that bilobalide effectively suppressed the gastric cancer cell growth and induced cell death by nuclear damage and apoptosis induction (Liu et al., 2021). Limonin isolated from *Poncirus trifoliata rafin* seeds induced apoptosis through the upregulation of proapoptotic protein Bax and downregulation of anti-apoptotic protein Bcl-2 in HCT-15 and SNU 449 cells in a dose-dependent manner (Rahman et al., 2015). A purchased protocatechuic acid reduced the growth rate of three non-small lung cancer cells, A549, H3255 and Calu-6; and increased Bax expression and reduced Bcl-2 expression (Tsao et al., 2014).

In order to examine the mechanism of apoptosis-induction during 24-h incubation of lung cancer cells with *D. calcarata* extracts, RT-PCR was used to measure the expression of several apoptotic genes, including *p53*, *Bcl-2* and *Bax.* In WE-treated H1437 cells, apoptosis was associated with increased mRNA expression of *p53*, whereas in A549 and H1573 cells, the mRNA expressions of *Bcl-2* and *p53* were decreased. As a result, *D. calcarata* WE induced the *p53*-dependent apoptosis in H1437, ME induced the *p53*-independent apoptosis in A549 and both ME and WE induced the *p53*-independent apoptosis in H1573. The A549 cells were not undergoing as much apoptotic cell death as the other lung cancer cells while the water extract showed no toxicity. *D. calcarata* extracts selectively affect the growth of human non-small lung cancer cells.

The chemotherapeutic drug cisplatin modulates the JAK/STATS pathway by dephosphorylating it in cancer cells. Additionally, numerous platinum-containing compounds disrupt STAT3 signalling and interfere with its biochemical activities (Song et al., 2004; Turkson et al., 2005; Thoennissen et al., 2009). Our results demonstrate that *D. calcarata* ME, downregulated the expression of *STAT1*, *STAT3* and *STAT5B*, and upregulated expression of *STAT5A* gene in A549. It has been shown that activated STAT5 reduces antitumour immunity and increases tumour proliferation, invasion, and survival (Baśkiewicz-Masiuk and Machaliński, 2004; Rani and Murphy, 2016). Previously, STAT5A partly inhibited the apoptosis induced by miR-1469 in lung cancer cells, A549 and NCI-H1650 (Xu et al., 2015). A possible explanation for the poor apoptosis rate in the present study can be found in the upregulation of STAT5A. Thus, STAT5A may prevent ME induced apoptosis and serve as a drug resistant mechanism of lung cancer A549 cells in response to water extract.

*STAT1*, *STAT3*, *STAT5A* and *STAT5B* genes were increased by both extracts in H1437. ME decreased *STAT1*, *STAT3*, *STAT5A* and *STAT5B* in H1573 cells, while treatment with WE lead to significant increase of *STAT1*, *STAT3*, *STAT5A*, while no significant change in STAT5B gene expression.

Crosstalk exists between the JAK-STAT pathway and *p53* function. STAT1 decreases the expression of MDM2, thus stabilizing *p53* (Townsend et al., 2004). STAT1 binds *p53* and accentuates transcriptional effects of *p53* on certain *p53*-responsive apoptotic genes like Bax (Aladjem et al., 1998). Another study showed that persistently activated STAT3 may disable *p53* without the requirement for *p53* mutations. Activated STAT3 interacts with the promoter of the *p53* gene, inhibiting *p53* expression (Niu et al., 2005). Furthermore, blocking STAT3 in cancer cells up-regulates expression of *p53*, leading to *p53*-mediated tumour cell apoptosis. As a point of convergence for many oncogenic signalling pathways, STAT3 is constitutively activated at high frequency in a wide diversity of cancers including lung cancer (Sen et al., 2020) and is a promising molecular target for cancer therapy (Niu et al., 2005). Thus, repression of *p53* expression by STAT3 is likely to have an important role in development of tumours and targeting STAT3 represents a novel therapeutic approach for *p53* reactivation in many cancers lacking *p53* mutations (Niu et al., 2005). Our results indicate that the inhibitory proliferative effect of *D. calcarata* extracts on *p53*-wildtype A549 and *p53*-mutated H1573 non-small lung cancer cells is correlated with the suppression of *Bcl-2*, *STAT3* and *STAT5B* while that is not the case in *p53*-mutant H1437 non-small lung cancer cells. Thus, our results suggest that the dysregulation of anti-apoptotic molecules *Bcl-2*, *STAT3*, *STAT5A* and *STAT5B* in H1437 by *D. calcarata* may play a role in the prolongation of cell survival, which may subsequently contribute to the development of *p53*-mutated non-small human lung cancer H1437 cells. A more profound understanding of the potential interaction between *p53* and activated STATs is necessary in order to take full advantage of novel *p53* targeted therapies.

To our knowledge this is the first report of *D. calcarata* methanol and water extracts fractions, their chemical compositions and their cytotoxicity against lung cancer A549 cells as well as their safety on the human embryonic kidney HEK-293 cells. Previously, methanol extract and water extract chemical compositions were reported and the extracts showed safety against human embryonic kidney HEK-293 cells. Compounds detected in both extracts included dihydrophaseic acid hexoside, 2-hydroxyethyl 4-acetyl-4-methyl-5-oxohexanoate, limonin-17-beta-d-glucoside (1-) and 6β-Acetoxyscillarenin3-Oβ-d-glucoside (1→4)-α-l-rhamnoside. Psoralene was only detected in the water extract (Laka et al., 2021). In the present study, all the *D. calcarata* fractions contain a similar compound psoralen with the chemical formula C_11_H_6_O_3_ (M-H formula: 187.09). Many reports have also confirmed that psoralen has the potential to eliminate various human cancer cells (Wang et al., 2011; Wu et al., 2013). Previously, Wu et al. (2013) reported that psoralen showed significant antiproliferative activity against the HepG-2 and C6 cancer cell lines. Furthermore, psoralen significantly inhibited cell proliferation by inducing G0/G1 phase arrest in MCF-7 cells and G2/M phase arrest in MDA-MB-231 cells (Wang et al., 2018). Thus, psoralen might be the main key compound responsible for the cytotoxic activity of the *D. calcarata*. The search for anticancer agents from natural sources has been successful worldwide, and active constituents have been isolated and are nowadays used to treat human tumours. The ethnopharmacological knowledge is helpful to lead the search for plants with potential cytotoxic activity. Thus, the search for new drugs is imperative and the results of our investigation call for future isolation and characterization of the active constituents in *D. calcarata* extracts.

## 5 Conclusion

The water extract showed cytotoxicity against both the *p53*-mutant H1437 and H1573 cell lines and no effect on the *p53*-wild type A549 cell line, whereas the methanol extract exhibited cytotoxic effects against both the *p53*-mutant and *p53*-wild type lung cancer cells. The A549 cells were less susceptible than the *p53*-mutant cell lines, H1573 and H1437. *D. calcarata* extracts selectively affect the growth of human non-small lung cancer cells. The growth inhibition of human lung cancer cells mediated by *D. calcarata* extracts appears to be associated with apoptosis and G0/G1 and S-phase cell cycle arrest and altered expression of the tumour suppressor and anti-apoptotic genes *p53* and *Bcl-2*, cancer-related genes, especially those that are involved in both cell cycle (*p21*, *CLB1* and *CDC2*) and transcriptional factors, such as STATs. Our results indicate that the proliferative inhibitory effect of *D. calcarata* extracts on *p53*-wildtype A549 and *p53*-mutated H1573 non-small lung cancer cells correlated with the suppression of *Bcl-2*, STAT3 and STAT5B while that is not the case in *p53*-mutant H1437 non-small lung cancer cells. Thus, our results suggest that the regulation of anti-apoptotic molecules *Bcl-2*, *STAT3*, *STAT5A* and *STAT5B* in H1437 by *D. calcarata* may play a role in the prolongation of cell survival, which may subsequently contribute to the development of *p53*-mutated non-small human lung cancer H1437 cells. The *D. calcarata* water and methanol fractions reduced the cell viability of A549 cells. In LC-MS profiling, the major compounds produced by *D. calcarata* water and methanol fractions was identified as psoralen and an identified compound might be the reason behind this plant’s anticancer activities. Additionally, our results indicate that *D. calcarata* gives most promising results as an anticancer agent for the *p53*-mutant human non-small lung cancer cells than the wild type-*p53* human non-small lung cancer cells.

## Data Availability

The original contributions presented in the study are included in the article/Supplementary Material, further inquiries can be directed to the corresponding author.
