# Supplementary material for: P53-Related Anticancer Activities of Drimia calcarata Bulb Extracts Against Lung Cancer
Source: Front Mol Biosci. 2022 Jun 13;9:876213. doi: 10.3389/fmolb.2022.876213 (PMC9235921; doi:10.3389/fmolb.2022.876213)
Supplement: Supplementary file 1 [file DataSheet1.PDF]

SUPPLEMENTARY DATA

MTT assay

Table S1: The MTT assay average percentages standard error of mean (SEM) of MRC-5 cells after 24 h treatment with *D. calcarata* extracts.

Treatment (µg/mL)	Mean ± SEM	
	**ME**	**WE**
0	100.00±0.000	100.00±0.000
0.25% DMSO	114.00±1.291	114.00±1.291
0.25% H_2O	113.67±0.955	113.67±0.955
50 µM Curcumin	112.67±0.667	112.67±0.667
15.63	95.000±1.033	97.500±2.094
31.25	94.667±0.803	100.00±2.805
62.50	94.667±0.494	101.00±3.011
125	93.500±0.885	103.83±0.872
250	95.167±0.749	102.00±2.530
500	98.500±0.671	104.00±2.082
1000	105.17±1.014	124.67±2.290

Table S2: The MTT assay average percentages standard error of mean (SEM) of A549 cells after 24 h treatment with *D. calcarata* extracts.

Treatment (µg/mL)	Mean ± SEM	
	**ME**	**WE**
0	100.00±0.000	100.00±0.000
0.25% DMSO	104.23±3.100	104.23±3.100
0.25% H_2O	104.51±3.922	104.51±3.922
50 µM Curcumin	50.716± 0.7583	55.647± 1.837
15.63	76.222±3.197	74.694±3.137
31.25	66.051±1.208	72.670±2.334
62.50	65.358± 1.722	74.140±4.580

125	61.975±2.011	76.328±4.751
250	56.860±1.331	79.173± 4.762
500	52.948±1.569	86.951±6.619
1000	42.256±1.339	80.375±3.506

Table S3: The MTT assay average percentages standard error of mean (SEM) of H1573 cells after 24 h treatment with *D. calcarata* extracts.

Treatment (µg/mL)	Mean (%) ±SEM	
	**ME**	**WE**
0	100.00±0.000	100.00±0.000
0.25% DMSO	102.50±0.806	102.50±0.806
0.25% H_2O	105.33±1.563	105.33±1.563
50 µM Curcumin	60.600± 1.030	60.600± 1.030
15.63	57.333±2.108	70.833±2.227
31.25	51.833±1.376	68.500±1.384
62.50	50.000± 1.826	62.833±0.9458
125	49.000±1.807	47.667±2.348
250	36.833±1.327	49.667±0.9189
500	42.500±1.455	57.333±1.282
1000	62.667±2.376	62.833±1.851

Table S4: The MTT assay average percentages standard error of mean (SEM) of H1437 cells after 24 h treatment with *D. calcarata* extracts.

Treatment (µg/mL)	Mean (%) ±SEM	
	**ME**	**WE**
0	100.00±0.000	100.00±0.000
0.25% DMSO	109.33± 3.792	109.33± 3.792
0.25% H_2O	114.00±4.171	114.00±4.171
50 µM Curcumin	53.333± 0.9189	53.333±0.9189
15.63	73.833±0.7491	74.667±1.202

31.25	60.833±2.915	70.167±2.136
62.50	52.667± 2.108	59.000±0.5164
125	55.000±2.113	56.167±1.470
250	55.333±2.305	58.167±1.138
500	56.000±2.338	59.833±1.014
1000	55.167±1.222	62.00±1.000

Muse Cell viability

Table S5: The Muse® Count and Viability mean percentages and SEM of MRC-5 cells.

Treatment	Mean (%) ±SEM			
	**Live**		**Dead**	
	**ME**	**WE**	**ME**	**WE**
Control	99.900±0.577	99.900±0.577	0.100±0.058	0.100±0.058
0.25% DMSO	99.800±0.577	99.800±0.577	0.200±0.058	0.200±0.058
0.25% H_2O	99.933±0.033	99.933±0.033	0.067±0.033	0.067±0.033
50 µM Curcumin	99.933±0.067	99.933±0.067	0.033±0.033	0.033±0.033
62.50 µg/mL	100.00±0.000	99.933±0.033	0.000±0.000	0.037±0.032
125 µg/mL	99.933±0.033	99.967±0.033	0.067±0.033	0.033±0.033
250 µg/mL	100.00±0.000	100.00±0.000	0.000±0.000	0.000±0.000
500 µg/mL	100.00±0.000	100.00±0.000	0.000±0.000	0.000±0.000
1000 µg/mL	99.967±0.058	99.967±0.058	0.033±0.033	0.033±0.033

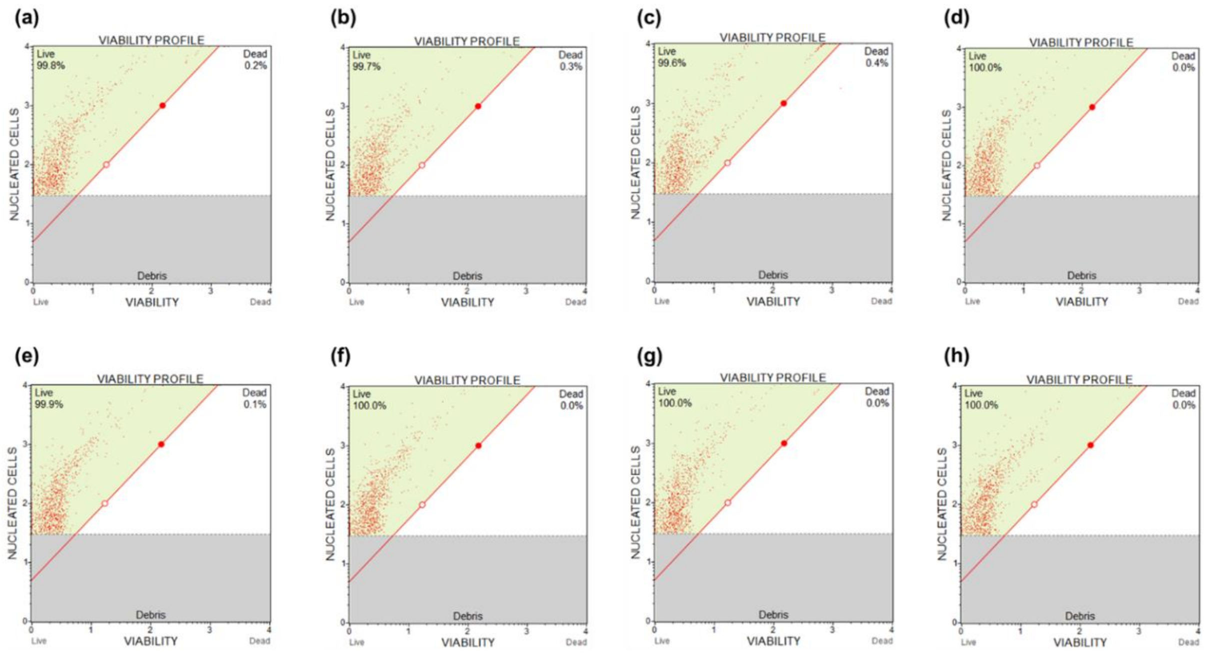

Figure S1. Muse® Count and Viability profiles of the MRC-5 cells after 24 h treatment with ME: (a) Untreated cells, (b) 0.25% H_2O, (c) 50 µM Curcumin, (d) 62.50 µg/mL, (e) 125 µg/mL, (f) 250 µg/mL, (g) 500 µg/mL and (h) 1000 µg/mL cells treated *D. calcarata* water extract. Muse® Cell Analyser was used to analyse the results.

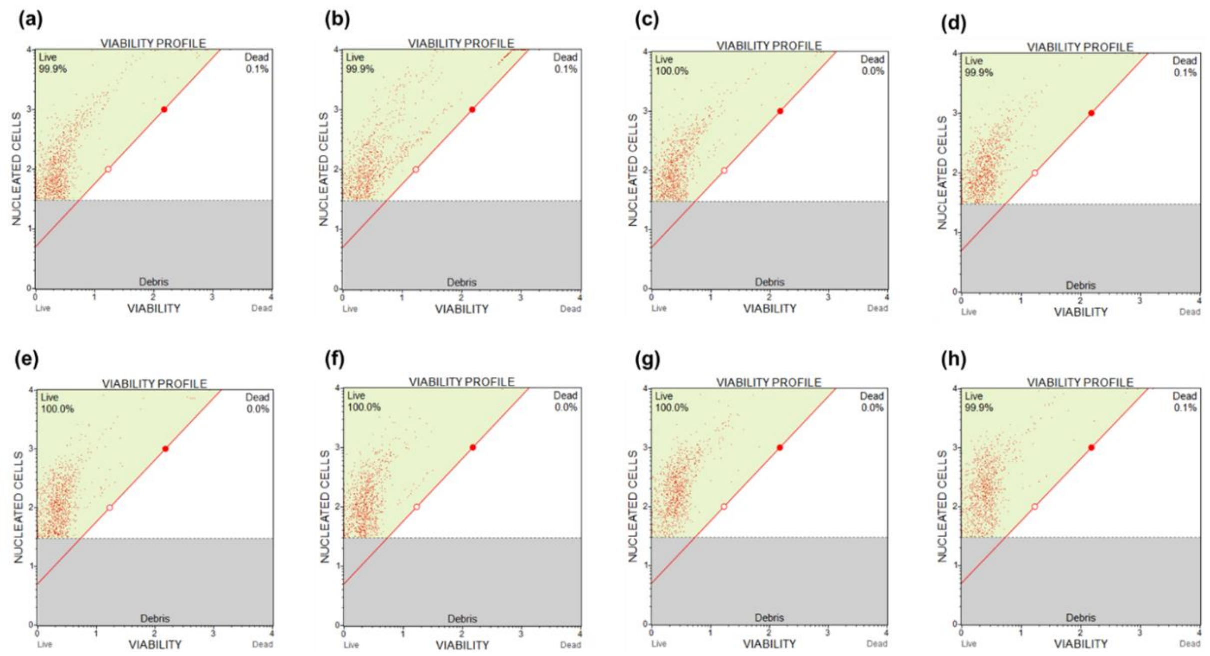

Figure S2. Muse® Count and Viability profiles of the MRC-5 cells after 24 h treatment: (a) Untreated cells, (b) 0.25% DMSO, (c) 50 µM Curcumin, (d) 62.50 µg/mL, (e) 125

µg/mL, (f) 250 µg/mL, (g) 500 µg/mL and (h) 1000 µg/mL cells treated *D. calcarata* methanol extract. Muse® Cell Analyser was used to analyse the results.

Table S6: The Muse® Count and Viability mean percentages and SEM of A549 cells.

Treatment	Mean (%) ±SEM	
	**Live**	**Dead**
Control	89.340± 2.524	10.660± 2.524
0.25% DMSO	85.200±1.457	14.800±1.457
0.25% H_2O	84.350±1.250	15.650±1.250
50 µM Curcumin	38.200±2.800	62.250±3.250
500 µg/mL ME	51.550±1.267	48.450±1.267
500 µg/mL WE	80.000±5.056	20.000±5.056

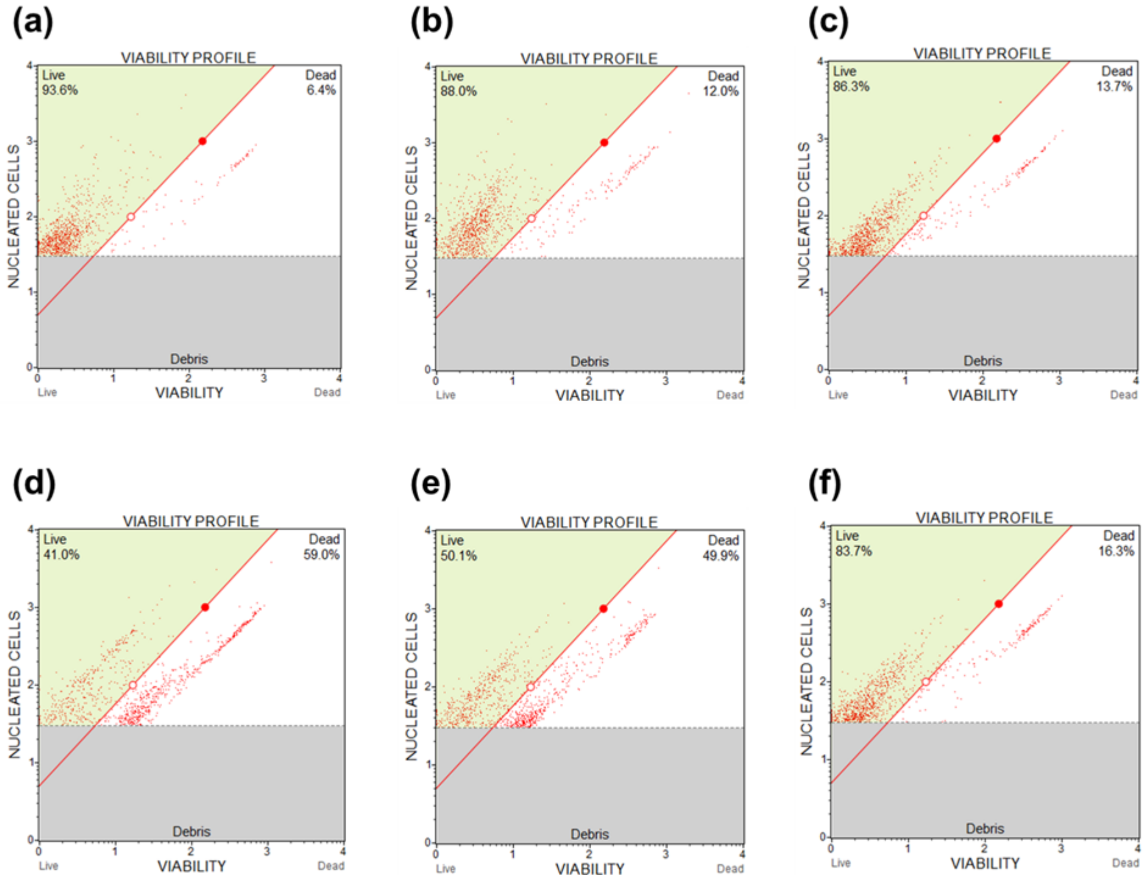

Figure S3. The Muse® cell viability profiles of A549 cells after 24 h treatment with *D. calcarata* extracts: (a) Control, (b) 0.25% DMSO, (c) 0.25% H_2O, (d) 50 µM curcumin, (e) 500 µg/mL ME and (f) 500 µg/mL WE. Muse® Cell Analyser was used to analyse the results.

Table S7: The Muse® Count and Viability mean percentages and SEM of H1573 cells.

Treatment	Mean (%) ±SEM	
	**Live**	**Dead**
Control	90.233±0.467	9.500±0.503
0.25% DMSO	85.733±0.203	14.267±0.203
0.25% H_2O	95.067±0.410	4.933±0.410
50 µM Curcumin	61.233±0.617	38.767±0.617
125 µg/mL ME	52.733±0.203	47.267±0.203
125 µg/mL WE	59.700±2.488	40.300±2.488

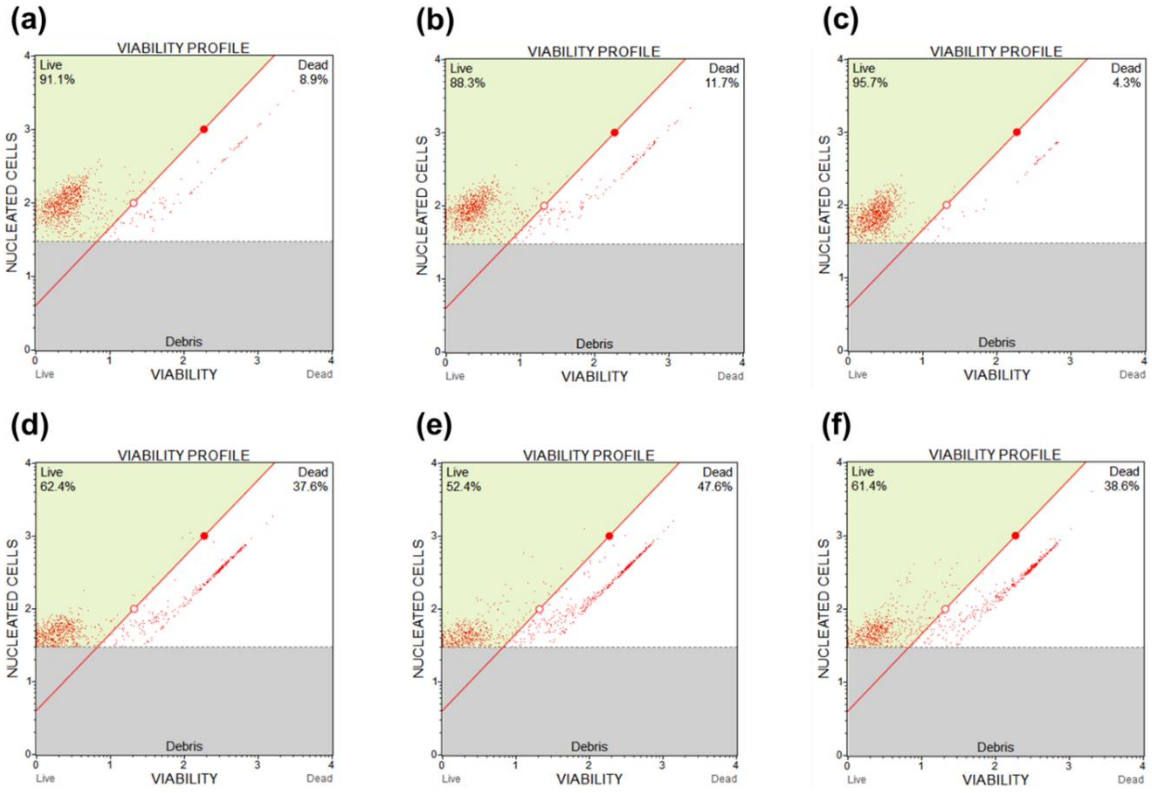

Figure S4. The Muse® cell viability profiles of H1573 cells after 24 h treatment with *D. calcarata* extracts: (a) Control, (b) 0.25% DMSO, (c) 0.25% H_2O, (d) 50 µM curcumin, (e) 125 µg/mL ME and (f) 125 µg/mL WE. Muse® Cell Analyser was used to analyse the results.

Table S8: The Muse® Count and Viability mean percentages and SEM of H1437 cells.

Treatment	Mean (%) ±SEM	
	**Live**	**Dead**
Control	88.400±2.400	11.600±2.400
0.25% DMSO	84.450±5.550	15.550±5.550
0.25% H_2O	88.750±1.850	11.250±1.850
50 µM Curcumin	47.967±7.514	52.033±7.514
62.50 µg/mL ME	32.295±1.661	66.800±1.597
125 µg/mL WE	54.824±1.176	45.175±1.176

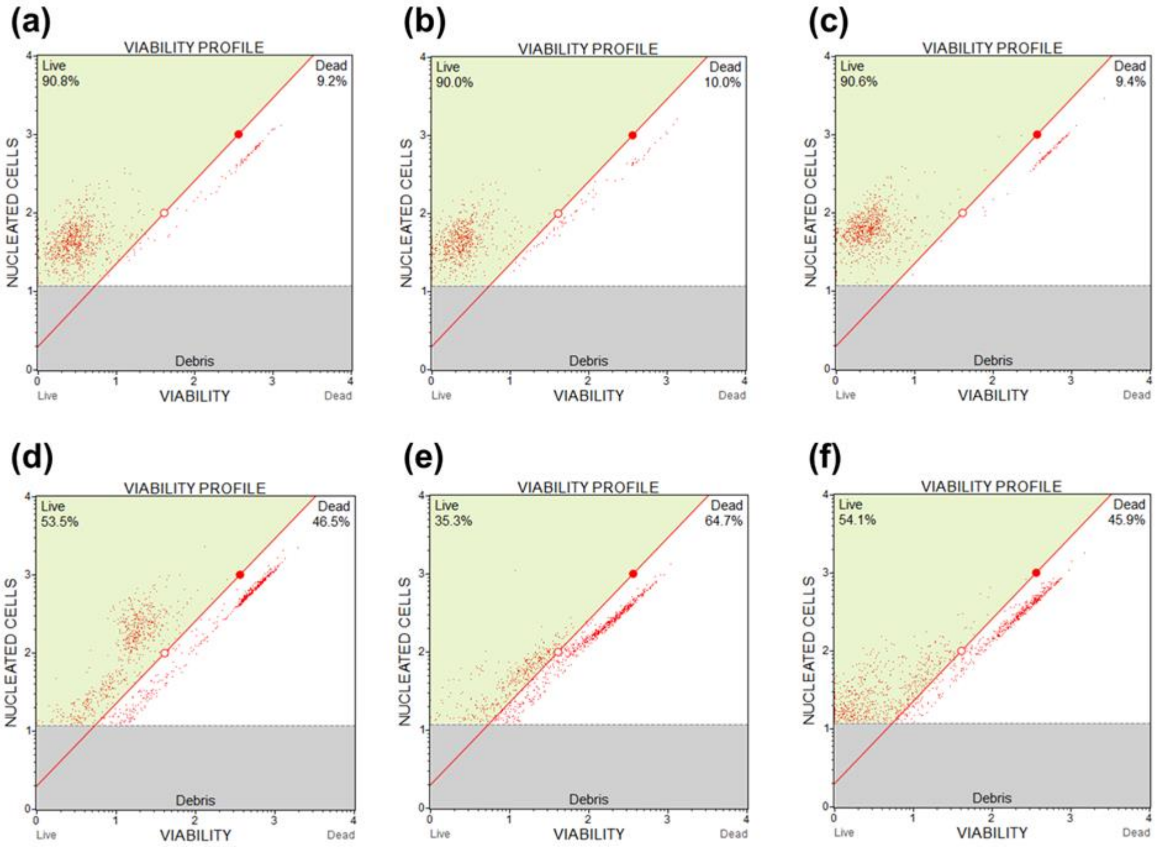

Figure S5. The Muse® cell viability profiles of H1437 cells after 24 h treatment with *D. calcarata* extracts: (a) Control, (b) 0.25% DMSO, (c) 0.25% H_2O, (d) 50 µM

curcumin, (e) 62.50 µg/mL ME and (f) 125 µg/mL WE. Muse® Cell Analyser was used to analyse the results.

Annexin V

Table S9: The apoptosis mean percentages and SEM of A549 cells.

Treatment	Mean (%) ±SEM				
	**Live**	**Early apoptotic**	**Late apoptotic**	**Dead**	**Total apoptotic**
Control	92.668±0.589	7.332±0.589	0.000±0.000	0.000±0.000	7.332±0.589
0.25% DMSO	87.300±0.671	10.683±0.885	2.300±0.638	0.050±0.000	12.650±0.671
0.25% H_2O	87.400±1.526	7.450±0.448	5.100±0.757	0.050±0.029	12.550±1.100
50 µM Curcumin	67.578±1.422	17.870±1.001	8.763±1.472	3.760±0.914	25.825±2.698
500 µg/mL ME	74.035±0.955	18.282±1.278	6.185±1.094	1.148±0.374	24.456±1.467
500 µg/mL WE	74.035±0.955	13.943±1.249	7.603±0.864	4.418±1.084	21.557±0.771

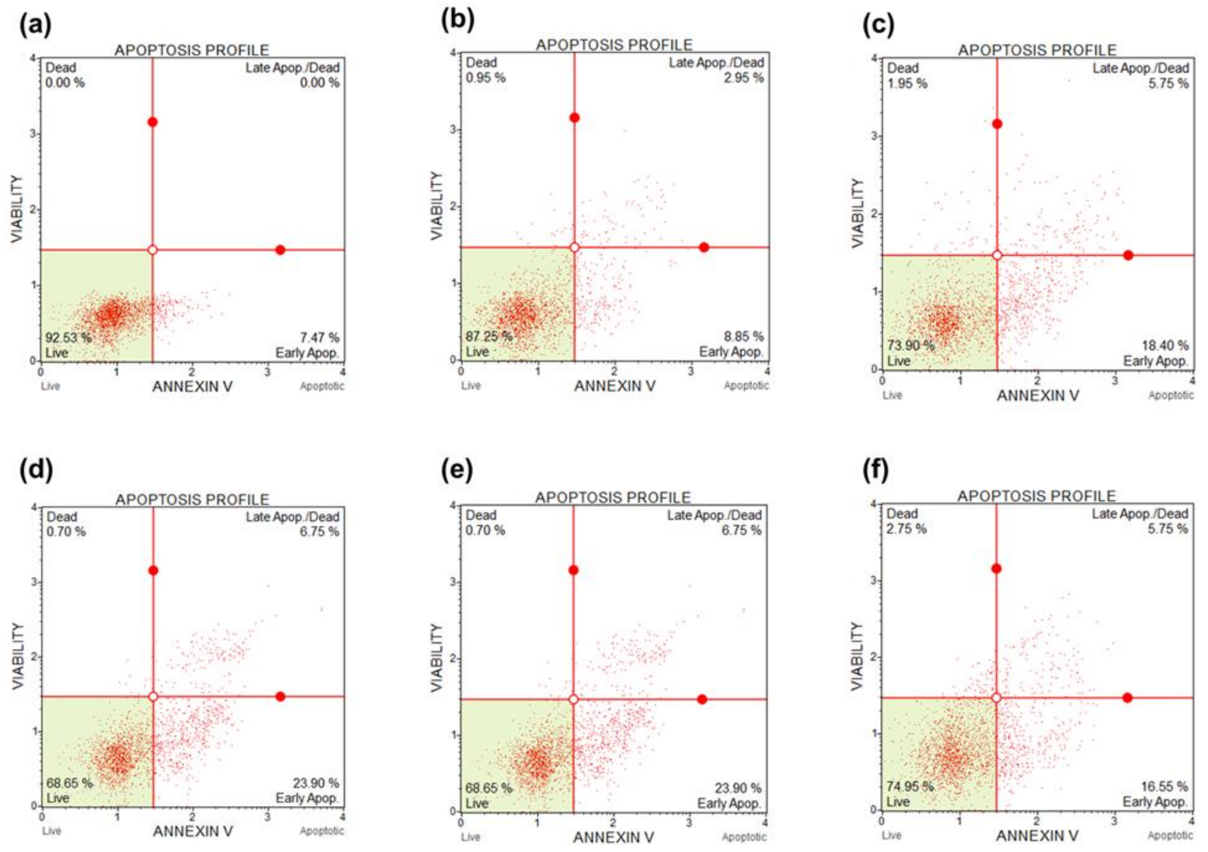

Figure S6. Apoptosis profiles of A549 cells after 24 h treatment: (a) Control, (b) 0.25% DMSO, (c) 0.25% H_2O, (d) 50 µM curcumin, (e) 500 µg/mL ME and (f) 500 µg/mL WE. Muse® Cell Analyser was used to analyse the results.

Table S10: The apoptosis mean percentages and SEM of H1573 cells.

Treatment	Mean (%) ±SEM				
	**Live**	**Early apoptotic**	**Late apoptotic**	**Dead**	**Total apoptotic**
Control	85.726±0.691	10.870±0.645	2.342±0.273	1.210±0.176	13.214±0.869
0.25% DMSO	85.157±2.220	11.720±2.127	1.883±0.198	1.240±0.132	13.607±2.322
0.25% H_2O	82.900±0.201	12.575±0.215	2.527±0.240	1.737±0.155	15.636±0.049
50 µM Curcumin	52.830±1.541	26.575±1.355	20.457±1.461	0.138±0.067	47.033±1.540

125 µg/mL ME	52.393±1.833	36.095±0.709	10.927±1.547	0.587±0.181	47.022±1.883
125 µg/mL WE	39.700±3.073	41.586±1.960	18.528±1.300	0.080±0.036	60.112±3.069

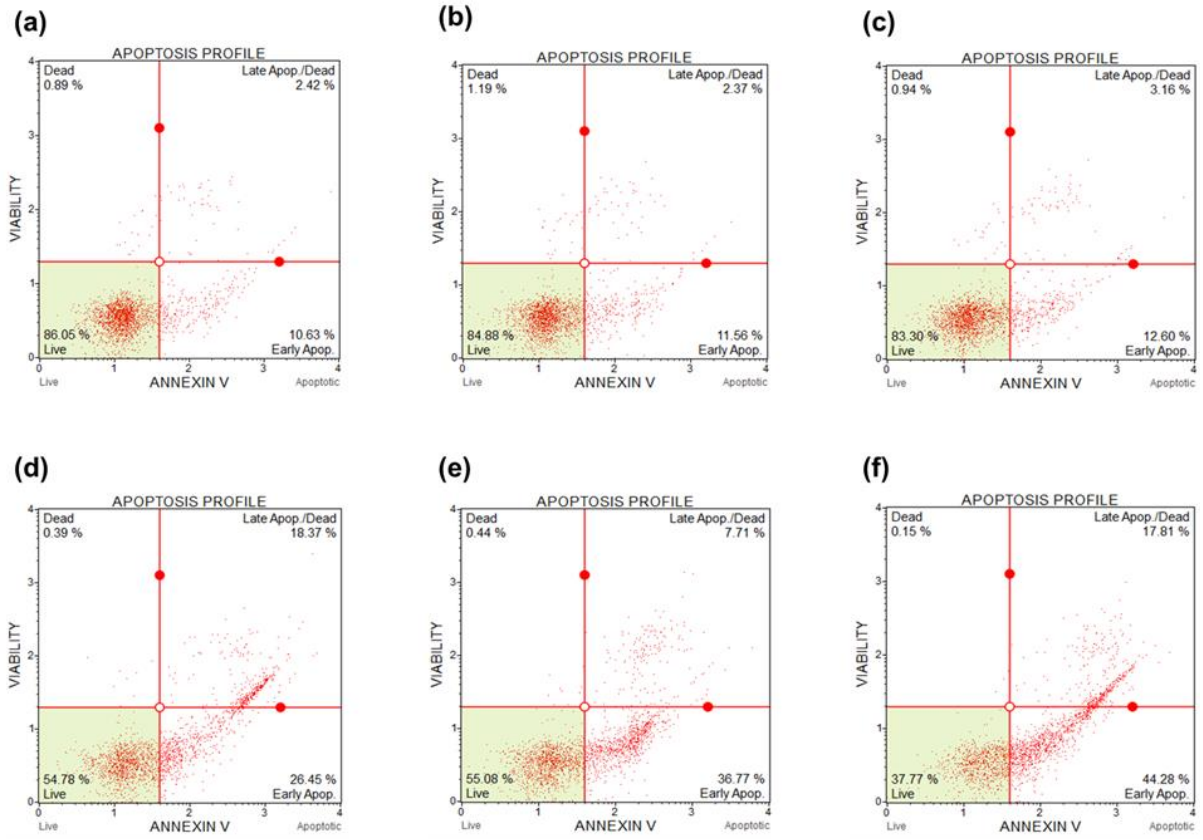

Figure S7. Apoptosis profiles of H1573 cells after 24 h treatment: (a) Control, (b) 0.25% DMSO, (c) 0.25% H_2O, (d) 50 µM curcumin, (e) 125 µg/mL ME and (f) 125 µg/mL WE. Muse® Cell Analyser was used to analyse the results.

Table S11: The apoptosis mean percentages and SEM of H1437 cells.

Treatment	Mean (%) ±SEM				
	**Live**	**Early apoptotic**	**Late apoptotic**	**Dead**	**Total apoptotic**
Control	83.260±1.003	13.782±1.163	2.510±0.158	0.328±0.111	16.412±1.074

0.25% DMSO	84.742±0.927	12.106±0.403	2.582±0.539	0.450±0.088	14.808±0.993
0.25% H_2O	82.950±2.075	13.475±2.046	3.350±0.029	0.588±0.139	16.463±2.188
50 µM Curcumin	56.902±4.045	22.385±2.397	19.098±2.136	0.333±0.025	41.487±3.664
62.50 µg/mL ME	59.270±2.564	23.700±2.101	16.263±0.429	0.117±0.051	40.065±2.212
125 µg/mL WE	62.114±0.974	24.374±0.848	13.464±0.329	0.032±0.011	37.836±0.972

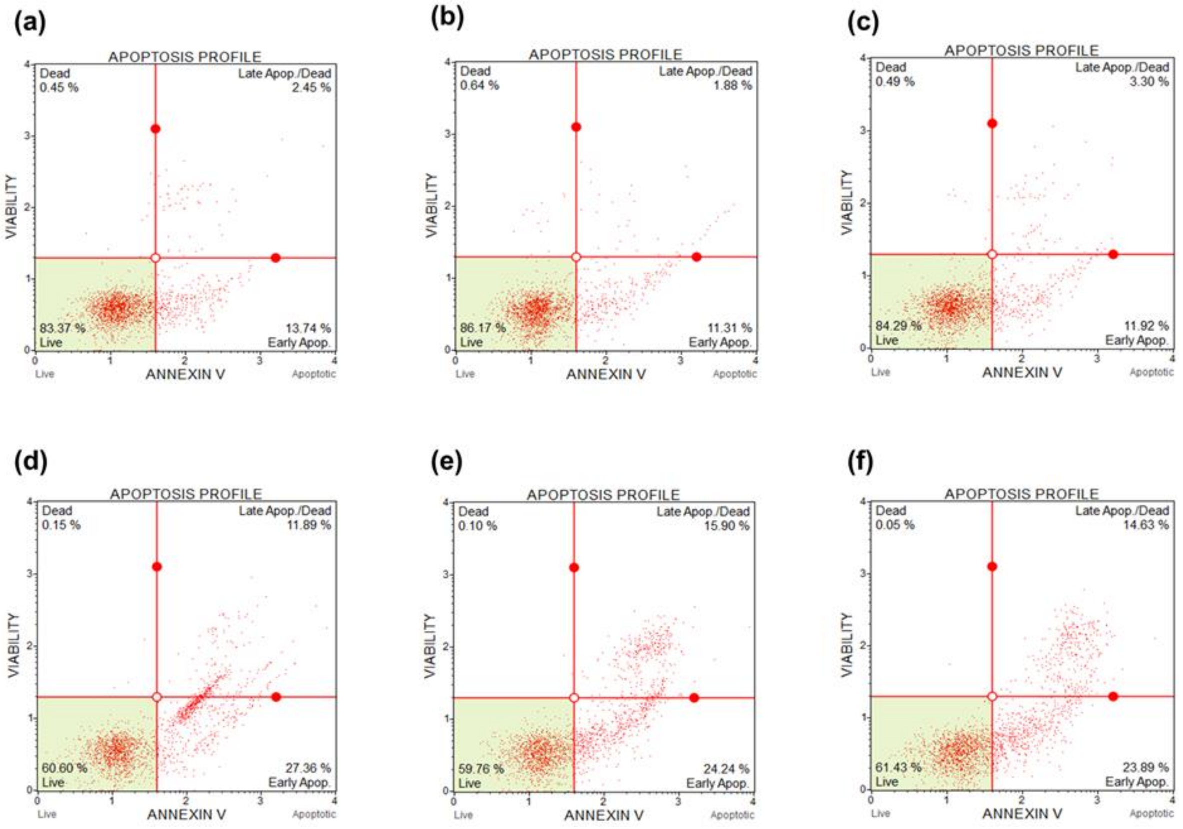

Figure S8. Apoptosis profiles of H1437 cells after 24 h treatment: (a) Control, (b) 0.25% DMSO, (c) 0.25% H_2O, (d) 50 µM curcumin, (e) 62.50 µg/mL ME and (f) 125 µg/mL WE. Muse® Cell Analyser was used to analyse the results.

Apoptosis related genes

Table S12: The mean densities and SEM of apoptotic genes in A549 cells.

Treatment		Mean (INT/mm^2) ±SEM			
	Bax	***Bcl-2***	***p53* Variant 1**	***p53* Variant 2**	***GAPDH***
**0**	0.000±0.000	6.592±0.007	0.277±0.067	0.736±0.029	4.568±0.019
**0.25% DMSO**	0.000±0.000	8.671±0.027	0.251±0.104	0.715±0.025	4.683±0.050
**0.25% H_2O**	0.000±0.000	6.159±0.014	0.565±0.057	2.694±0.037	4.630±0.047
**500 µg/mL ME**	0.000±0.000	6.095±0.011	0.000±0.000	0.031±0.00	4.509±0.051
**50 µM Curcumin**	0.000±0.000	6.828±0.017	0.000±0.000	0.028± 0.006	4.555±0.069
**HEK-293**	0.000±0.000	0.1623±0.015	0.000±0.000	0.010±0.005	4.540±0.023

**Blank**	0.000±0.000	0.000±0.000	0.000±0.000	0.000±0.000	0.000±0.000

Table S13: The mean densities and SEM of apoptotic genes in H1573 cells.

**Treatment**		**Mean (INT/mm²) ±SEM**			
	Bax	***Bcl-2***	***p53* Variant 1**	***p53* Variant 2**	***GAPDH***
**0**	0.000±0.000	14.490±0.032	10.973±0.071	4.451±0.236	11.219±0.120
**0.25% DMSO**	0.000±0.000	15.045± 0.041	9.305± 0.026	4.128± 0.063	11.422±0.132
**0.25% H_2O**	0.000±0.000	15.201±0.048	9.470±0.032	3.973±0.098	11.548± 0.117
**125 µg/mL ME**	0.000±0.000	5.539±0.065	7.673±0.052	2.807±0.048	11.973±0.147
**125 µg/mL WE**	0.000±0.000	0.583± 0.091	2.514±0.037	1.257±0.078	11.600±0.139
**50 µM Curcumin**	0.000±0.000	17.197±0.062	8.856±0.032	3.794±0.100	11.742±0.142

**HEK-293**	0.000±0.000	0.2837± 0.021	0.000±0.000	0.000±0.000	11.224±0.137
**Blank**	0.000±0.000	0.000±0.000	0.000±0.000	0.000±0.000	0.000±0.000

Table S14: The mean densities and SEM of apoptotic genes in H1437 cells.

**Treatment**		**Mean (INT/mm^2) ±SEM**			
	Bax	***Bcl-2***	***p53* Variant 1**	***p53* Variant 2**	**GAPDH**
**0**	0.000±0.000	0.5947±0.056	2.185±0.044	0.727±0.028	9.817±0.083
**0.25% DMSO**	0.000±0.000	0.660±0.047	2.309±0.039	0.918±0.030	9.937±0.016
**0.25% H_2O**	0.000±0.000	1.089± 0.045	2.350±0.022	1.005±0.0157	9.985±0.005
**62.50 µg/mL ME**	0.000±0.000	10.219±0.069	6.790±0.101	1.005±0.0157	10.155±0.061
**125 µg/mL WE**	0.000±0.000	13.647±0.173	2.940± 0.425	10.078±0.748	10.052±0.046

**50 µM Curcumin**	0.000±0.000	13.869± 0.106	15.532±0.220	0.06167± 0.005	10.128±0.048
**HEK-293**	0.000±0.000	0.000±0.000	0.000±0.000	0.000±0.000	9.800±0.089
**Blank**	0.000±0.000	0.000±0.000	0.000±0.000	0.000±0.000	0.000±0.000

Cell cycle

Table S15: The cell cycle mean percentages and SEM of A549 cells.

Treatment	Mean (%) ±SEM		
	**G0/G1**	**S**	**G2/M**
Control	51.000±2.556	4.833±1.276	42.500±1.335
0.25% DMSO	56.167±2.386	6.500±1.335	35.833±3.790
0.25% H_2O	58.950±0.650	4.600±0,400	36.450±0,250
50 µM Curcumin	58.500±1.408	4.833±0.401	36.000±1.571
500 µg/mL ME	58.667±2.060	8.833±1.579	31.333±2.940
500 µg/mL WE	53.333±0.494	6.333±0.494	38.500±0.992

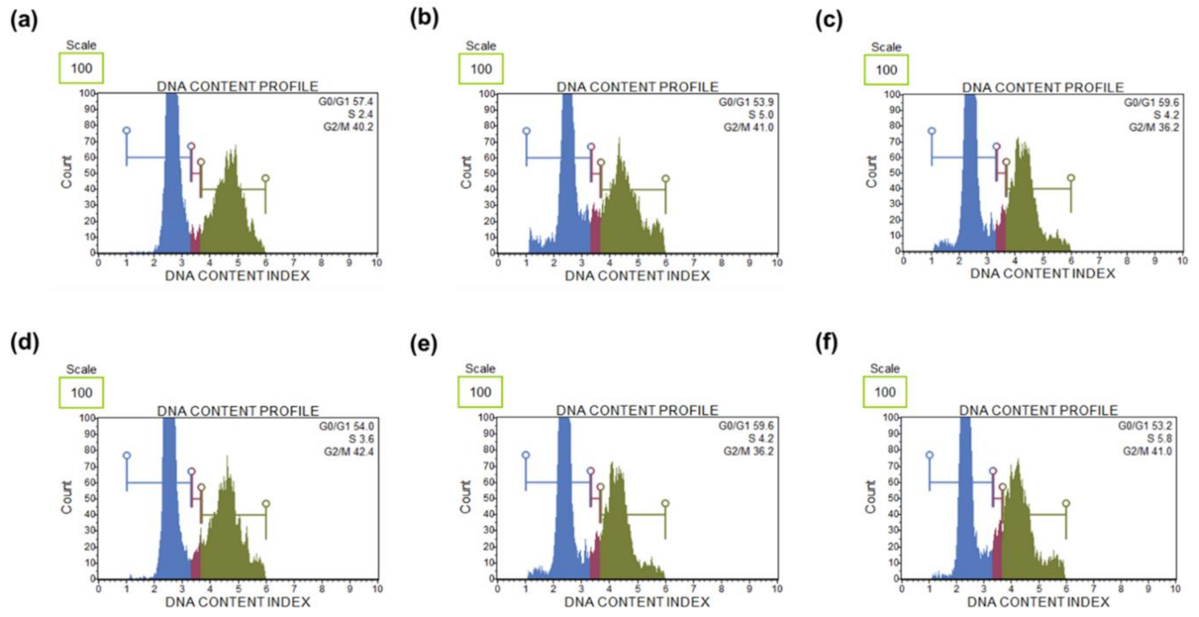

Figure S9. Cell cycle profiles of the A549 cells after 24 h treatment: (a) Control, (b) 0.25% DMSO, (c) 0.25% H_2O, (d) 50 µM curcumin, (e) 500 µg/mL ME and (f) 500 µg/mL WE. Muse® Cell Analyser was used to analyse the results.

Table S16: The cell cycle mean percentages and SEM of H1573 cells.

Treatment	Mean (%) ±SEM		
	**G0/G1**	**S**	**G2/M**

Control	36.100±2.473	10.533±2.062	55.017±1.006
0.25% DMSO	33.633±3.272	11.983±1.740	54.267±1.634
0.25% H_2O	32.850±1.636	10.667±0.545	56.400±1.222
50 µM Curcumin	42.250±4.883	19.550±5.614	38.067±1.204
125 µg/mL ME	59.100±1.264	8.700±1.111	32.150±1.578
125µg/mL WE	55.667±2.071	8.083±0.995	36.200±2.728

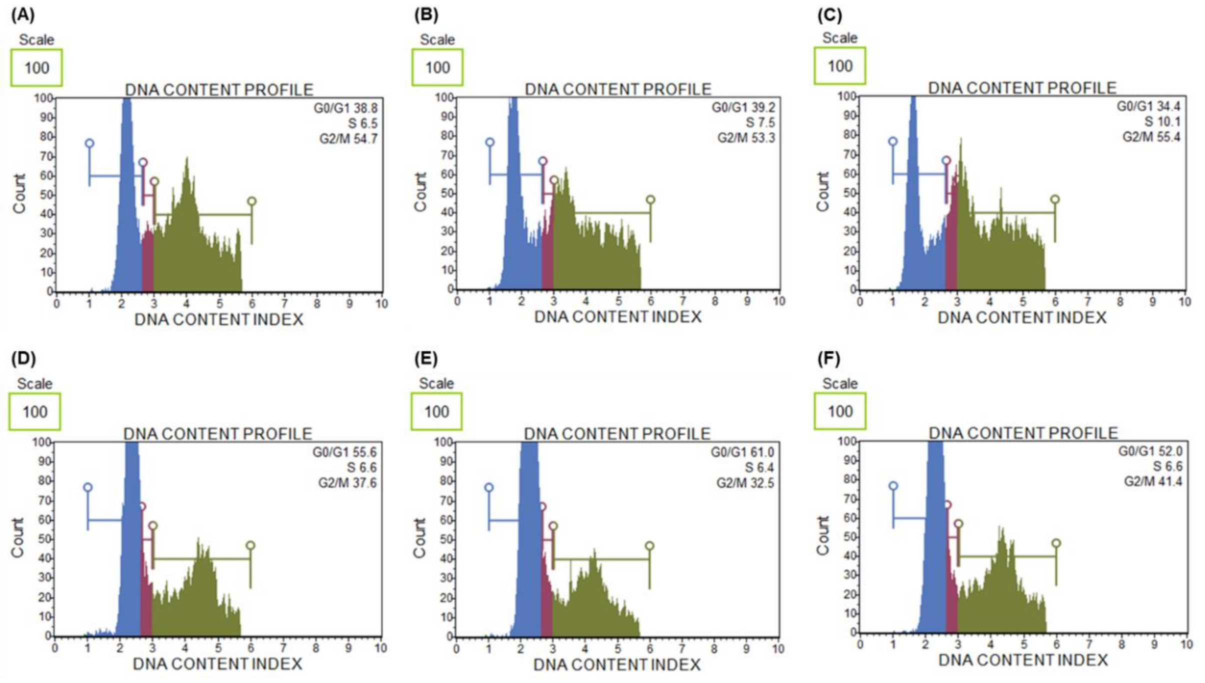

Figure S10. Cell cycle profiles of the H1573 cells after 24 h treatment: (a) Control, (b) 0.25% DMSO, (c) 0.25% H_2O, (d) 50 µM curcumin, (e) 125 µg/mL ME and (f) 125 µg/mL WE. Muse® Cell Analyser was used to analyse the results.

Table S17: The cell cycle mean percentages and SEM of H1437 cells.

Treatment	Mean (%) ±SEM		
	**G0/G1**	**S**	**G2/M**
Control	48.967±2.325	14.133±0.899	36.867±1.444
0.25% DMSO	50.567±1.466	15.500±0.900	33.900±0.608
0.25% H_2O	45.533±1.588	18.967±0.694	35.467±1.338
50 µM Curcumin	11.100±2.120	62.667±2.848	26.133±0.684

62.50 µg/mL ME	26.667±1.235	48.800±0.723	24.467±0.612
125 µg/mL WE	29.567±0.984	39.233±1.936	31.067±2.843

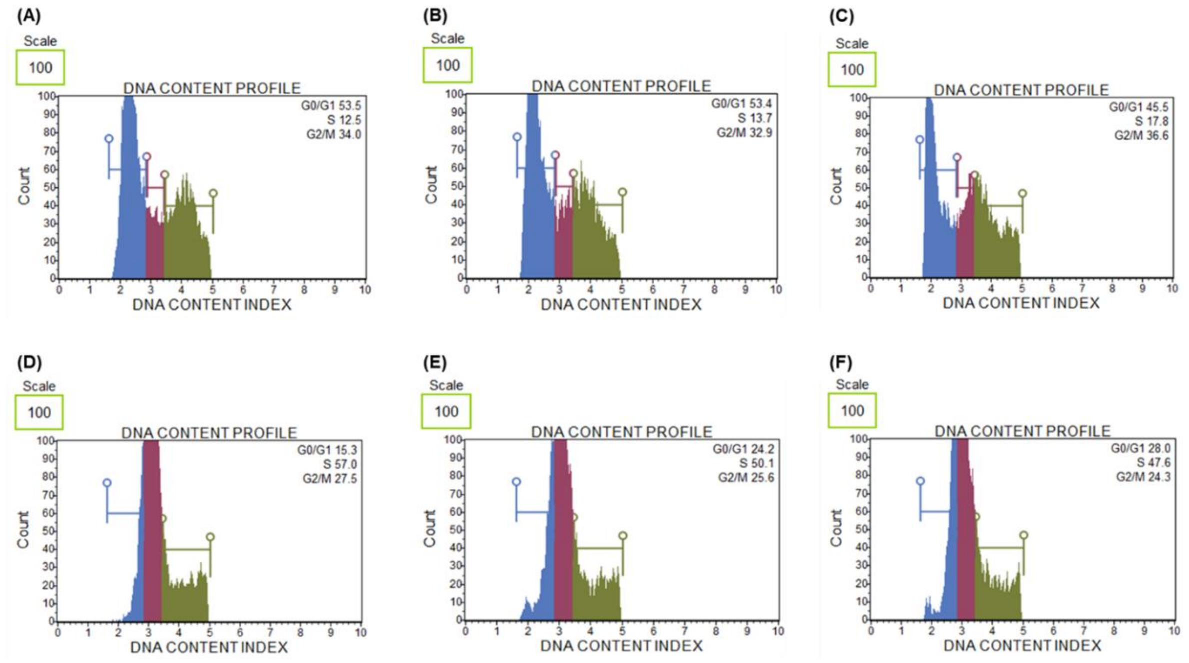

Figure S11. Cell cycle profiles of the H1437 cells after 24 h treatment: (a) Control, (b) 0.25% DMSO, (c) 0.25% H_2O, (d) 50 µM curcumin, (e) 125 µg/mL ME and (f) 125 µg/mL WE. Muse® Cell Analyser was used to analyse the results.

Cell cycle related genes

Table S18: The mean densities and SEM of cell cycle related genes in A549 cells.

**Treatment**	**Mean (INT/mm^2) ±SEM**				
	CLA1	***p21***	***CLB1***	***CDC2***	***GAPDH***
**0**		0.678±0.301	10.148±0.026	17.390±0.110	4.568±0.019
**0.25% DMSO**	0.000±0.000	0.958±0.060	10.227±0.047	15.655±0.124	4.683±0.050
**0.25% H_2O**	0.000±0.000	1.579± 0.073	12.045±0.047	16.010±0.128	4.630±0.047

**500 µg/mL ME**	0.000±0.000	2.074±0.097	7.849±0.010	15.709±0.087	4.509±0.051
**50 µM Curcumin**	0.000±0.000	1.924±0.019	10.388±0.011	16.333± 0.122	4.555±0.069
**HEK-293**	0.000±0.000	0.000±0.000	11.734±0.011	14.020±0.074	4.540±0.023
**Blank**	0.000±0.000	0.000±0.000	0.000±0.000	0.000±0.000	0.000±0.000

Table S19: The mean densities and SEM of cell cycle related genes in H1573 cells.

**Treatment**	**Mean (INT/mm^2) ±SEM**				
	CLA1	***p21***	***CLB1***	***CDC2***	***GAPDH***
**0**	3.691±0.028	3.606±0.031	17.737±0.055	15.100±0.137	12.231±0.1013
**0.25% DMSO**	3.935± 0.135	3.985± 0.046	17.989±0.089	15.051±0.139	12.576±0.069
**0.25% H_2O**	4.498±0.043	4.628±0.015	17.997± 0.103	14.920±0.142	12.767±0.061
**125 µg/mL ME**	0.207±0.012	6.724±0.033	17.350±0.073	14.518±0.075	13.025± 0.002
**125 µg/mL WE**	0.199±0.016	1.454±0.016	0.000±0.000	0.756±0.0324	12.824±0.064
**50 µM Curcumin**	1.687±0.005	10.752±0.043	18.136±0.097	15.249±0.1619	13.041±0.124
**HEK-293**	0.000±0.000	0.2617±.038	17.438±0.115	14.550±0.150	12.753±0.069
**Blank**	0.000±0.000	0.000±0.000	0.000±0.000	0.000±0.000	0.000±0.000

Table S20: The mean densities and SEM of cell cycle related genes in H1437 cells.

Treatment	Mean (INT/mm^2) ±SEM				
	CLA1	*p21*	*CLB1*	*CDC2*	*GAPDH*
**0**	0.000±0.000	0.492±0.046	10.747±0.052	10.674±0.034	11.050±0.100
**0.25% DMSO**	0.000±0.000	0.929±0.023	11.066± 0.070	10.867±0.052	11.413±0.098
**0.25% H_2O**	0.000±0.000	1.064±0.018	11.197±0.055	10.343±0.038	11.449±0.096
**62.50 μg/mL ME**	0.000±0.000	4.808±0.014	11.074±0.071	11.654±0.054	11.779±0.101
**125 μg/mL WE**	0.000±0.000	11.944±0.172	11.345±0.071	11.605±0.037	11.599±0.106
**50 μM Curcumin**	8.428±0.2157	12.355±0.169	11.040±0.061	11.586±0.045	11.686±0.103
**HEK-293**	0.000±0.000	0.000±0.000	10.978±0.054	10.936±0.065	11.085±0.066
**Blank**	0.000±0.000	0.000±0.000	0.000±0.000	0.000±0.000	0.000±0.000

STAT genes

Table S21: The mean densities and SEM of STAT genes in A549 cells.

Treatment	Mean (INT/mm^2) ±SEM				
	STAT1	*STAT3*	*STAT5A*	*STAT5B*	*GAPDH*
**0**	14.221±0.088	2.099±0.058	0.107±0.015	9.593±0.099	4.568±0.019
**0.25% DMSO**	13.857±0.084	1.834±0.031	0.126±0.014	9.096±0.097	4.683±0.050
**0.25% H_2O**	13.430±0.112	1.629±0.038	0.071±0.005	7.969±0.079	4.630±0.047

**500 µg/mL ME**	13.189±0.102	1.752±0.014	0.267±0.024	6.558±0.085	4.509±0.051
**50 µM Curcumin**	13.582±0.104	2.067±0.017	0.335± 0.028	3.889±0.051	4.555±0.069
**HEK-293**	3.236±0.063	3.939±0.026	0.072±0.023	3.889±0.051	4.540±0.023
**Blank**	0.000±0.000	0.000±0.000	0.000±0.000	0.000±0.000	0.000±0.000

Table S22: The mean densities and SEM of STAT genes in H1573 cells.

**Treatment**	**Mean (INT/mm^2) ±SEM**				
	STAT1	***STAT3***	***STAT5A***	***STAT5B***	***GAPDH***
**0**	10.205±0.031	15.119±0.120	2.491±0.146	6.901±0.065	12.231±0.101 3
**0.25% DMSO**	10.785±0.027	14.154±0.078	2.613±0.159	7.508±0.089	12.576±0.069
**0.25% H_2O**	10.313±0.017	12.720±0.194	2.431±0.146	7.245±0.080	12.767±0.061
**125 µg/mL ME**	9.208±0.540	14.235±0.186	0.359±0.090	4.633±0.028	13.025± 0.002
**125 µg/mL WE**	2.287±0.103	0.7453±0.330	0.854±0.114	6.152±0.051	12.824±0.064
**50 µM Curcumin**	10.299±0.017	14.456±0.079	0.247±0.052	1.974±0.015	13.041±0.124
**HEK-293**	2.341±0.017	0.474±0.050	0.000±0.000	2.861±0.008	12.753±0.069
**Blank**	0.000±0.000	0.000±0.000	0.000±0.000	0.000±0.000	0.000±0.000

Table S23: The mean densities and SEM of STAT genes in H1437 cells.

Treatment	Mean (INT/mm^2) ±SEM				
	STAT1	***STAT3***	***STAT5A***	***STAT5B***	***GAPDH***
**0**	12.035±0.048	15.960±0.936	0.476±0.019	2.517±0.018	11.050±0.100
**0.25% DMSO**	9.996±0.050	13.676±0.198	0.4467±0.026	2.701±0.019	11.413±0.098
**0.25% H_2O**	9.213±0.072	10.137±0.164	0.5387±0.026	2.757±0.020	11.449±0.096
**62.50 µg/mL ME**	13.642±0.033	19.402±0.170	1.621±0.021	2.527± 0.010	11.779±0.101
**125 µg/mL WE**	14.253±0.068	21.817±0.075	10.356±0.056	7.259±0.005	11.599±0.106
**50 µM Curcumin**	14.473±0.061	23.985±0.069	8.711±0.055	6.757±0.0183	11.686±0.103
**HEK-293**	2.614±0.038	1.088±0.084	0.398±0.072	5.312±0.017	11.085±0.066
**Blank**	0.000±0.000	0.000±0.000	0.000±0.000	0.000±0.000	0.000±0.000

The fractions chromatograms

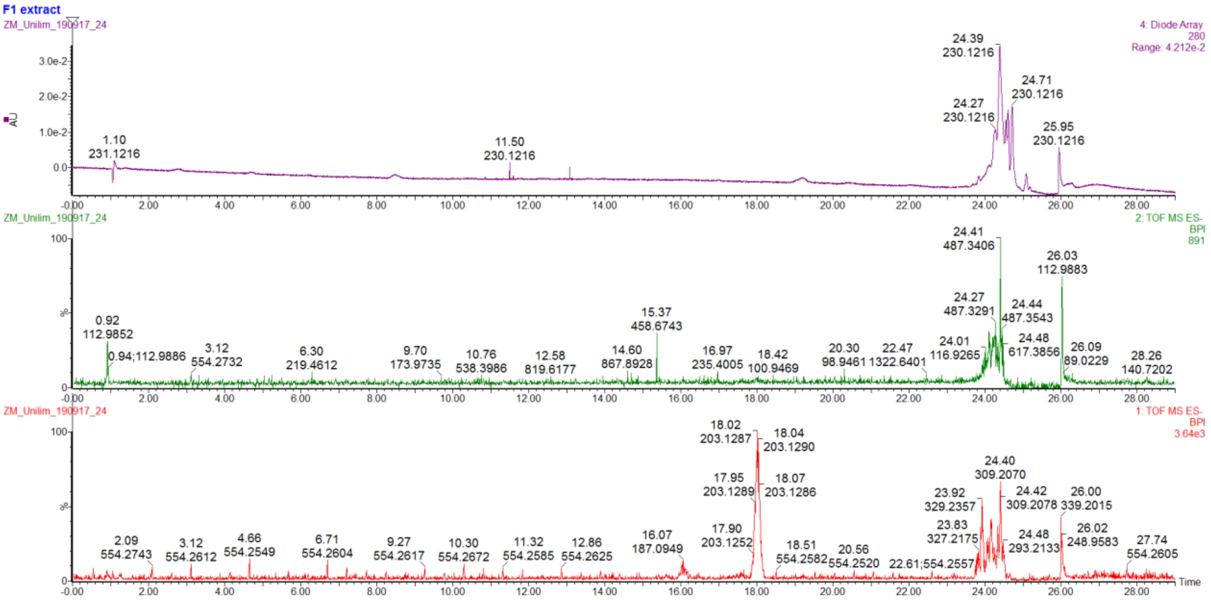

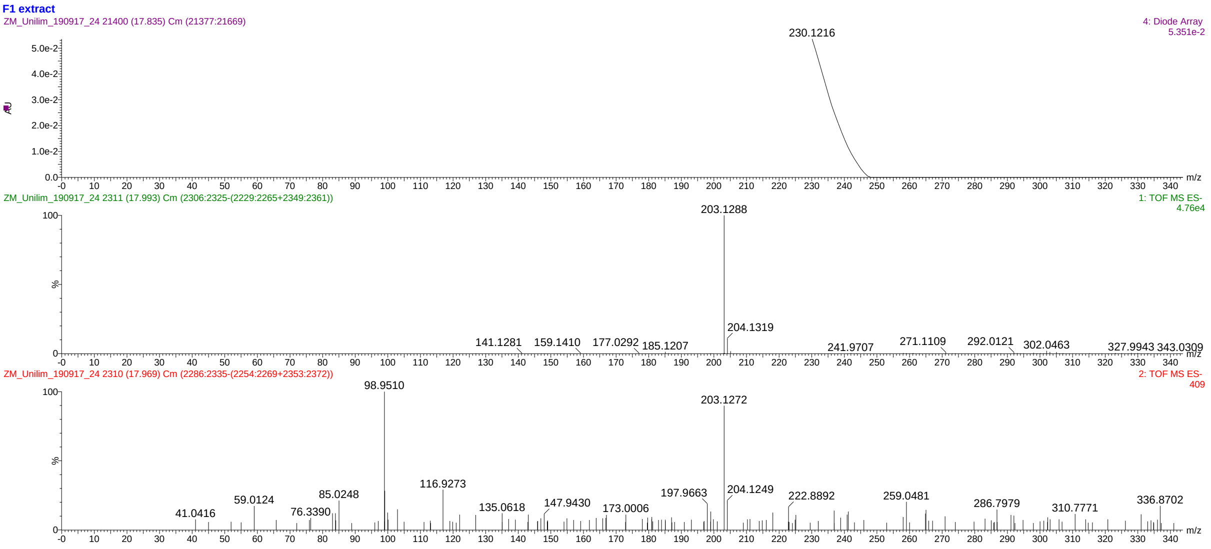

Peak with m/z 203.1272 at 18.02 minutes. No UV above 230nm and low DBE (double bond equivalents) probably a straight-chain fatty acid or surfactant

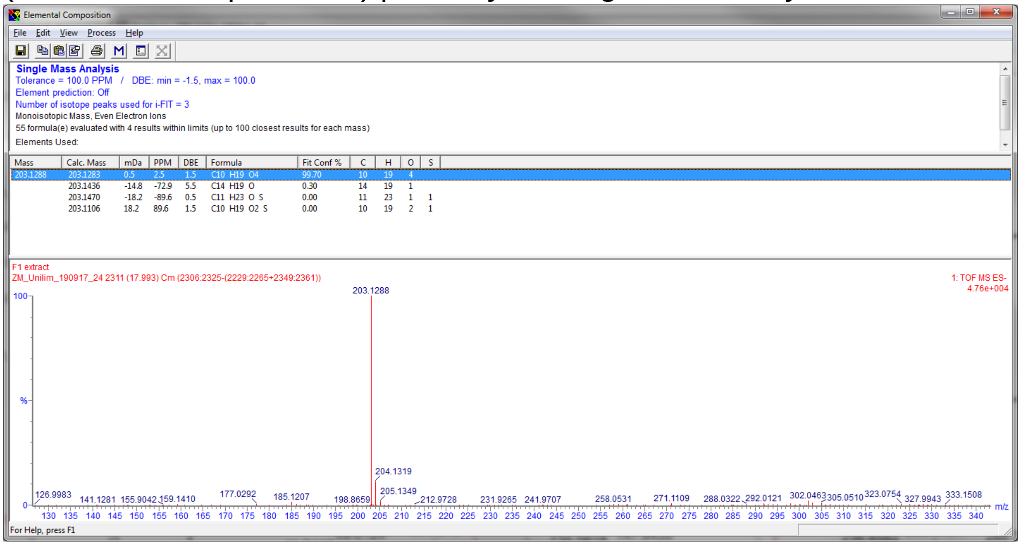

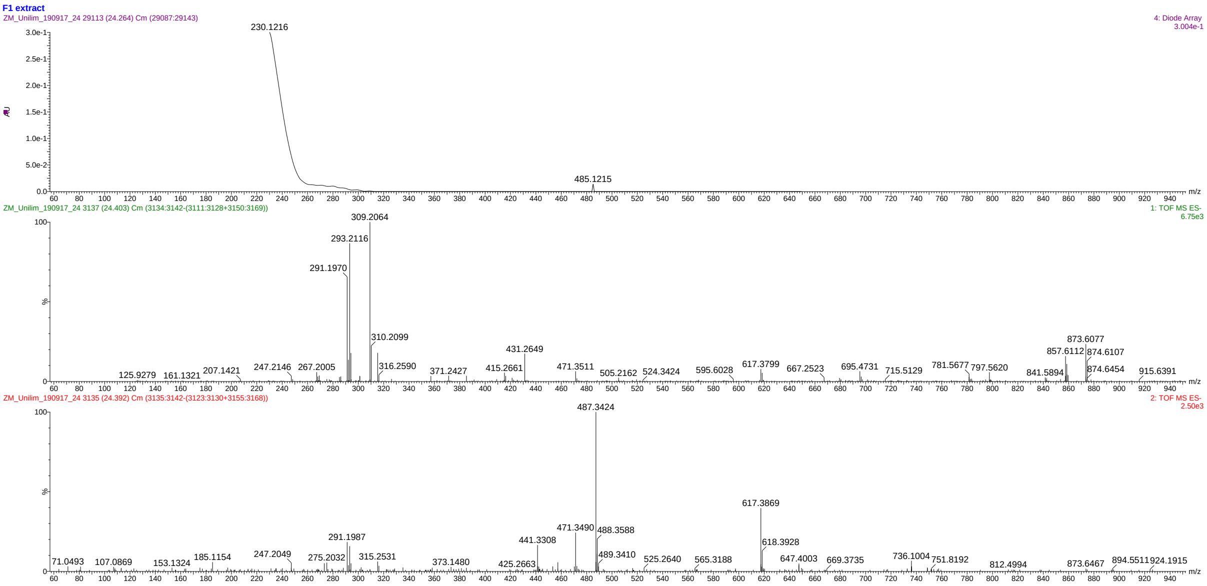

Peak at 24.2 minutes in F1 extract : Unknown

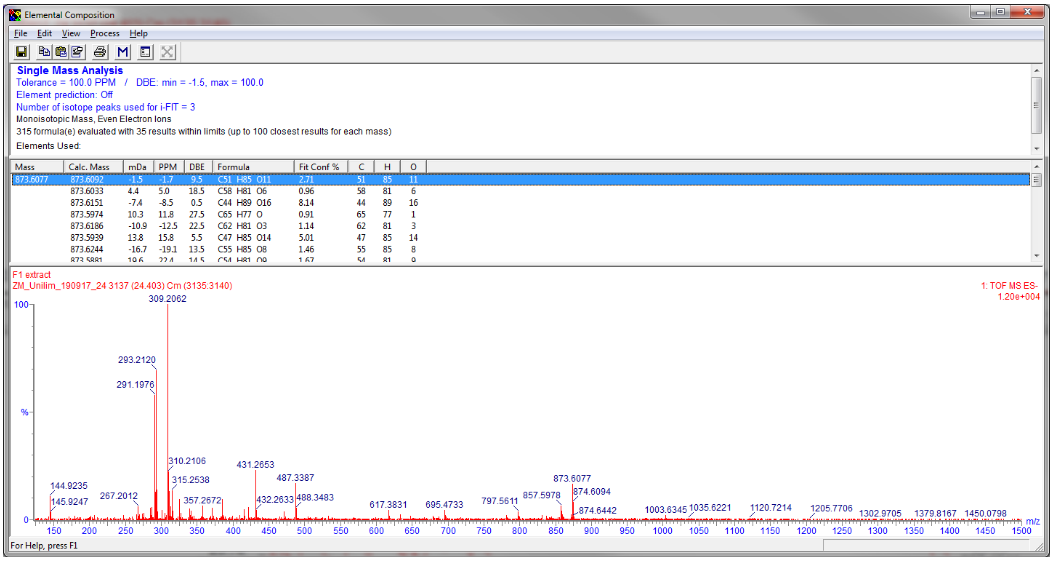
Elemental Composition

File Edit View Process Help

Single Mass Analysis
Tolerance = 100.0 PPM / DBE: min = -1.5, max = 100.0
Element prediction: Off
Number of isotope peaks used for i-FIT = 3
Monoisotopic Mass, Even Electron Ions
315 formula(e) evaluated with 35 results within limits (up to 100 closest results for each mass)
Elements Used:

Mass	Calc. Mass	mDa	PPM	DBE	Formula	Fit Conf %	C	H	O
873.6077	873.6092	-1.5	-1.7	9.5	C51 H85 O11	2.71	51	85	11
	873.6033	4.4	5.0	18.5	C58 H81 O6	0.96	58	81	6
	873.6151	-7.4	-8.5	0.5	C44 H89 O16	8.14	44	89	16
	873.5974	10.3	11.8	27.5	C65 H77 O	0.91	65	77	1
	873.6186	-10.9	-12.5	22.5	C62 H81 O3	1.14	62	81	3
	873.5939	13.8	15.8	5.5	C47 H85 O14	5.01	47	85	14
	873.6244	-16.7	-19.1	13.5	C55 H85 O8	1.46	55	85	8
	873.5881	19.6	22.4	14.5	C54 H81 O9	1.67	54	81	9

F1 extract
ZM_Unilim_190917_24 3137 (24.403) Cm (3135:3140)
1: TOF MS ES-
1.20e+004

For Help, press F1

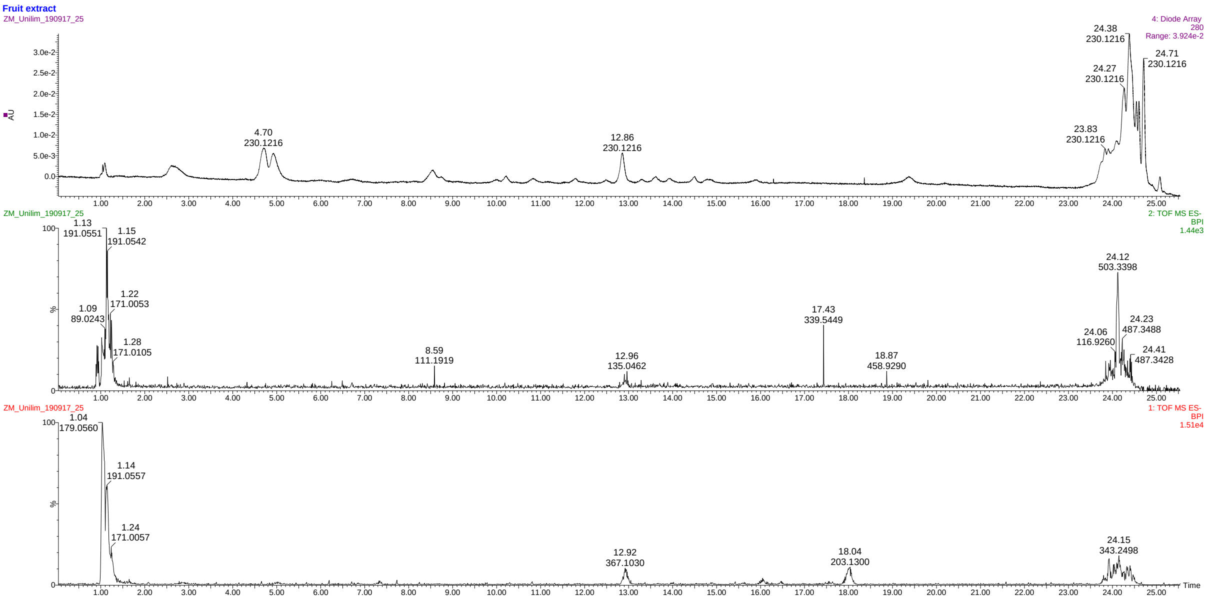

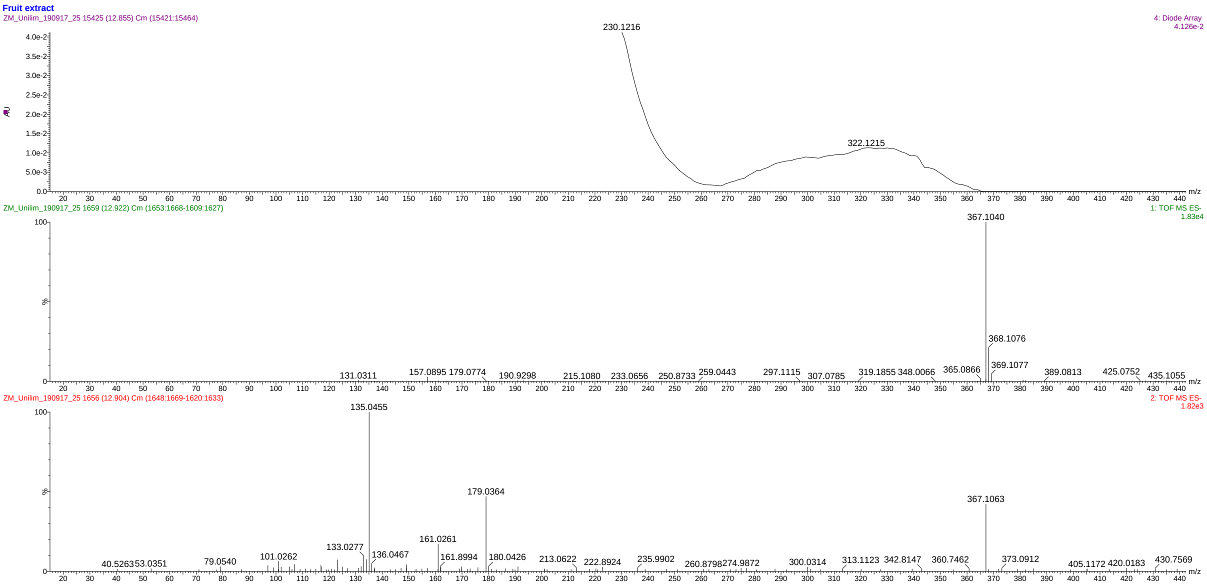

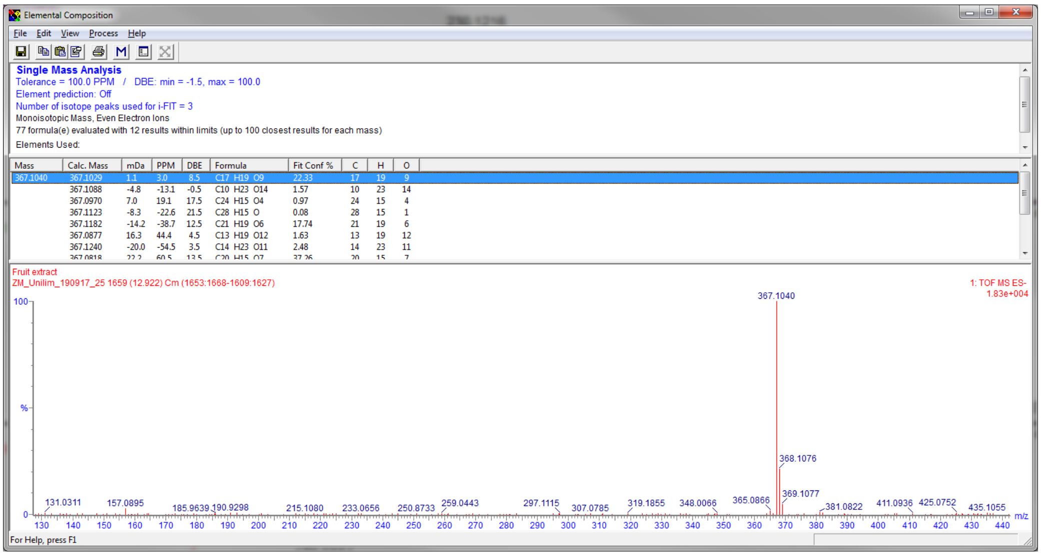

Mass	Calc. Mass	mDa	PPM	DBE	Formula	Fit Conf %	C	H	O
367.1040	367.1029	1.1	3.0	8.5	C17 H19 O9	22.33	17	19	9
	367.1088	-4.8	-13.1	-0.5	C10 H23 O14	1.57	10	23	14
	367.0970	7.0	19.1	17.5	C24 H15 O4	0.97	24	15	4
	367.1123	-8.3	-22.6	21.5	C28 H15 O	0.08	28	15	1
	367.1182	-14.2	-38.7	12.5	C21 H19 O6	17.74	21	19	6
	367.0877	16.3	44.4	4.5	C13 H19 O12	1.63	13	19	12
	367.1240	-20.0	-54.5	3.5	C14 H23 O11	2.48	14	23	11

Feruloyl quinic acid at 12.8 minutes

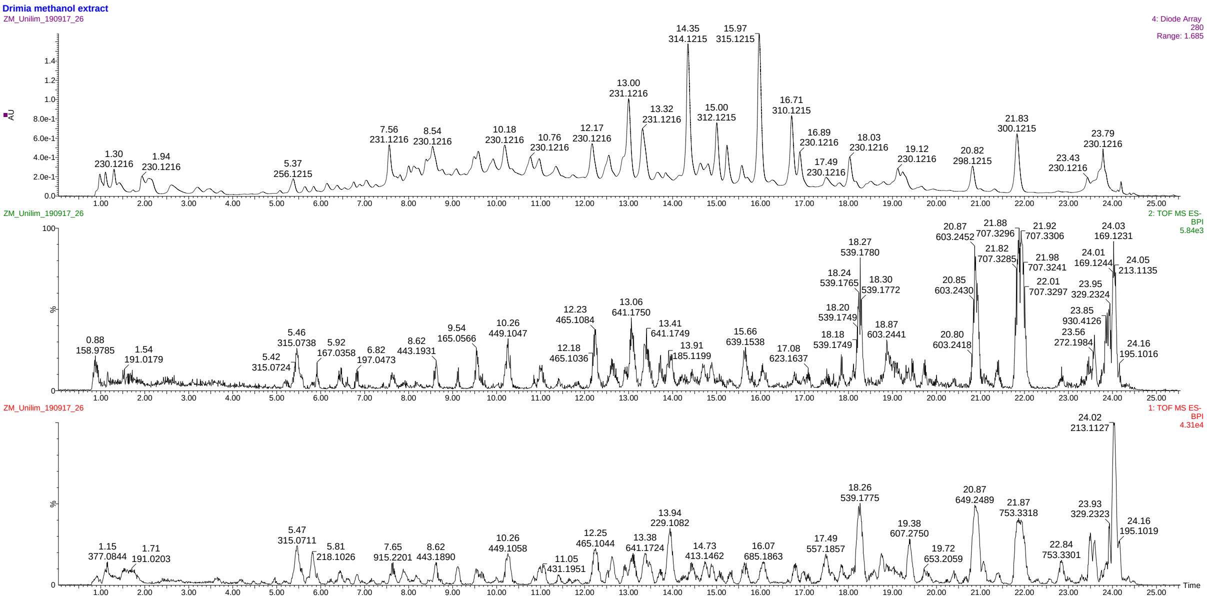

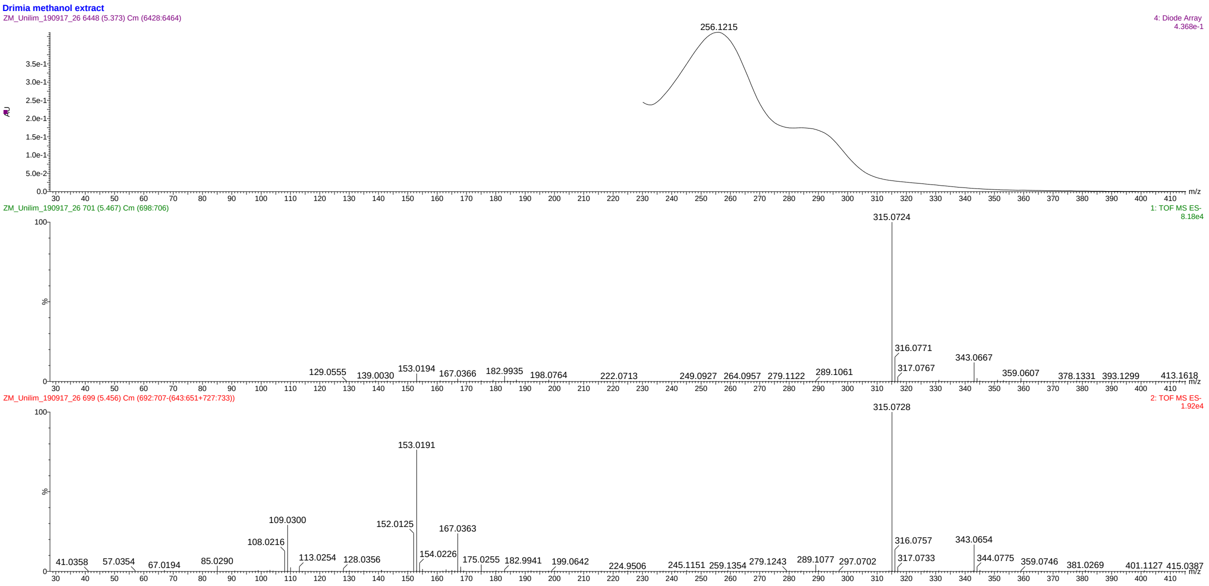

Protocatechuic acid O-glucoside at 5.47 minutes

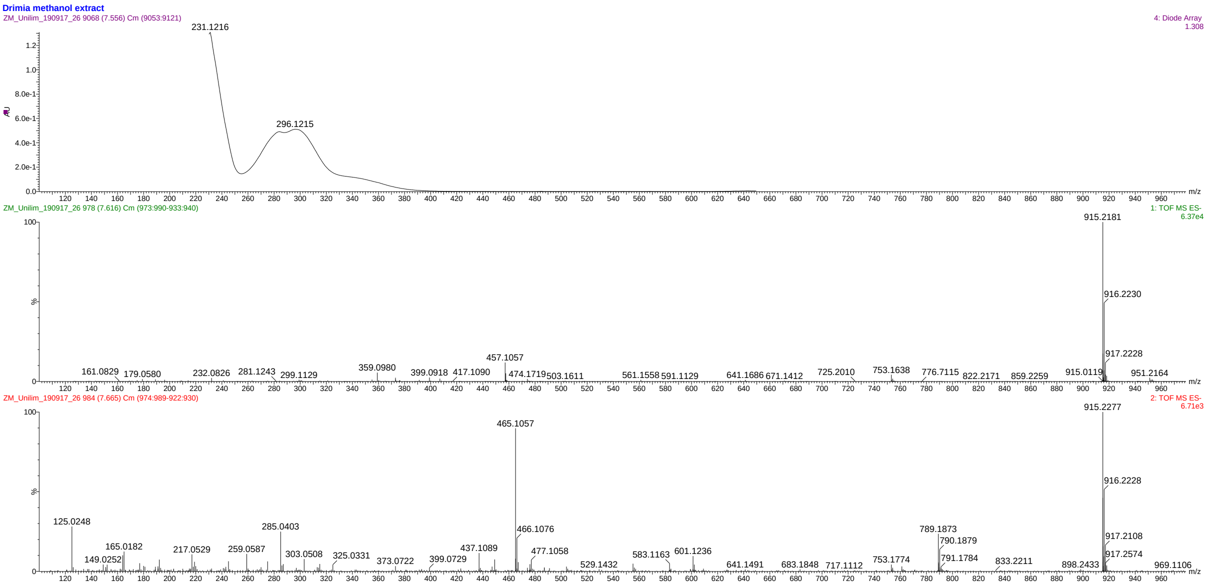

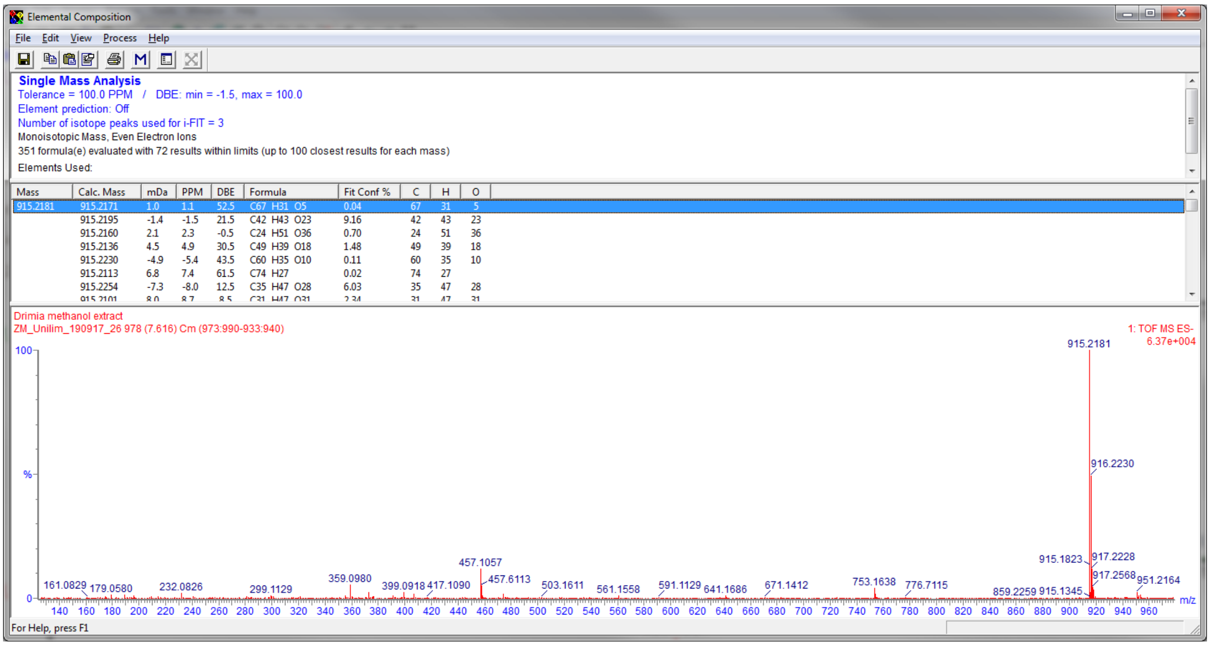

Mass	Calc. Mass	mDa	PPM	DBE	Formula	Fit Conf %	C	H	O
915.2181	915.2171	1.0	1.1	52.5	C67 H31 O5	0.04	67	31	5
	915.2195	-1.4	-1.5	21.5	C42 H43 O23	9.16	42	43	23
	915.2160	2.1	2.3	-0.5	C24 H51 O36	0.70	24	51	36
	915.2136	4.5	4.9	30.5	C49 H39 O18	1.48	49	39	18
	915.2230	-4.9	-5.4	43.5	C60 H35 O10	0.11	60	35	10
	915.2113	6.8	7.4	61.5	C74 H27	0.02	74	27	
	915.2254	-7.3	-8.0	12.5	C35 H47 O28	6.03	35	47	28
	915.2101	8.0	8.7	8.5	C31 H47 O31	2.34	31	47	31

Unknown at 7.62 minutes

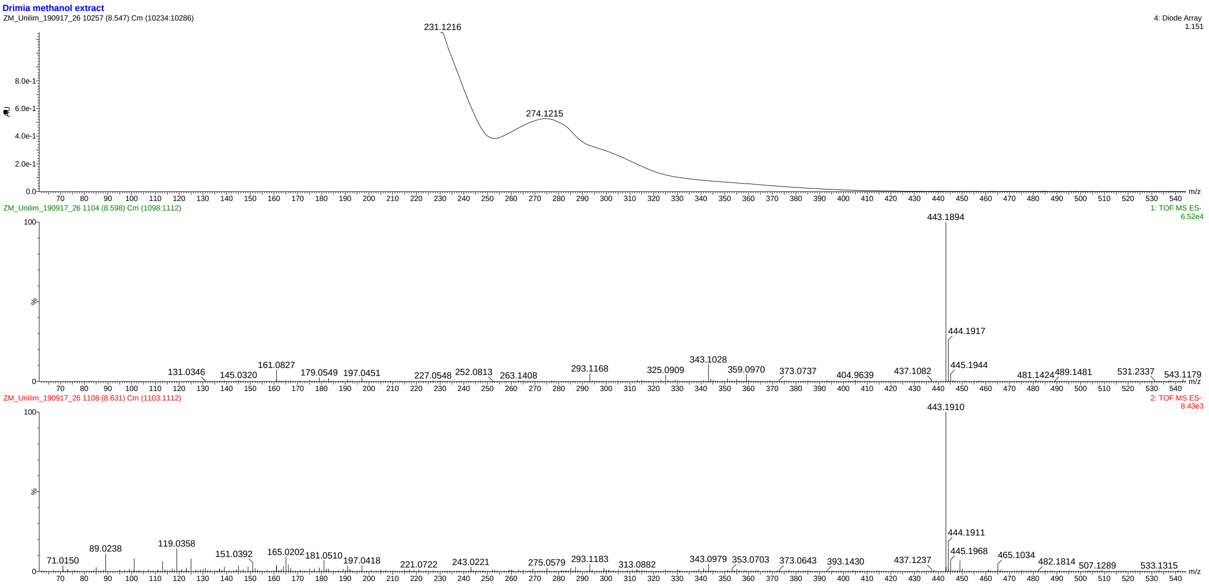
Drimia methanol extract
ZM_Unilim_190917_26 10257 (8.547) Cm (10234:10286)
4: Diode Array
1.151
231.1216
274.1215
ZM_Unilim_190917_26 1104 (8.598) Cm (1098:1112)
1: TOF MS ES-
6.52e4
443.1894
444.1917
445.1944
ZM_Unilim_190917_26 1108 (8.631) Cm (1103:1112)
2: TOF MS ES-
8.43e3
443.1910
444.1911
445.1968
m/z

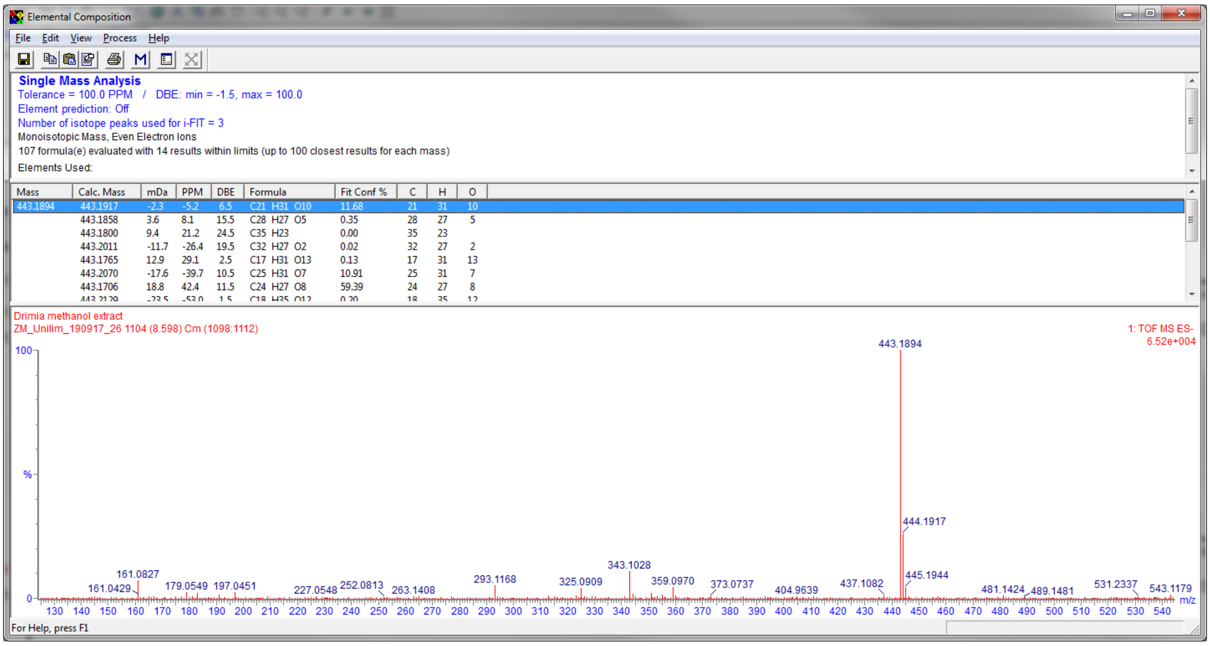
Elemental Composition
File Edit View Process Help
Single Mass Analysis
Tolerance = 100.0 PPM / DBE: min = -1.5, max = 100.0
Element prediction: Off
Number of isotope peaks used for i-FIT = 3
Monoisotopic Mass, Even Electron Ions
107 formula(e) evaluated with 14 results within limits (up to 100 closest results for each mass)
Elements Used:
Mass | Calc. Mass | mDa | PPM | DBE | Formula | Fit Conf % | C | H | O
443.1894 | 443.1917 | -2.3 | -5.2 | 6.5 | C21 H31 O10 | 11.68 | 21 | 31 | 10
443.1858 | 3.6 | 8.1 | 15.5 | C28 H27 O5 | 0.35 | 28 | 27 | 5
443.1800 | 9.4 | 21.2 | 24.5 | C35 H23 | 0.00 | 35 | 23
443.2011 | -11.7 | -26.4 | 19.5 | C32 H27 O2 | 0.02 | 32 | 27 | 2
443.1765 | 12.9 | 29.1 | 2.5 | C17 H31 O13 | 0.13 | 17 | 31 | 13
443.2070 | -17.6 | -39.7 | 10.5 | C25 H31 O7 | 10.91 | 25 | 31 | 7
443.1706 | 18.8 | 42.4 | 11.5 | C24 H27 O8 | 59.39 | 24 | 27 | 8
Drimia methanol extract
ZM_Unilim_190917_26 1104 (8.598) Cm (1098:1112)
1: TOF MS ES-
6.52e+004
443.1894
444.1917
445.1944
m/z
For Help, press F1

Unknown

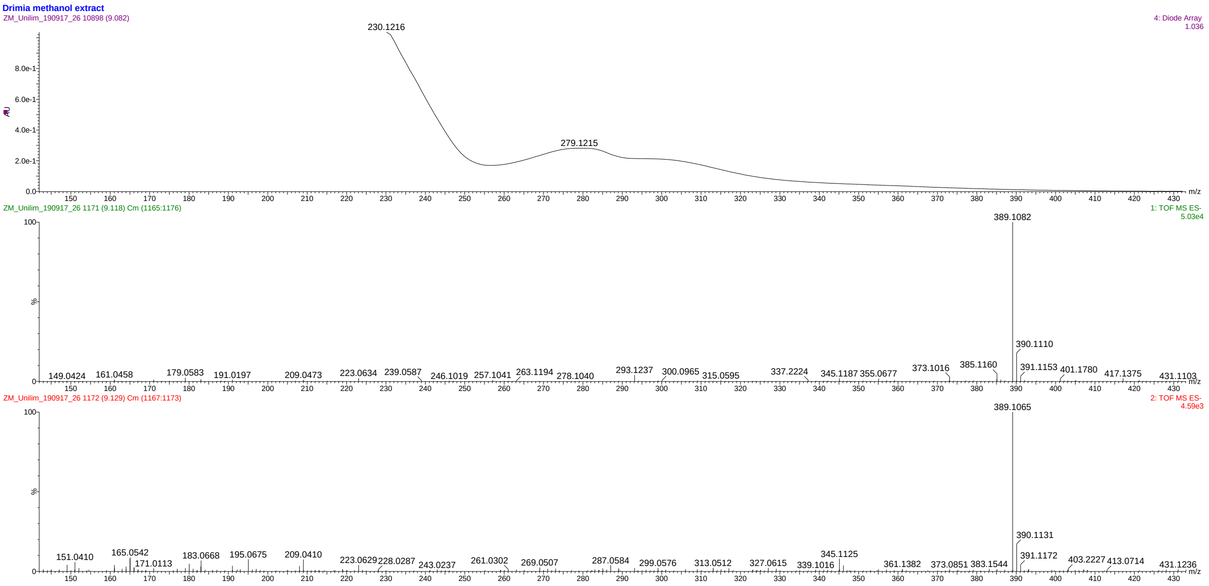

Elemental Composition

File Edit View Process Help

Single Mass Analysis

Tolerance = 100.0 PPM / DBE: min = -1.5, max = 100.0

Element prediction: Off

Number of isotope peaks used for i-FIT = 3

Monoisotopic Mass, Even Electron Ions

83 formula(e) evaluated with 14 results within limits (up to 100 closest results for each mass)

Elements Used:

Mass	Calc. Mass	mDa	PPM	DBE	Formula	Fit Conf %	C	H	O
389.1082	389.1084	-0.2	-0.5	6.5	$C_{16}H_{21}O_{11}$	74.40	16	21	11
	389.1025	5.7	14.6	15.5	$C_{23}H_{17}O_6$	0.31	23	17	6
	389.1178	-9.6	-24.7	19.5	$C_{27}H_{17}O_3$	0.13	27	17	3
	389.0966	11.6	29.8	24.5	$C_{30}H_{13}O$	0.10	30	13	1
	389.0931	15.1	38.8	2.5	$C_{12}H_{21}O_{14}$	0.20	12	21	14
	389.1236	-15.4	-39.6	10.5	$C_{20}H_{21}O_8$	0.80	20	21	8
	389.0873	20.9	53.7	11.5	$C_{19}H_{17}O_9$	1.62	19	17	9
	389.1295	-21.3	-54.7	1.5	$C_{13}H_{25}O_{13}$	0.61	13	25	13

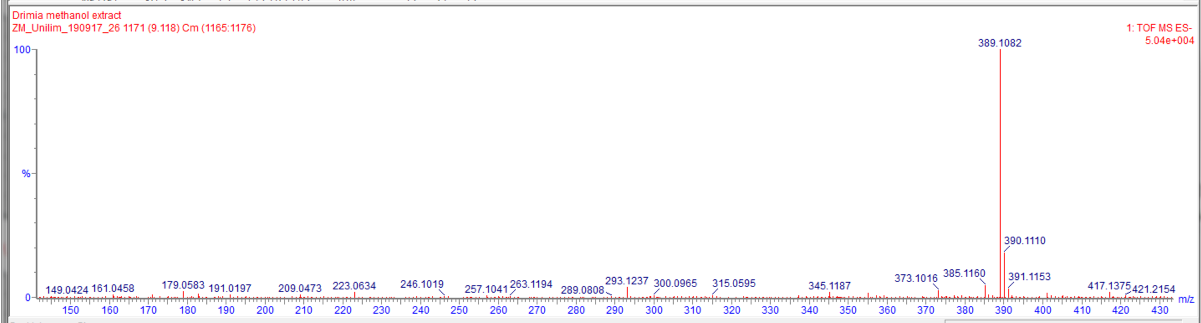

For Help, press F1

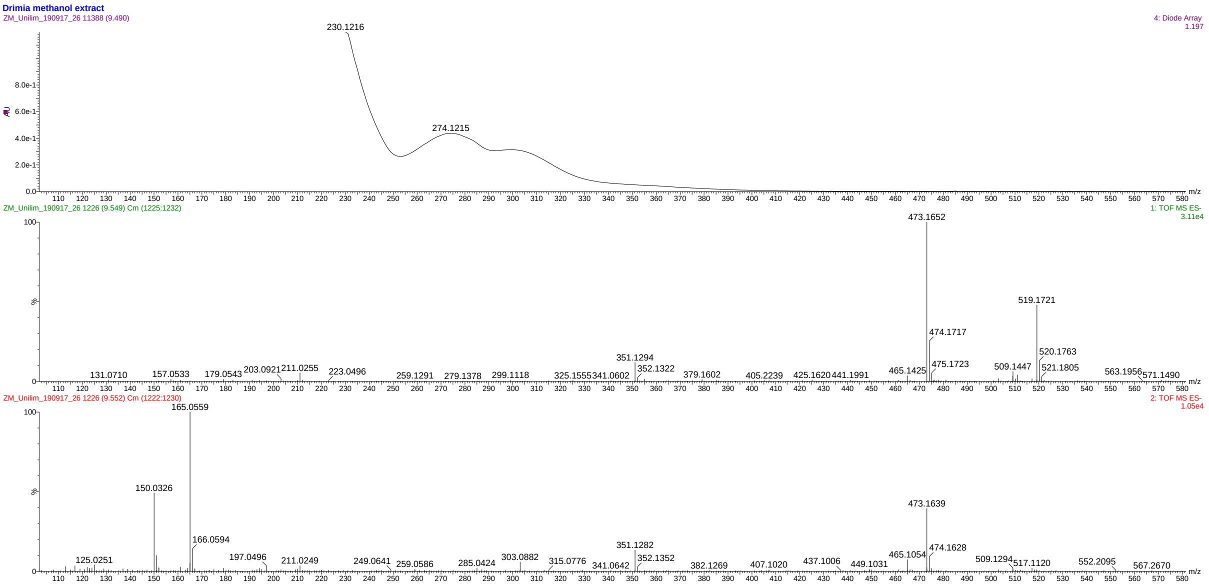

Elemental Composition

File Edit View Process Help

Single Mass Analysis
Tolerance = 100.0 PPM / DBE: min = -1.5, max = 100.0
Element prediction: Off
Number of isotope peaks used for i-FIT = 3
Monoisotopic Mass, Even Electron Ions
140 formula(e) evaluated with 22 results within limits (up to 100 closest results for each mass)
Elements Used:

Mass	Calc. Mass	mDa	PPM	DBE	Formula	Fit Conf %	C	H	O
519.1721	519.1714	0.7	1.3	7.5	C22 H31 O14	1.00	22	31	14
	519.1749	-2.8	-5.4	29.5	C40 H23 O	0.01	40	23	1
	519.1773	-5.2	-10.0	-1.5	C15 H35 O19	0.00	15	35	19
	519.1655	6.6	12.7	16.5	C29 H27 O9	0.32	29	27	9
	519.1808	-8.7	-16.8	20.5	C33 H27 O6	0.04	33	27	6
	519.1596	12.5	24.1	25.5	C36 H23 O4	0.02	36	23	4
	519.1866	-14.5	-27.9	11.5	C26 H31 O11	10.20	26	31	11
	519.1561	16.0	30.8	3.5	C18 H31 O17	0.01	18	31	17

Drimia methanol extract
ZM_Unilim_190917_26 1226 (9.549) Cm (1225:1232)

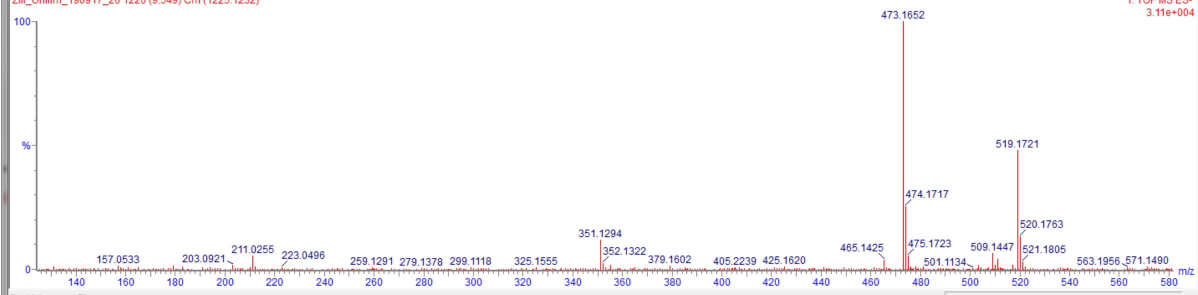

For Help, press F1

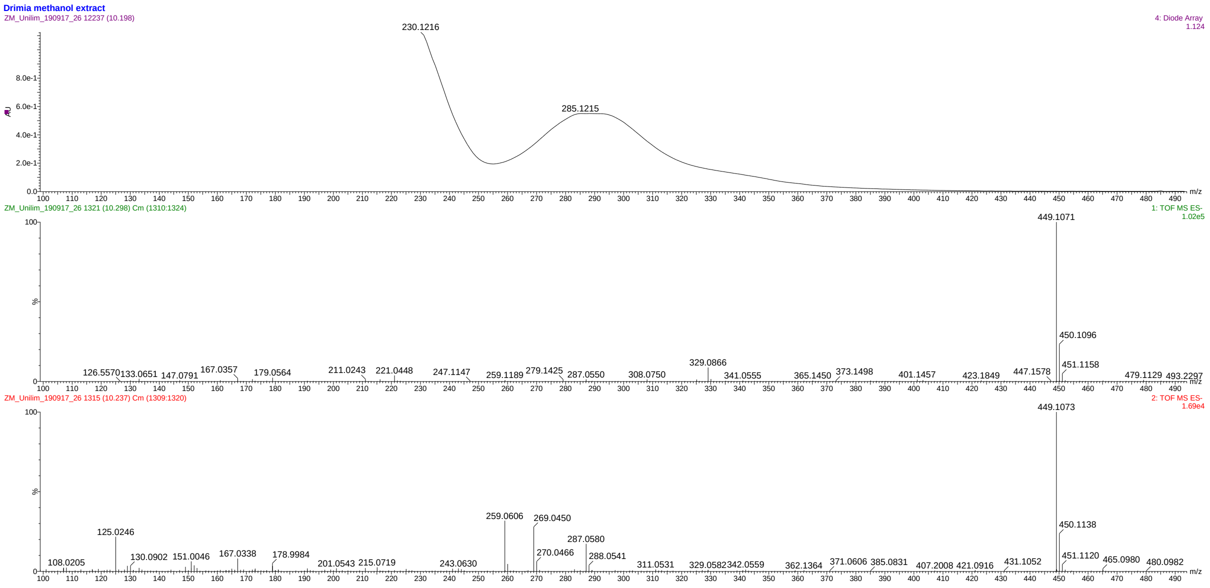

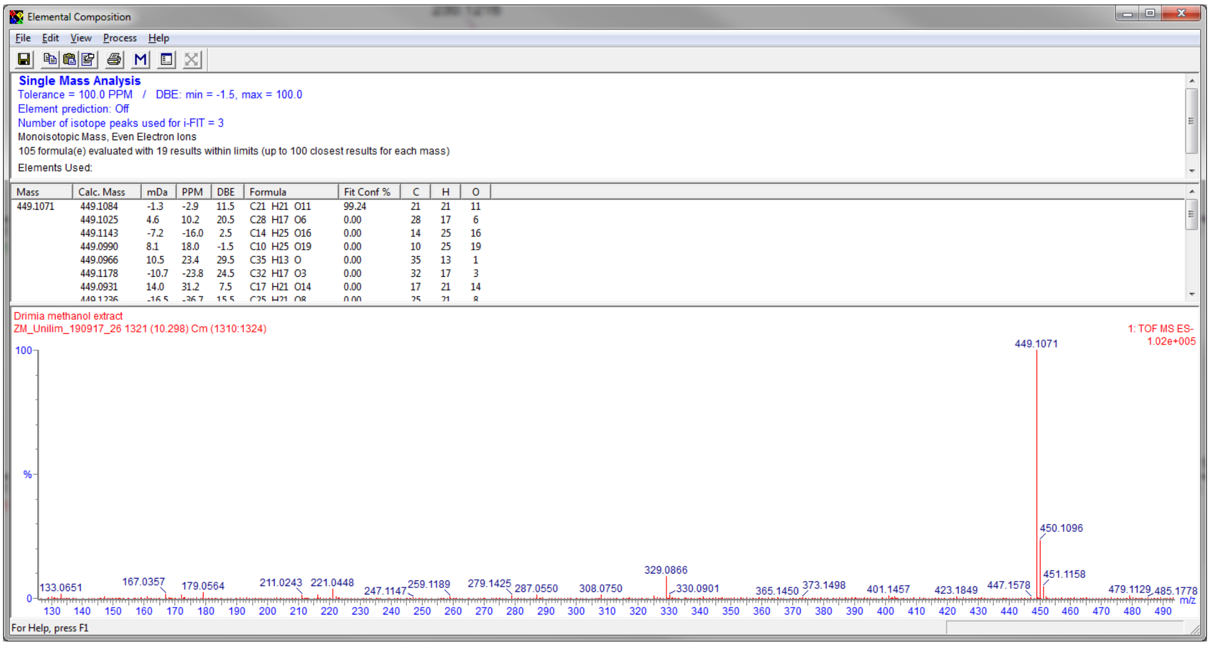

Elemental Composition

File Edit View Process Help

Single Mass Analysis

Tolerance = 100.0 PPM / DBE: min = -1.5, max = 100.0

Element prediction: Off

Number of isotope peaks used for i-FIT = 3

Monoisotopic Mass, Even Electron Ions

105 formula(e) evaluated with 19 results within limits (up to 100 closest results for each mass)

Elements Used:

Mass	Calc. Mass	mDa	PPM	DBE	Formula	Fit Conf %	C	H	O
449.1071	449.1084	-1.3	-2.9	11.5	C21 H21 O11	99.24	21	21	11
	449.1025	4.6	10.2	20.5	C28 H17 O6	0.00	28	17	6
	449.1143	-7.2	-16.0	2.5	C14 H25 O16	0.00	14	25	16
	449.0990	8.1	18.0	-1.5	C10 H25 O19	0.00	10	25	19
	449.0966	10.5	23.4	29.5	C35 H13 O	0.00	35	13	1
	449.1178	-10.7	-23.8	24.5	C32 H17 O3	0.00	32	17	3
	449.0931	14.0	31.2	7.5	C17 H21 O14	0.00	17	21	14
	449.1236	-16.5	-36.7	15.5	C25 H21 O8	0.00	25	21	8

Drimia methanol extract

ZM_Unilim_190917_26 1321 (10.298) Cm (1310:1324)

1: TOF MS ES-
1.02e+005

449.1071

For Help, press F1

Eriodictyol 7-O-glucoside

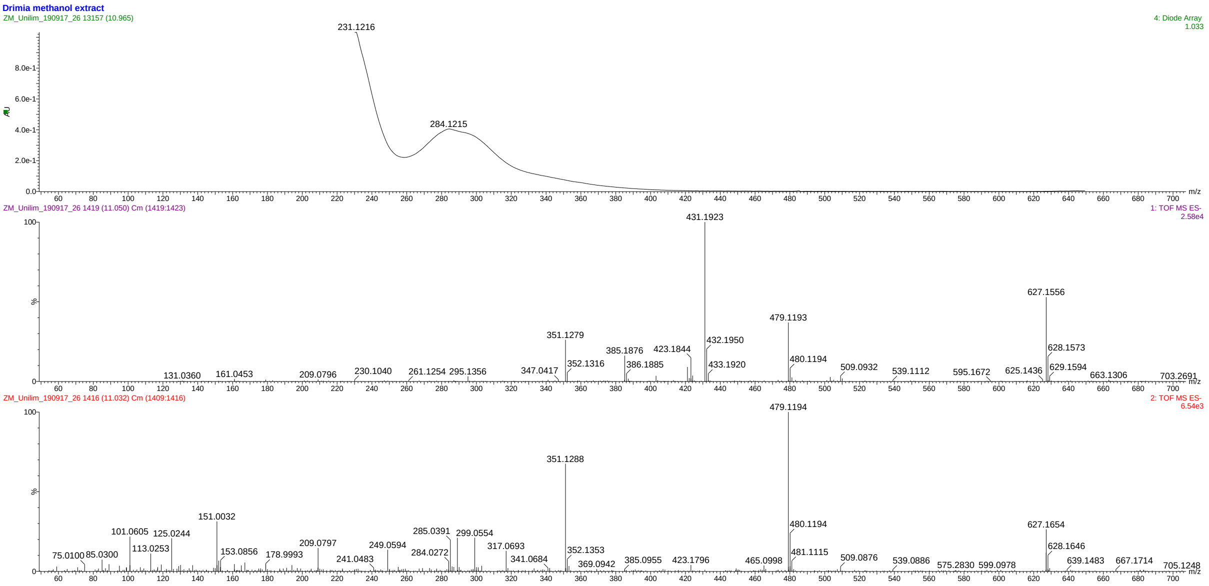

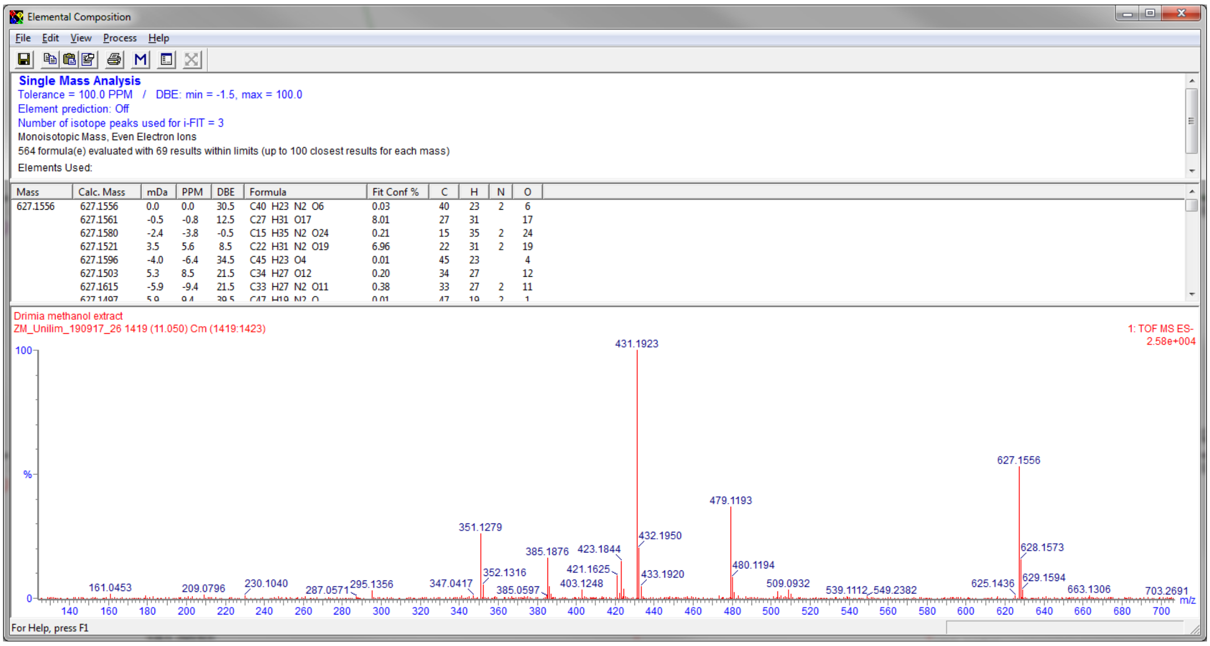

Mass	Calc. Mass	mDa	PPM	DBE	Formula	Fit Conf %	C	H	N	O
627.1556	627.1556	0.0	0.0	30.5	C40 H23 N2 O6	0.03	40	23	2	6
	627.1561	-0.5	-0.8	12.5	C27 H31 O17	8.01	27	31		17
	627.1580	-2.4	-3.8	-0.5	C15 H35 N2 O24	0.21	15	35	2	24
	627.1521	3.5	5.6	8.5	C22 H31 N2 O19	6.96	22	31	2	19
	627.1596	-4.0	-6.4	34.5	C45 H23 O4	0.01	45	23		4
	627.1503	5.3	8.5	21.5	C34 H27 O12	0.20	34	27		12
	627.1615	-5.9	-9.4	21.5	C33 H27 N2 O11	0.38	33	27	2	11
	627.1497	5.9	9.4	39.5	C47 H19 N2 O	0.01	47	19	2	1

Unknown alkaloid at 11.05 minutes

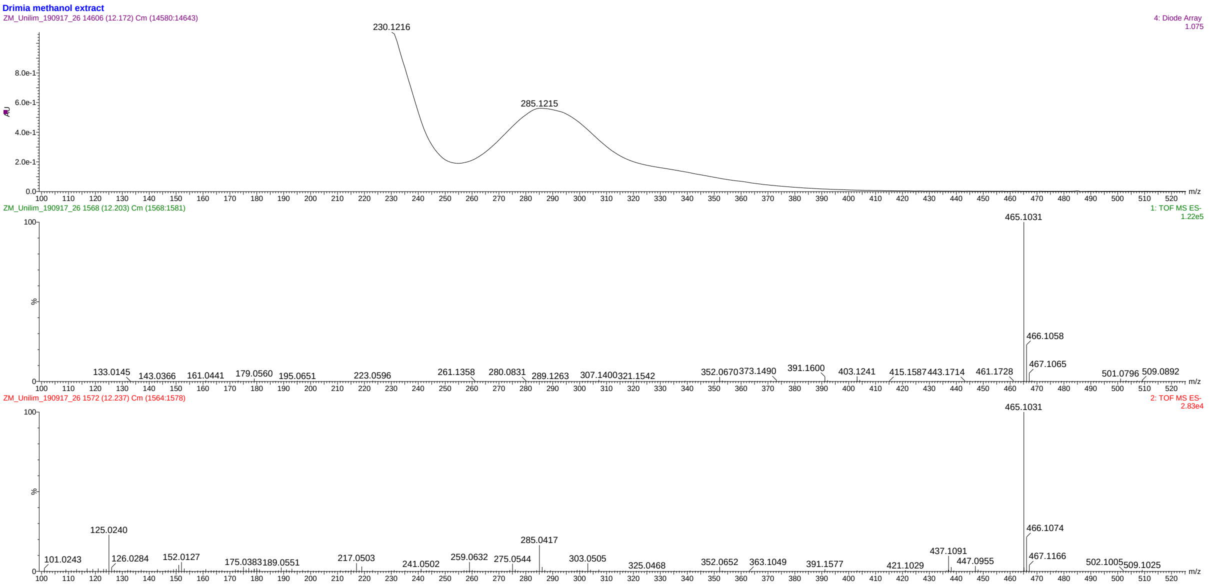

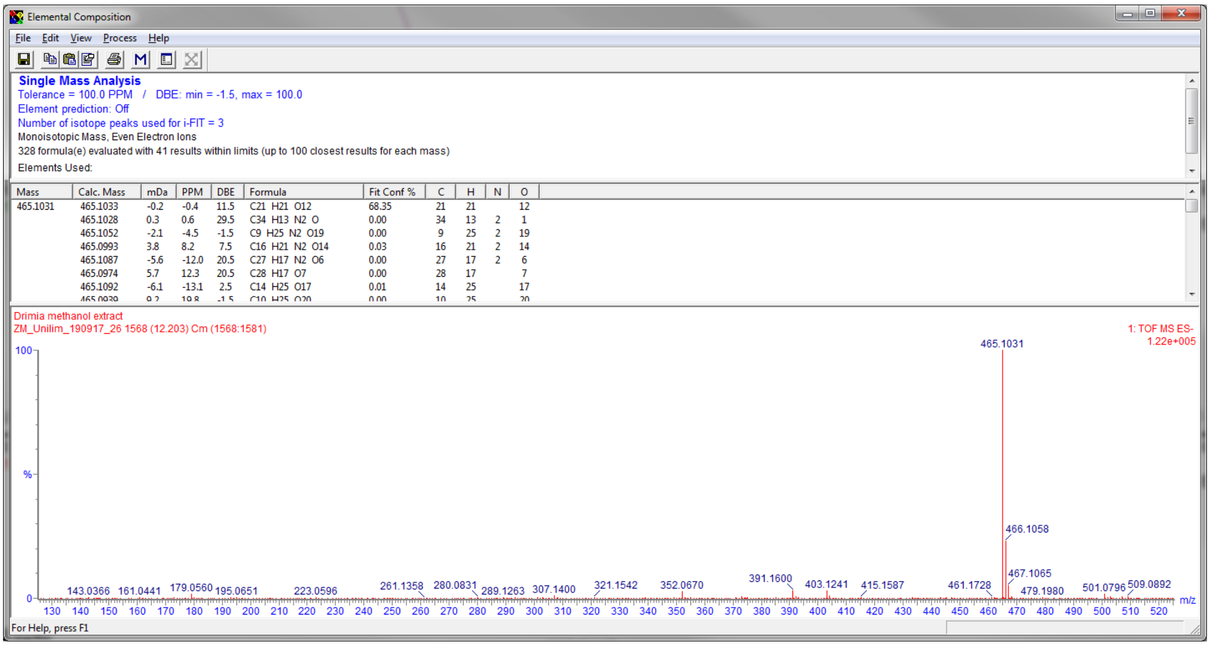

Mass	Calc. Mass	mDa	PPM	DBE	Formula	Fit Conf %	C	H	N	O
465.1031	465.1033	-0.2	-0.4	11.5	C21 H21 O12	68.35	21	21		12
	465.1028	0.3	0.6	29.5	C34 H13 N2 O	0.00	34	13	2	1
	465.1052	-2.1	-4.5	-1.5	C9 H25 N2 O19	0.00	9	25	2	19
	465.0993	3.8	8.2	7.5	C16 H21 N2 O14	0.03	16	21	2	14
	465.1087	-5.6	-12.0	20.5	C27 H17 N2 O6	0.00	27	17	2	6
	465.0974	5.7	12.3	20.5	C28 H17 O7	0.00	28	17		7
	465.1092	-6.1	-13.1	2.5	C14 H25 O17	0.01	14	25		17
	465.0939	9.2	19.8	-1.5	C10 H25 O20	0.00	10	25		20

Dihydromyricetin 3-O-rhamnoside

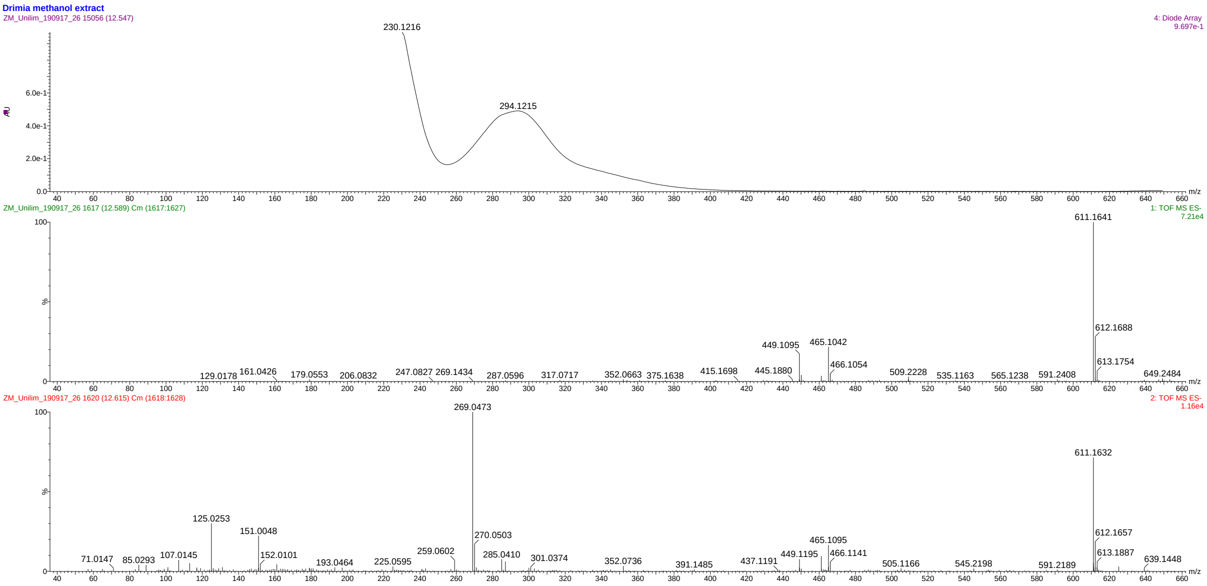

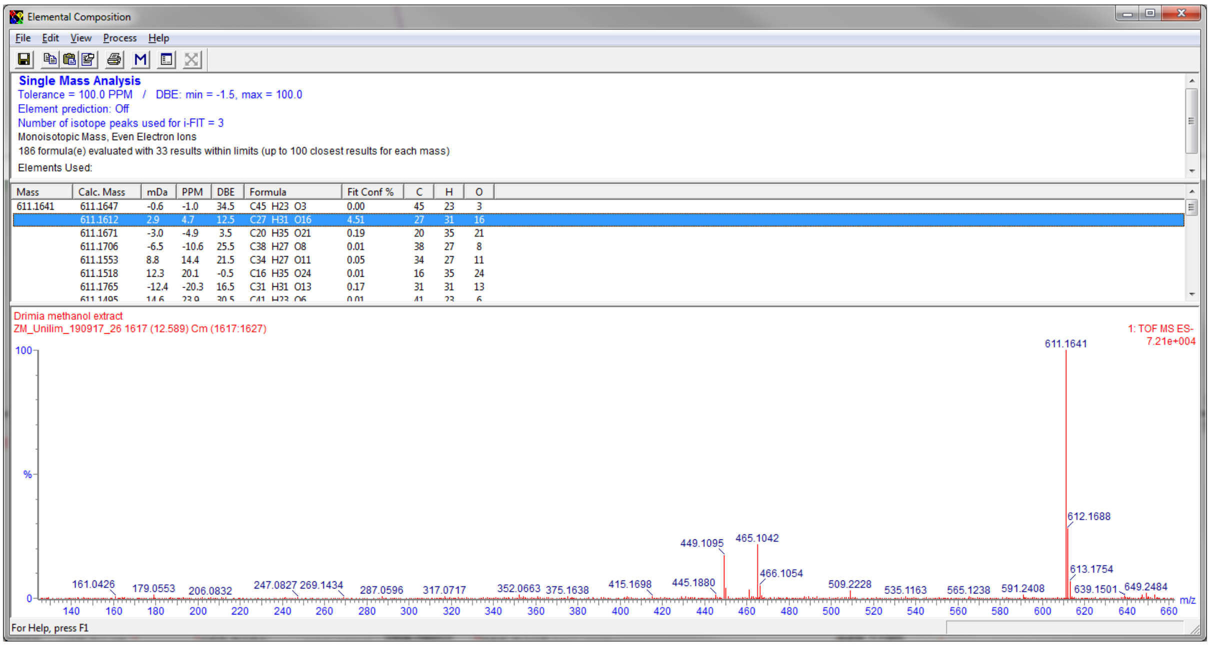

Mass	Calc. Mass	mDa	PPM	DBE	Formula	Fit Conf %	C	H	O
611.1641	611.1647	-0.6	-1.0	34.5	C45 H23 O3	0.00	45	23	3
	611.1612	2.9	4.7	12.5	C27 H31 O16	4.51	27	31	16
	611.1671	-3.0	-4.9	3.5	C20 H35 O21	0.19	20	35	21
	611.1706	-6.5	-10.6	25.5	C38 H27 O8	0.01	38	27	8
	611.1553	8.8	14.4	21.5	C34 H27 O11	0.05	34	27	11
	611.1518	12.3	20.1	-0.5	C16 H35 O24	0.01	16	35	24
	611.1765	-12.4	-20.3	16.5	C31 H31 O13	0.17	31	31	13
	611.1495	14.6	23.9	30.5	C41 H23 O6	0.01	41	23	6

Cyanidin 3-O-sophoroside (cyanidin fragment at m/z 287)

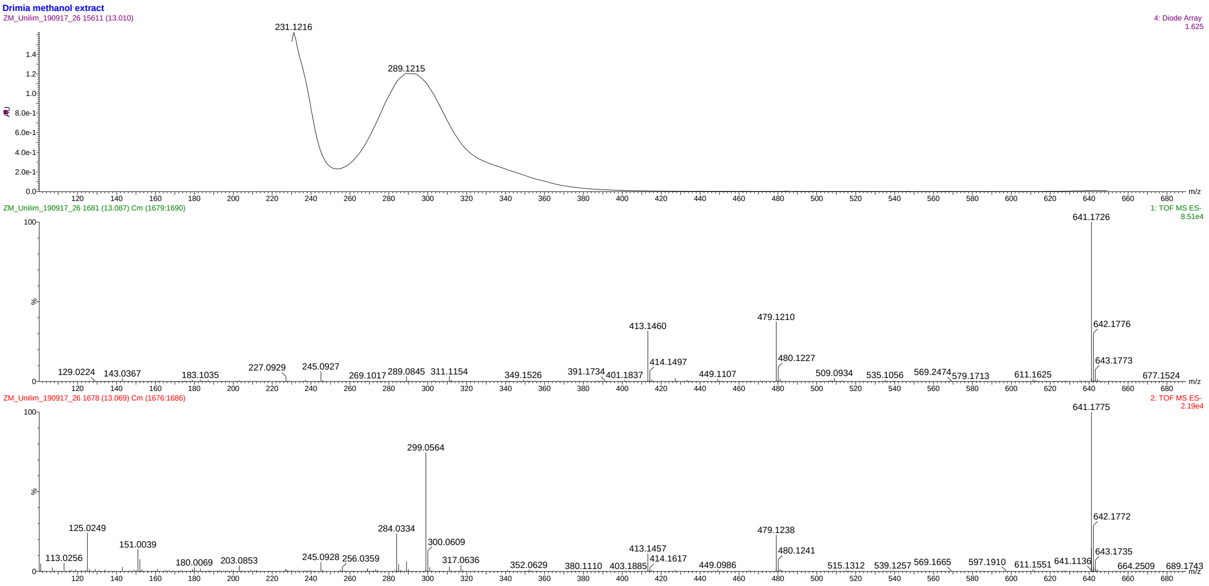

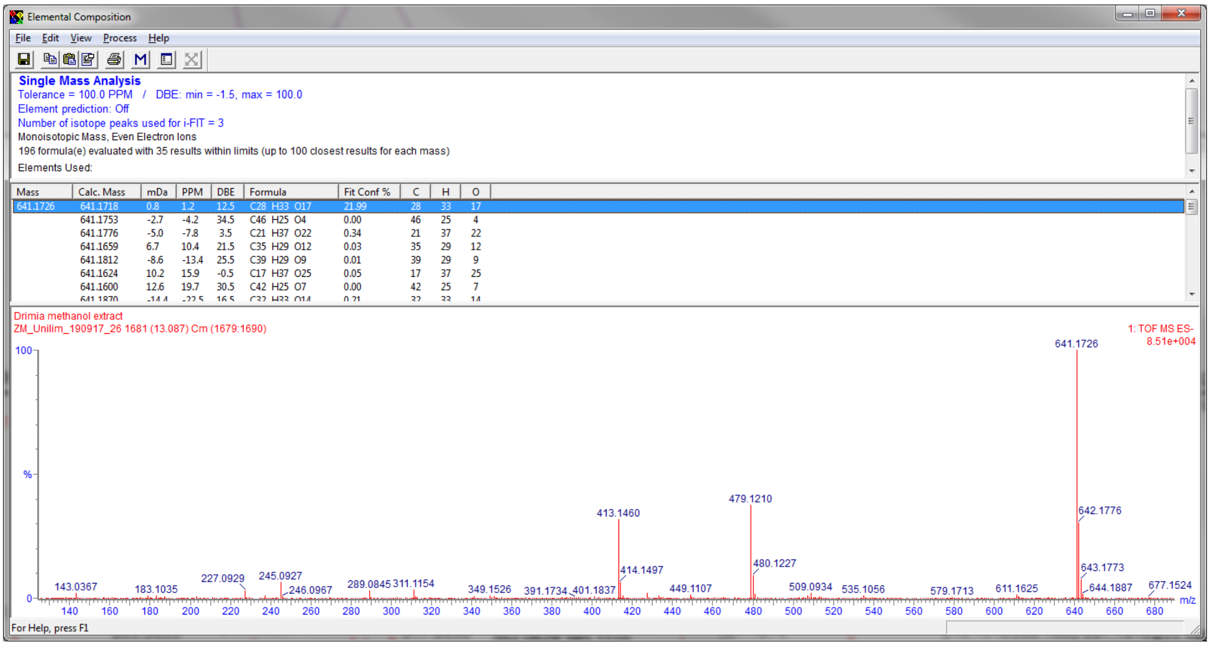
Elemental Composition

File Edit View Process Help

Single Mass Analysis
Tolerance = 100.0 PPM / DBE: min = -1.5, max = 100.0
Element prediction: Off
Number of isotope peaks used for i-FIT = 3
Monoisotopic Mass, Even Electron Ions
196 formula(e) evaluated with 35 results within limits (up to 100 closest results for each mass)
Elements Used:

Mass	Calc. Mass	mDa	PPM	DBE	Formula	Fit Conf %	C	H	O
641.1726	641.1718	0.8	1.2	12.5	C28 H33 O17	21.99	28	33	17
	641.1753	-2.7	-4.2	34.5	C46 H25 O4	0.00	46	25	4
	641.1776	-5.0	-7.8	3.5	C21 H37 O22	0.34	21	37	22
	641.1659	6.7	10.4	21.5	C35 H29 O12	0.03	35	29	12
	641.1812	-8.6	-13.4	25.5	C39 H29 O9	0.01	39	29	9
	641.1624	10.2	15.9	-0.5	C17 H37 O25	0.05	17	37	25
	641.1600	12.6	19.7	30.5	C42 H25 O7	0.00	42	25	7
	641.1870	-14.4	-22.5	16.5	C32 H33 O14	0.21	32	33	14

Petunidin 3,5-O-diglucoside

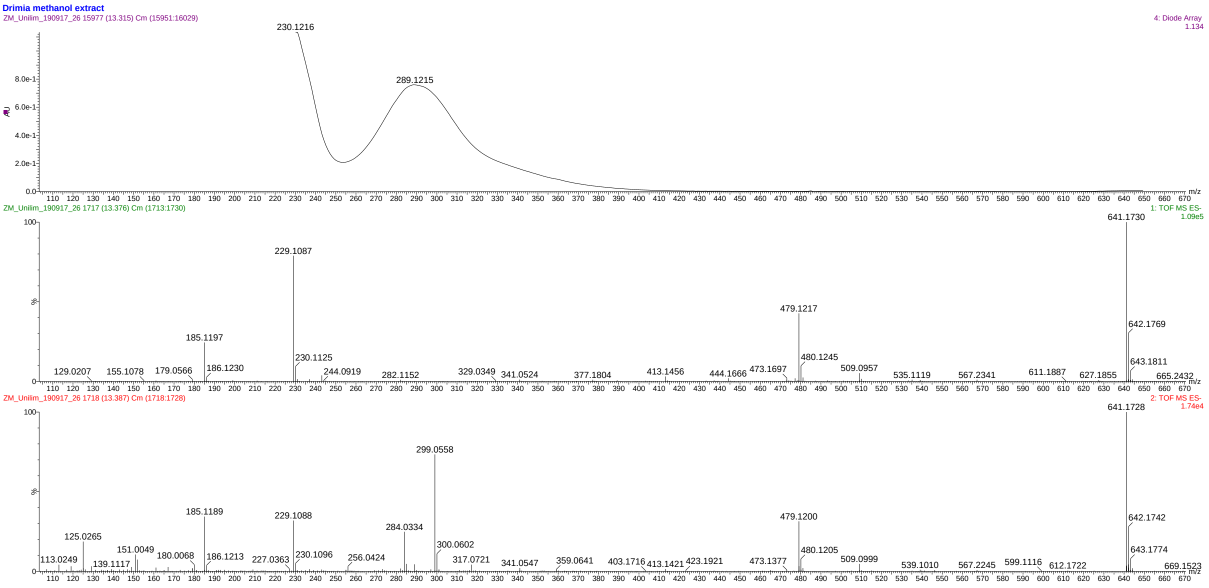

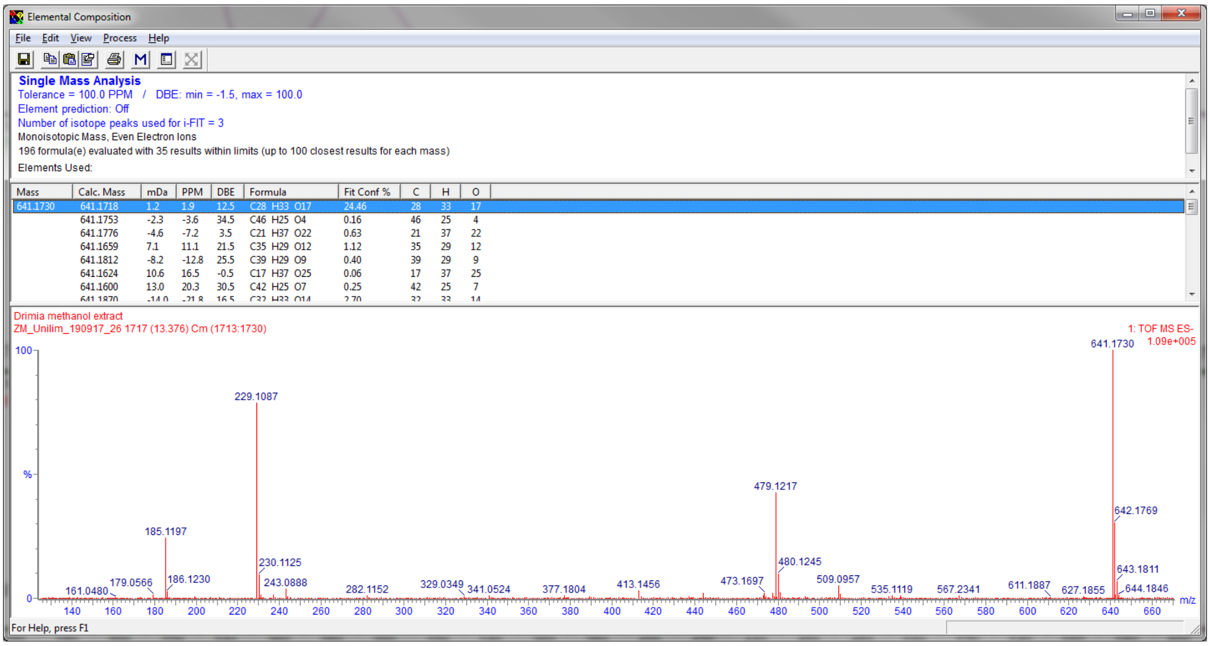

Mass	Calc. Mass	mDa	PPM	DBE	Formula	Fit Conf %	C	H	O
641.1730	641.1718	1.2	1.9	12.5	C28 H33 O17	24.46	28	33	17
	641.1753	-2.3	-3.6	34.5	C46 H25 O4	0.16	46	25	4
	641.1776	-4.6	-7.2	3.5	C21 H37 O22	0.63	21	37	22
	641.1659	7.1	11.1	21.5	C35 H29 O12	1.12	35	29	12
	641.1812	-8.2	-12.8	25.5	C39 H29 O9	0.40	39	29	9
	641.1624	10.6	16.5	-0.5	C17 H37 O25	0.06	17	37	25
	641.1600	13.0	20.3	30.5	C42 H25 O7	0.25	42	25	7
	641.1870	-14.0	-21.8	16.5	C32 H33 O14	2.70	32	33	14

Petunidin O-diglucoside isomer

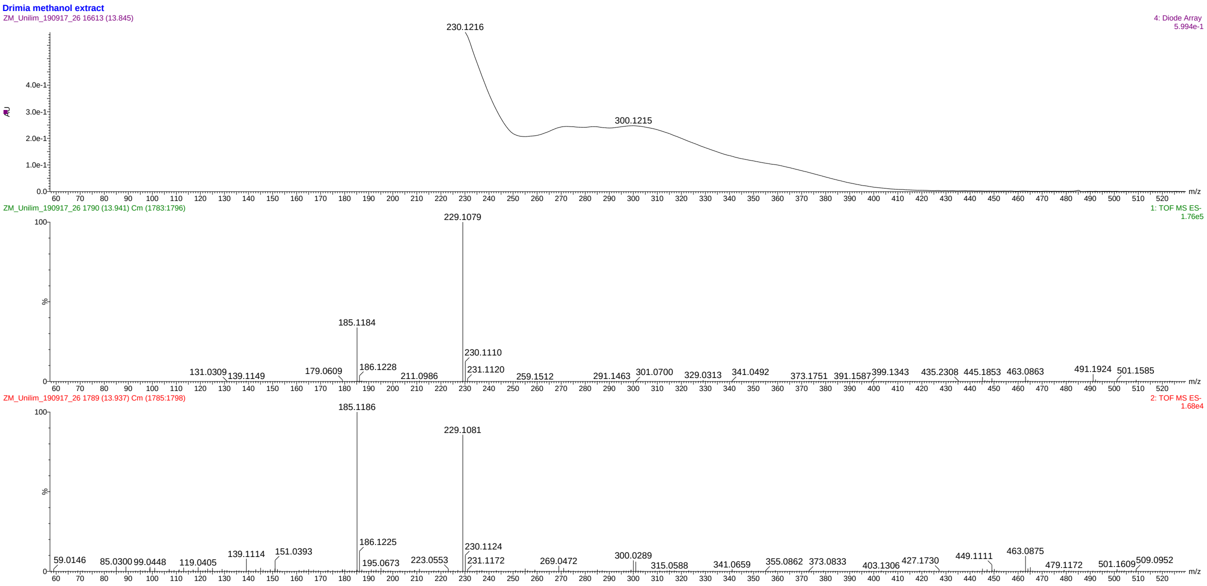

Elemental Composition

File Edit View Process Help

Single Mass Analysis

Tolerance = 100.0 PPM / DBE: min = -1.5, max = 100.0

Element prediction: Off

Number of isotope peaks used for i-FIT = 3

Monoisotopic Mass, Even Electron Ions

114 formula(e) evaluated with 22 results within limits (up to 100 closest results for each mass)

Elements Used:

Mass	Calc. Mass	mDa	PPM	DBE	Formula	Fit Conf %	C	H	O
	463.1147	-27.2	-58.7	-1.5	C11 H27 O19	2.33	11	27	19
	463.0571	30.4	65.6	4.5	C13 H19 O18	3.01	13	19	18
	463.1182	-30.7	-66.3	20.5	C29 H19 O6	4.62	29	19	6
	463.0548	32.7	70.6	35.5	C38 H7	4.35	38	7	
	463.0513	36.2	78.2	13.5	C20 H15 O13	4.15	20	15	13
	463.1240	-36.5	-78.8	11.5	C22 H23 O11	3.80	22	23	11
	463.0454	42.1	90.9	22.5	C27 H11 O8	3.93	27	11	8
	463.1299	-42.4	-91.6	2.5	C15 H27 O16	2.51	15	27	16

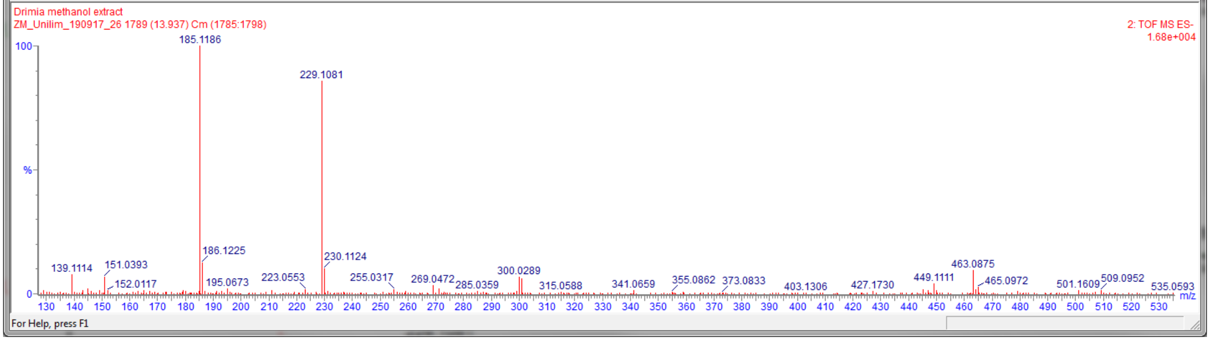

For Help, press F1

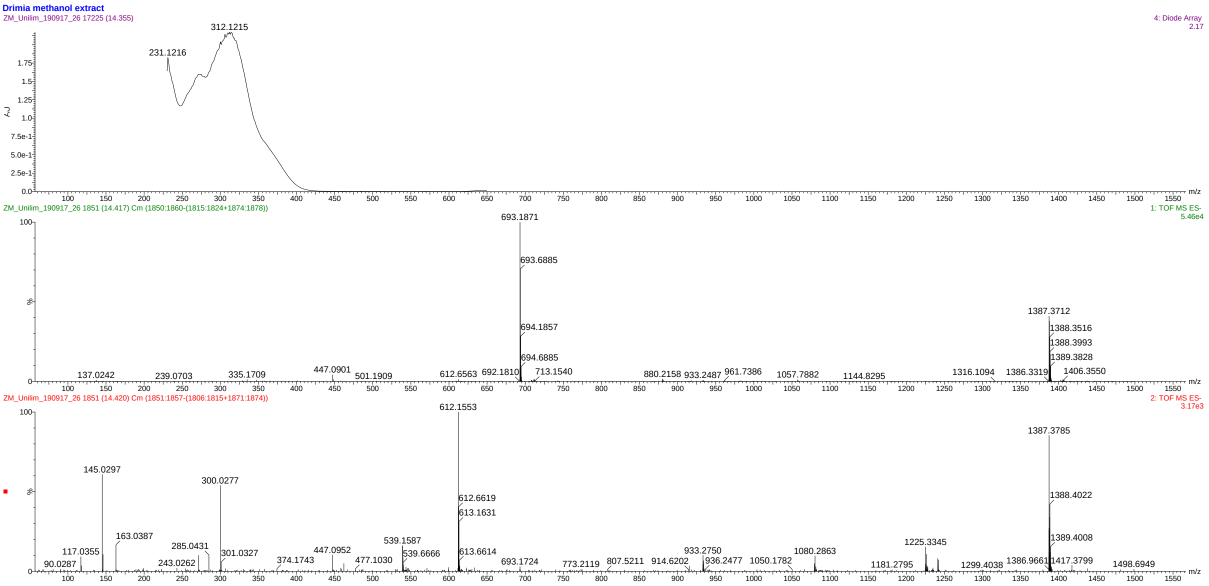

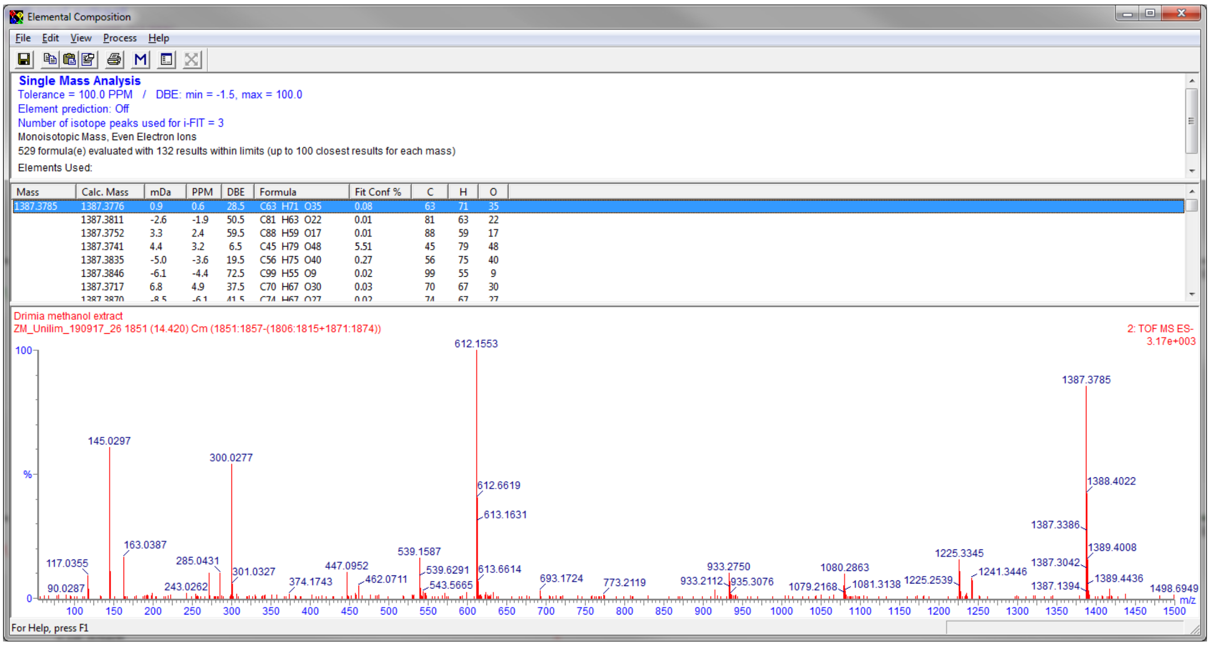

Mass	Calc. Mass	mDa	PPM	DBE	Formula	Fit Conf %	C	H	O
1387.3785	1387.3776	0.9	0.6	28.5	C63 H71 O35	0.08	63	71	35
	1387.3811	-2.6	-1.9	50.5	C81 H63 O22	0.01	81	63	22
	1387.3752	3.3	2.4	59.5	C88 H59 O17	0.01	88	59	17
	1387.3741	4.4	3.2	6.5	C45 H79 O48	5.51	45	79	48
	1387.3835	-5.0	-3.6	19.5	C56 H75 O40	0.27	56	75	40
	1387.3846	-6.1	-4.4	72.5	C99 H55 O9	0.02	99	55	9
	1387.3717	6.8	4.9	37.5	C70 H67 O30	0.03	70	67	30
	1387.3870	-8.5	-6.1	41.5	C74 H67 O27	0.02	74	67	27

Unknown

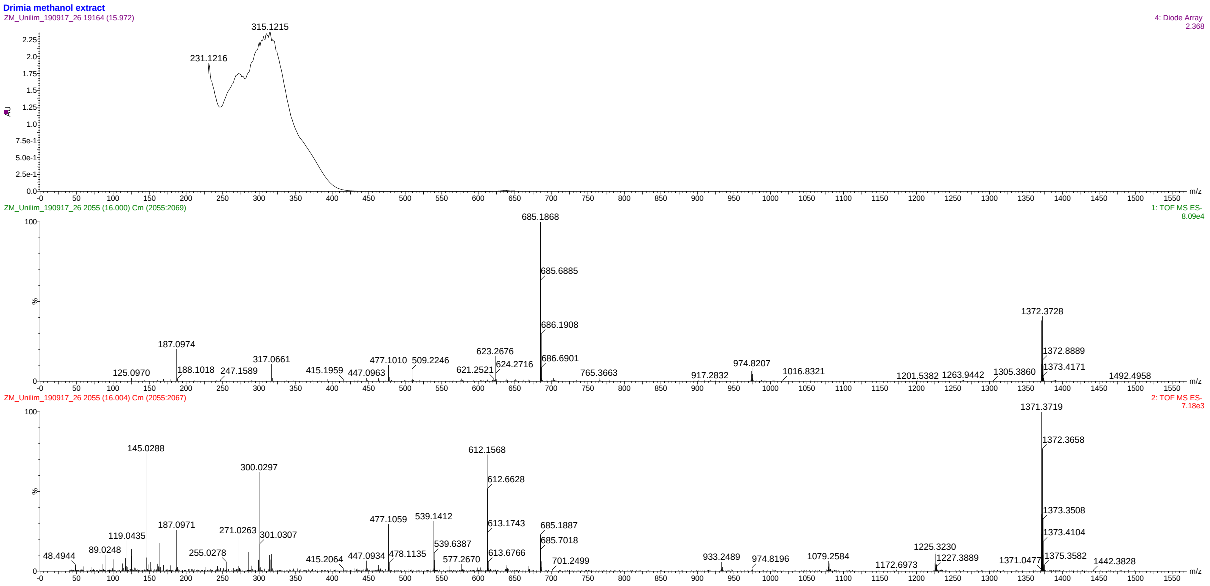

Elemental Composition

File Edit View Process Help

Single Mass Analysis

Tolerance = 100.0 PPM / DBE: min = -1.5, max = 100.0

Element prediction: Off

Number of isotope peaks used for i-FIT = 3

Monoisotopic Mass, Even Electron Ions

529 formula(e) evaluated with 133 results within limits (up to 100 closest results for each mass)

Elements Used:

Mass	Calc. Mass	mDa	PPM	DBE	Formula	Fit Conf %	C	H	O
1371.3763	1371.3768	-0.5	-0.4	37.5	$C_{70}H_{67}O_{29}$	2.10	70	67	29
	1371.3744	1.9	1.4	68.5	$C_{95}H_{55}O_{11}$	0.48	95	55	11
	1371.3792	-2.9	-2.1	6.5	$C_{45}H_{79}O_{47}$	0.58	45	79	47
	1371.3733	3.0	2.2	15.5	$C_{52}H_{75}O_{42}$	1.02	52	75	42
	1371.3803	-4.0	-2.9	59.5	$C_{88}H_{59}O_{16}$	0.80	88	59	16
	1371.3709	5.4	3.9	46.5	$C_{77}H_{63}O_{24}$	1.63	77	63	24
	1371.3827	-6.4	-4.7	28.5	$C_{63}H_{71}O_{34}$	1.96	63	71	34
	1371.3674	8.9	6.5	24.5	$C_{59}H_{71}O_{37}$	1.65	59	71	37

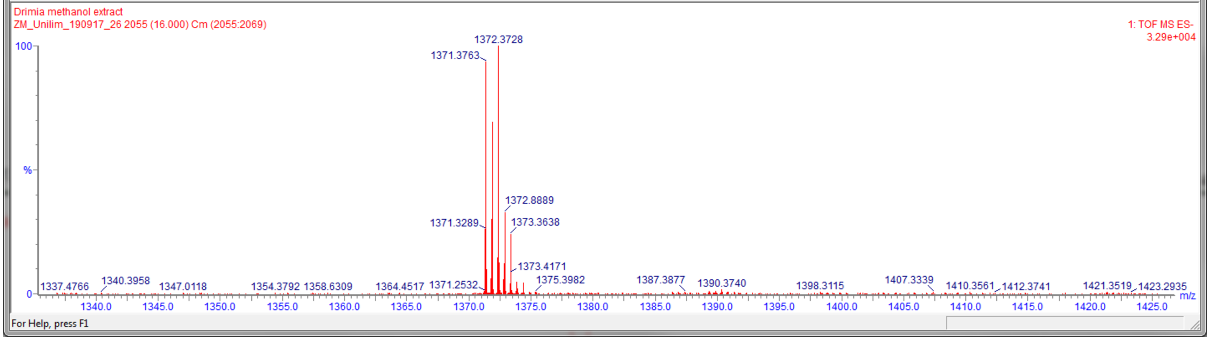

For Help, press F1

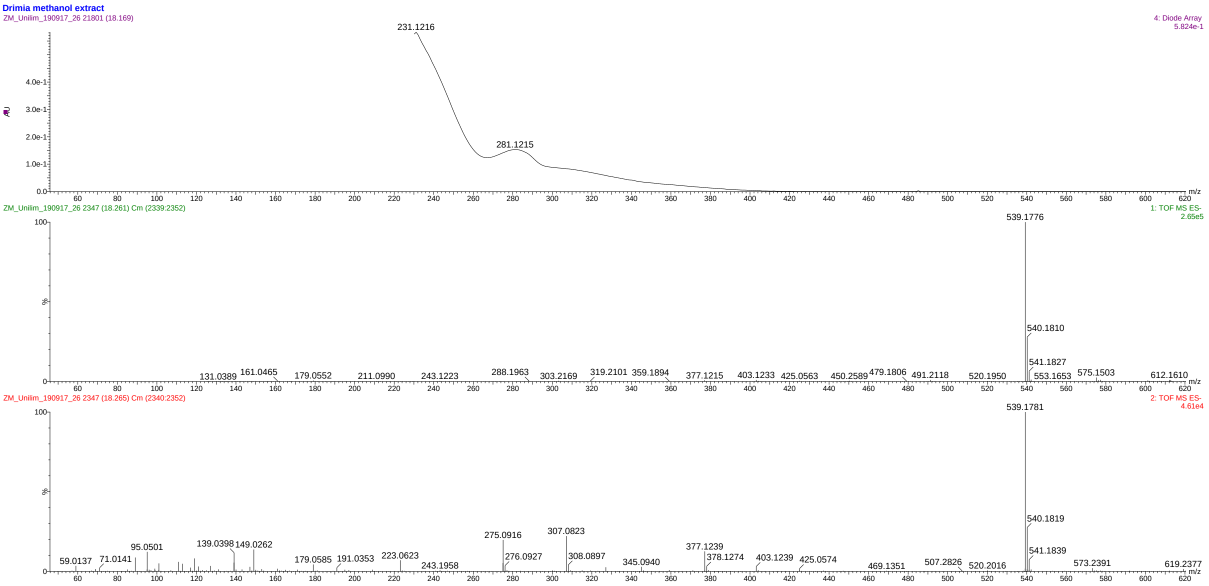
Drimia methanol extract
ZM_Unilim_190917_26 21801 (18.169)
4: Diode Array
5.824e-1
231.1216
281.1215
ZM_Unilim_190917_26 2347 (18.261) Cm (2339:2352)
1: TOF MS ES-
2.65e5
539.1776
540.1810
541.1827
ZM_Unilim_190917_26 2347 (18.265) Cm (2340:2352)
2: TOF MS ES-
4.61e4
539.1781
540.1819
541.1839
m/z

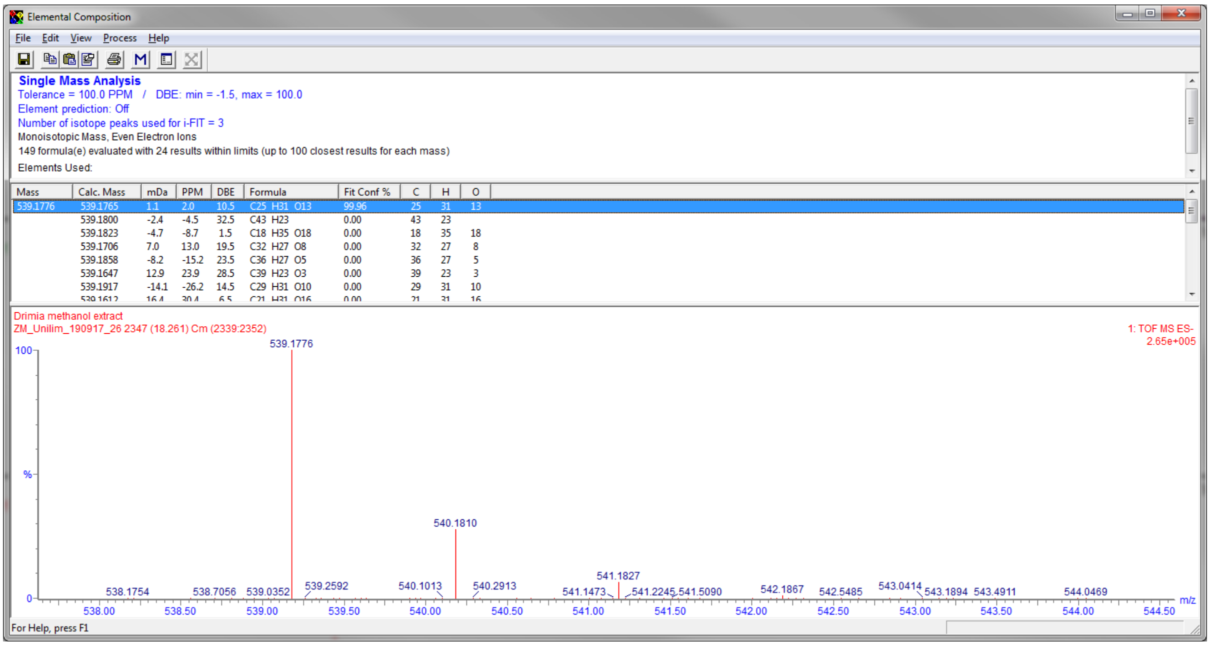
Elemental Composition
File Edit View Process Help
Single Mass Analysis
Tolerance = 100.0 PPM / DBE: min = -1.5, max = 100.0
Element prediction: Off
Number of isotope peaks used for i-FIT = 3
Monoisotopic Mass, Even Electron Ions
149 formula(e) evaluated with 24 results within limits (up to 100 closest results for each mass)
Elements Used:
Mass Calc. Mass mDa PPM DBE Formula Fit Conf % C H O
539.1776 539.1765 1.1 2.0 10.5 C25 H31 O13 99.96 25 31 13
539.1800 -2.4 -4.5 32.5 C43 H23 0.00 43 23
539.1823 -4.7 -8.7 1.5 C18 H35 O18 0.00 18 35 18
539.1706 7.0 13.0 19.5 C32 H27 O8 0.00 32 27 8
539.1858 -8.2 -15.2 23.5 C36 H27 O5 0.00 36 27 5
539.1647 12.9 23.9 28.5 C39 H23 O3 0.00 39 23 3
539.1917 -14.1 -26.2 14.5 C29 H31 O10 0.00 29 31 10
Drimia methanol extract
ZM_Unilim_190917_26 2347 (18.261) Cm (2339:2352)
1: TOF MS ES-
2.65e+005
539.1776
540.1810
541.1827
538.1754
538.7056
539.0352
539.2592
540.1013
540.2913
541.1473
541.2245
541.5090
542.1867
542.5485
543.0414
543.1894
543.4911
544.0469
m/z
For Help, press F1

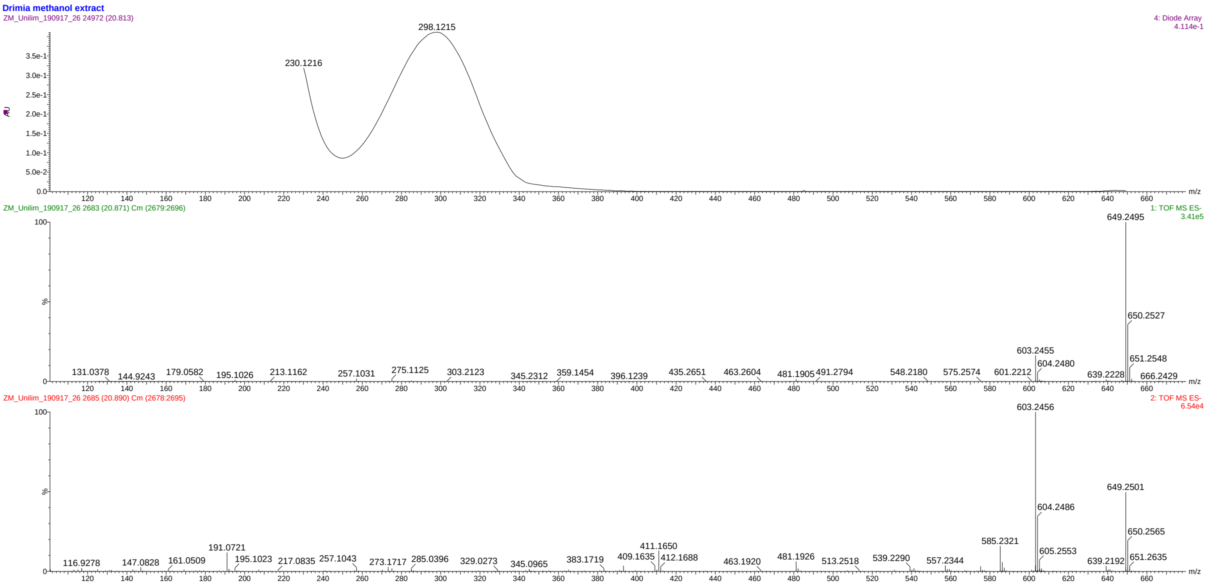

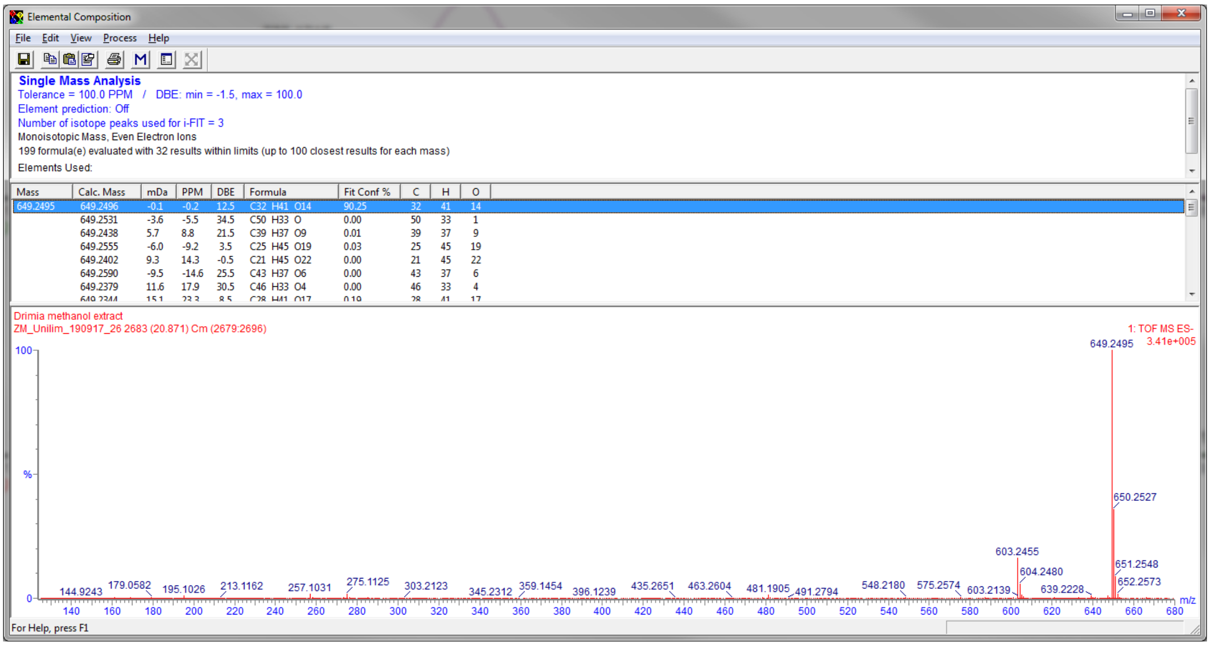

Mass	Calc. Mass	mDa	PPM	DBE	Formula	Fit Conf %	C	H	O
649.2495	649.2496	-0.1	-0.2	12.5	C32 H41 O14	90.25	32	41	14
	649.2531	-3.6	-5.5	34.5	C50 H33 O	0.00	50	33	1
	649.2438	5.7	8.8	21.5	C39 H37 O9	0.01	39	37	9
	649.2555	-6.0	-9.2	3.5	C25 H45 O19	0.03	25	45	19
	649.2402	9.3	14.3	-0.5	C21 H45 O22	0.00	21	45	22
	649.2590	-9.5	-14.6	25.5	C43 H37 O6	0.00	43	37	6
	649.2379	11.6	17.9	30.5	C46 H33 O4	0.00	46	33	4
	649.2344	15.1	23.3	8.5	C28 H41 O17	0.19	28	41	17

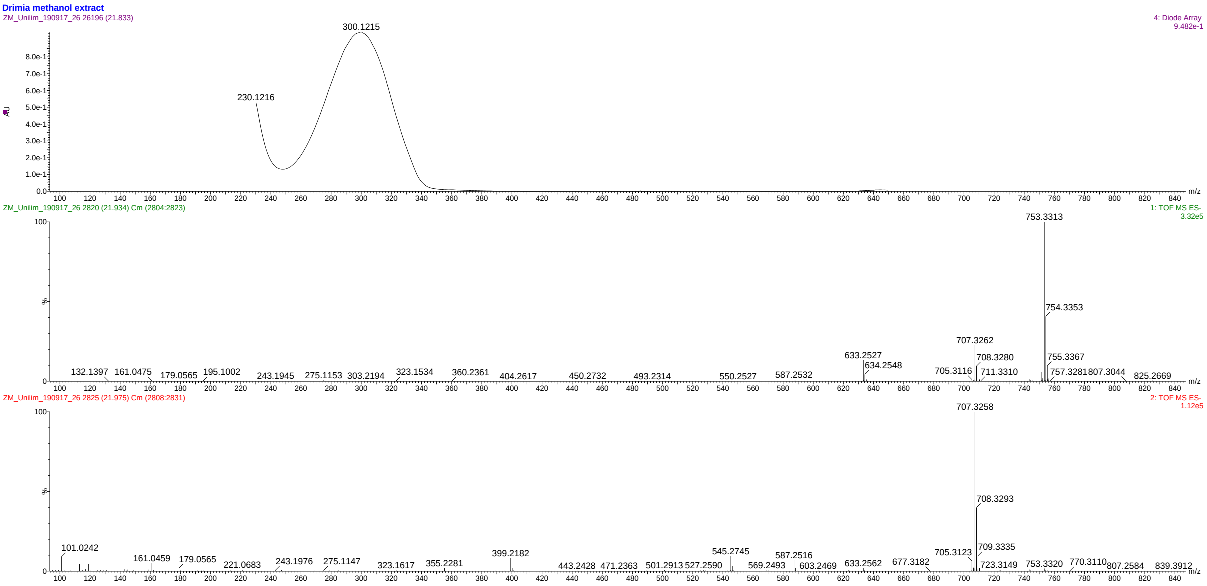

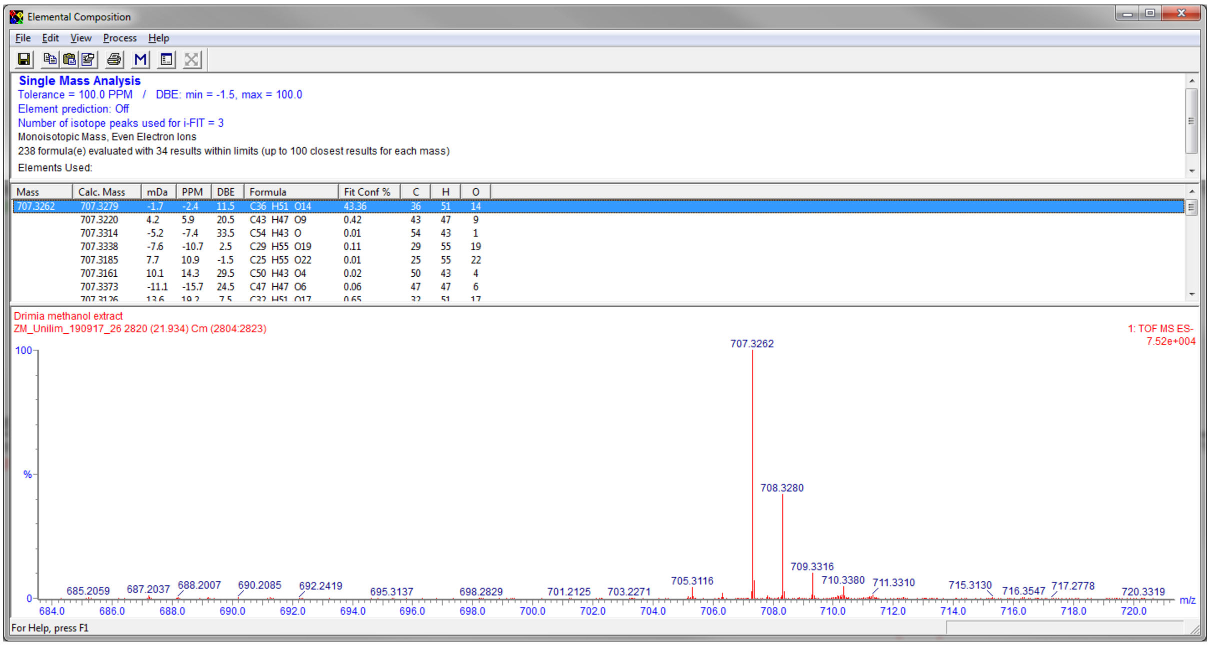

m/z 753 is the formate adducte of 707

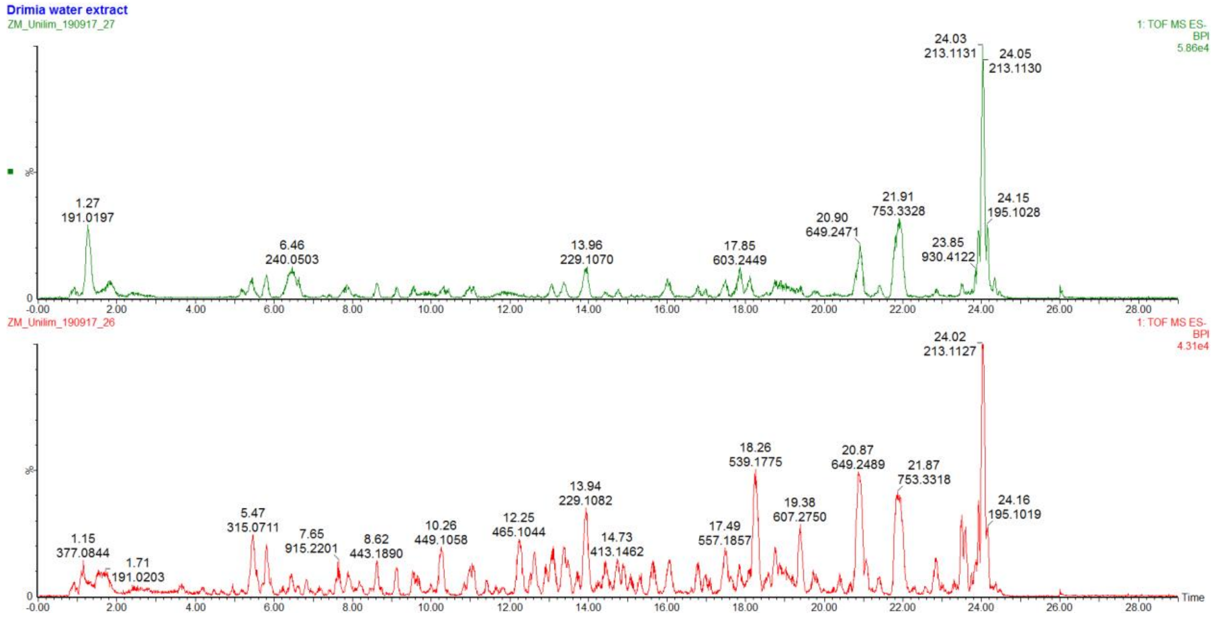

Water (top) vs methanol (bottom) extracts of Drimia

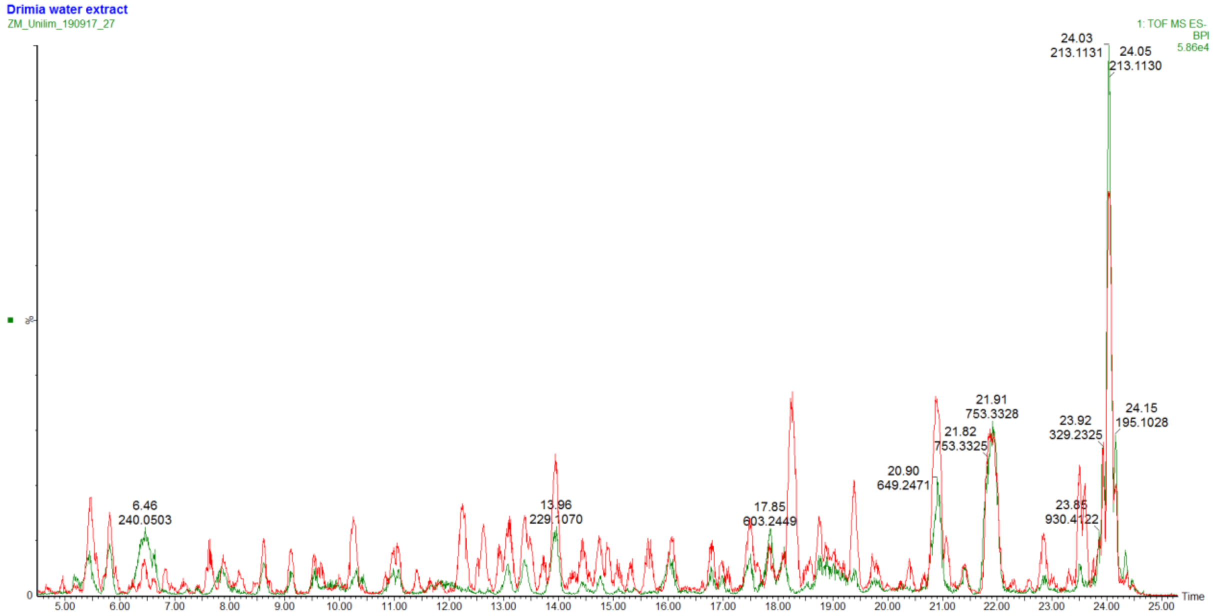

Overlay of water and methanol extracts

Figure S12: The LC-MS chromatograms of *D. calcarata* water and methanol fractions.

MTT assay (Water Fractions)

HEK-293 Cells

Table S24: The MTT assay average percentages and standard error of mean (SEM) of HEK-293 cells after 24 h treatment with *D. calcarata* water fractions.

**Treatment (µg/mL)**	**Mean (%) ±SEM**			
	**Fraction 1**	**Fraction 2**	**Fraction 3**	**Fraction 4**
**0**	100.00±0.000	100.00±0.000	100.00±0.000	100.00±0.000
**0.25% H_2O**	103.77±1.884	103.77±1.884	103.77±1.884	103.77±1.884
**50 µM Curcumin**	106.57±2.202	106.57±2.202	106.57±2.202	106.57±2.202
**15.63**	119.20±0.586	121.78±1.213	136.93±2.577	139.43±1867
**31.25**	115.70±1.480	125.18±0.952	110.17±9.117	126.57±0.524
**62.50**	93.40±1.068	105.60±1.192	113.73±1.257	107.83±4.807
**125**	75.90±3.950	103.70±1.089	115.93±2.339	109.90±0.322
**250**	63.50±3.134	90.03±2.212	105.40±4.373	75.43±2.035
**500**	31.80±1.060	82.00±2.322	109.27±1.832	77.90±2.511
**1000**	30.43±0.470	51.03±0.838	57.27±1.357	91.00±0.289

A549 cells

Table S25: The MTT assay average percentages and standard error of mean (SEM) of A549 cells after 24 h treatment with *D. calcarata* water fractions.

**Treatment (µg/mL)**	**Mean (%) ±SEM**			
	**Fraction 1**	**Fraction 2**	**Fraction 3**	**Fraction 4**
**0**	100.00±0.000	100.00±0.000	100.00±0.000	100.00±0.000
**0.25% H_2O**	114.88±1.125	114.88±1.125	114.88±1.125	114.88±1.125
**50 µM Curcumin**	50.625±0.375	50.625±0.375	50.625±0.375	50.625±0.375
**15.63**	87.500±0.926	51.750±0.881	72.625±2.195	87.875±4.029
**31.25**	83.375±0.800	51.250±0.526	70.750±1.521	68.125±3.446
**62.50**	79.000±1.165	79.000±0.707	68.500±1.363	54.875±2.310

**125**	81.250±1.509	48.875±0.667	67.875±2.326	48.000±1.842
**250**	76.857±2.310	47.125±0.742	60.625±1.164	52.000±2.514
**500**	21.750±0.675	26.250±0.620	48.250±0.940	45.875±2.567
**1000**	18.625±0.596	19.875±0.743	34.875±0.990	45.250±2.717

MTT assay (Methanol Fractions)

HEK-293 cells

Table S26: The MTT assay average percentages and standard error of mean (SEM) of HEK-293 cells after 24 h treatment with *D. calcarata* methanol fractions.

**Treatment (µg/mL)**	**Mean (%) ±SEM**		
	**Fraction 1**	**Fraction 2**	**Fraction 3**
**0**	100.00±0.000	100.00±0.000	100.00±0.000
**0.25% DMSO**	103.77±1.884	103.77±1.884	103.77±1.884
**50 µM Curcumin**	106.57±2.202	106.57±2.202	106.57±2.202
**15.63**	159.57±2.976	95.87±1.994	101.13±0.974
**31.25**	155.97±1.922	87.30±2.608	96.03±1.938
**62.50**	118.10±3.460	77.63±3.023	81.33±1.827
**125**	104.90±2.994	78.90±2.779	59.50±1.343
**250**	98.23±1.846	71.83±0.601	56.17±0.561
**500**	91.60±8.137	48.40±0.529	51.30±0.839
**1000**	49.60±1.200	46.40±1.200	42.77±0.291

A549 cells

Table S27: The MTT assay average percentages and standard error of mean (SEM) of A549 cells after 24 h treatment with *D. calcarata* methanol fractions.

**Treatment (µg/mL)**	**Mean (%) ±SEM**		
	**Fraction 1**	**Fraction 2**	**Fraction 3**
**0**	100.00±0.000	100.00±0.000	100.00±0.000
**0.25% DMSO**	111.38±2.492	111.38±2.492	111.38±2.492

**50 µM Curcumin**	51.000±0.327	51.000±0.327	51.000±0.327
**15.63**	98.625±2.434	38.375±0.730	77.375±2.738
**31.25**	88.500±3.620	39.250±0.700	76.375±1.580
**62.50**	64.875±1.807	40.500±0.823	77.750±1.386
**125**	35.875±0.934	43.250±1.461	72.125±2.013
**250**	37.250±0.796	44.750±1.497	67.125±1.986
**500**	1.875±0.125	33.375±1.017	66.750±1.623
**1000**	2.000±0.000	27.625±1.592	39.375±1.388
